# Capturing the microbial dark matter in desert soils using culturomics-based metagenomics and high-resolution analysis

**DOI:** 10.1038/s41522-023-00439-8

**Published:** 2023-09-22

**Authors:** Shuai Li, Wen-Hui Lian, Jia-Rui Han, Mukhtiar Ali, Zhi-Liang Lin, Yong-Hong Liu, Li Li, Dong-Ya Zhang, Xian-Zhi Jiang, Wen-Jun Li, Lei Dong

**Affiliations:** 1 State Key Laboratory of Biocontrol, Guangdong Provincial Key Laboratory of Plant Resources and Southern Marine Science and Engineering Guangdong Laboratory (Zhuhai), School of Life Sciences, Sun Yat‑sen University, Guangzhou, 510275 China; 2 School of Life Science, Jiaying University, Meizhou, 514015 China; 3 State Key Laboratory of Desert and Oasis Ecology, Xinjiang Institute of Ecology and Geography, Chinese Academy of Sciences, Urumqi, 830011 China; 4 Microbiome Research Center, Moon (Guangzhou) Biotech Ltd., Guangzhou, 510700 China

**Keywords:** Metagenomics, Microbiome

## Abstract

Deserts occupy one-third of the Earth’s terrestrial surface and represent a potentially significant reservoir of microbial biodiversity, yet the majority of desert microorganisms remain uncharacterized and are seen as “microbial dark matter”. Here, we introduce a multi-omics strategy, culturomics-based metagenomics (CBM) that integrates large-scale cultivation, full-length 16S rRNA gene amplicon, and shotgun metagenomic sequencing. The results showed that CBM captured a significant amount of taxonomic and functional diversity missed in direct sequencing by increasing the recovery of amplicon sequence variants (ASVs) and high/medium-quality metagenome-assembled genomes (MAGs). Importantly, CBM allowed the post hoc recovery of microbes of interest (e.g., novel or specific taxa), even those with extremely low abundance in the culture. Furthermore, strain-level analyses based on CBM and direct sequencing revealed that the desert soils harbored a considerable number of novel bacterial candidates (1941, 51.4%), of which 1095 (from CBM) were culturable. However, CBM would not exactly reflect the relative abundance of true microbial composition and functional pathways in the in situ environment, and its use coupled with direct metagenomic sequencing could provide greater insight into desert microbiomes. Overall, this study exemplifies the CBM strategy with high-resolution is an ideal way to deeply explore the untapped novel bacterial resources in desert soils, and substantially expands our knowledge on the microbial dark matter hidden in the vast expanse of deserts.

## Introduction

Deserts are among the largest and most understudied biomes on Earth, covering about one-third of the total global land surface^1^, and represent a significant reservoir of Earth’s microbial diversity^2^. Desert microorganisms play important roles in maintaining ecological stability and biogeochemical cycles. The profiling of microbial biodiversity, compositions, and functions of the desert ecosystems would help to understand global change, threats, and opportunities posed by life in drylands^3^. Furthermore, many studies have suggested that the diverse microbial communities in desert habitats can produce an impressive array of novel bioactive compounds, including antimicrobial, anti-inflammatory, anti-tumor and anti-quorum sensing candidates, etc.^4–8^. However, due to strong niche specialization, difficulty in sampling, and limited adaptability of conventional culture methods, most of the desert microorganisms have been neglected to be cultivated and characterized in the laboratory^9^. This large and as yet poorly explored portion of microbial diversity represents a vast underexplored and uncharacterized biological resource, colloquially called “microbial dark matter”^10,11^, which represents a fundamental impediment to microbial ecology and bioresource exploitation^12,13^.

Metagenomic approaches (e.g., 16S amplicon and shotgun sequencing) provide relatively simple and rapid ways to profile the taxonomic composition and functional potential of microbial community and to recover whole genome sequences without the necessity of culturing^14^. Recent metagenomic surveys on desert microbiomes have significantly advanced our current understanding of the composition and function of microbial populations in the global deserts^15–17^, such as the Atacama Desert^18–21^, Namib Desert^22–24^, Negev Desert^25,26^, Gurbantunggut Desert^27^ and polar deserts^28,29^, laying an important foundation for further in-depth exploration of desert microbial resources. However, the usefulness of metagenomic data from environmental samples is highly dependent on the complexity and biomass of the community, sequencing technology and reference database, etc.^30–32^, which may result in the omission of certain specific taxa, such as those with low abundance.

Culturomics, which applies multiple culture conditions in combination with 16S rRNA gene amplicon sequencing and/or other technologies, has greatly improved our understanding of the diversity of culturable microbes^33,34^. Although culturomics has great utility in obtaining pure cultures of microbes, it is often considered labor- and resource-intensive approach and it may omit the specific target groups of importance within the microbial community^35^. Therefore, the generation of comprehensive strain collections via systematic culturomics is still an important and unresolved challenge^36^.

It is noteworthy that strategies for selective culture enrichment to reduce community complexity may aid the metagenomic studies in specific environmental biomes^14^ (e.g., gut^37,38^). However, no studies have jointly employed culturomics and culture-enriched metagenomic sequencing to study the desert microbiome. Given the available knowledge, we hypothesize that culture-enriched metagenomic sequencing based on a large-scale culturomic approach and high-resolution analysis enables largely to fill in the missing parts of the soil microbiome in direct sequencing. Taking this into count, our major aim is to evaluate the effectiveness and prospect of the multi-omics strategy combining culturomics and metagenomics in desert soil microbiome research, and provide new perspectives for exploring the microbial dark matter in desert soils.

**Fig. 1:**
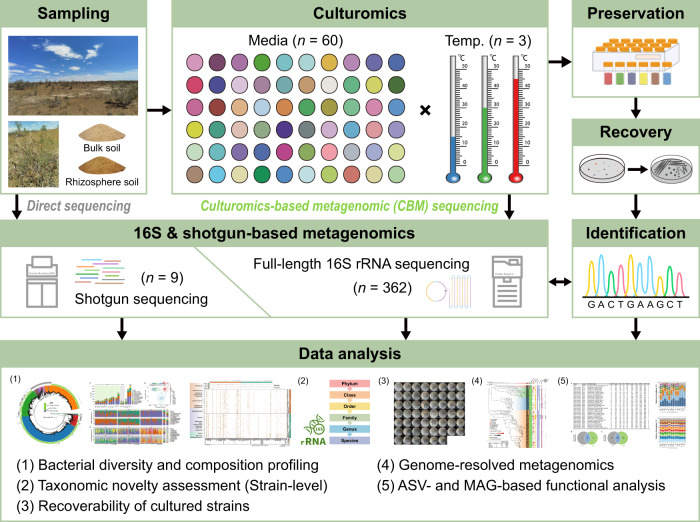
Schematic diagram of the experimental design. The bulk and rhizosphere soils (*n* = 2) of *Calligonum leucocladum* were selected by a pre-experiment (see Supplementary Fig. 10), then they were plated onto 60 different media and incubated at 15, 30 and 45 °C, of which generating 360 cultures within 6 subgroups: BCL (bulk soil cultures at 15 °C), BCM (bulk soil cultures at 30 °C), BCH (bulk soil cultures at 45 °C), RCL (rhizosphere cultures at 15 °C), RCM (rhizosphere cultures at 30 °C) and RCH (rhizosphere cultures at 45 °C). PacBio SMRT full-length 16S rRNA gene sequencing was conducted on the two original soil samples and the 360 culture-enriched samples. Also, shotgun metagenomic sequencing was performed on the two original soils and seven selected culture-enriched samples. Each culture-enriched sample was stored in glycerol (25%, v/v) at −80 °C, and two of which (BM11 and RM11 that cultured at 30 °C on M11 agar by bulk and rhizosphere soils, respectively) were used for the second-round restorative isolation.

Taking the mining of microbial dark matter in desert soils as an example, we present an integrated strategy that merges culturomic and metagenomic approaches (full-length 16S amplicon and shotgun sequencing), i.e., culturomics-based metagenomics (CBM) (Fig. 1). Our findings reveal the previously undescribed landscape of the untapped potential of novel bacterial resources in desert soils, and demonstrate the great advantages of CBM in increasing the taxonomic and functional resolution of the desert microbiome, which will expand knowledge of the microbial dark matter hidden in deserts.

## Results

### CBM captures the majority of ASVs missed by direct sequencing

A total of 4,610,948 circular consensus sequences (CCSs) were obtained via the full-length 16S rRNA gene sequencing of the two original soil samples (Bulk soil: 12,913; Rhizosphere soil: 12,980) and 360 culture-enriched samples (range from 8213 to 13,220). Of these, 3,666,324 effective CCSs were recovered after identifying the barcode sequences, quality filtering, denoising, and removing chimeras. Original bulk and rhizosphere soil samples were comprised of 6299 and 6328 effective CCSs, respectively. Whereas, culture-enriched samples consisted of an average of 10,121 effective CCSs, ranging from 6354 to 11,778. Across all 362 samples, a total of 3779 different ASVs were generated.

**Fig. 2:**
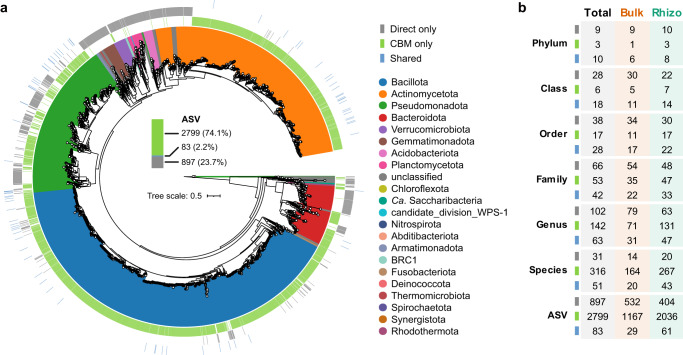
Numerical and taxonomic comparisons of ASVs recovered by direct sequencing and CBM. **a** ASV-based phylogenetic tree. 74.1% (2799) and 23.7% (897) of ASVs across the dataset were detected only in direct sequencing (gray) and CBM (green), respectively. The ASVs shared by direct sequencing and CBM accounted for just 2.2% (83, polo blue). **b** The number of bacterial taxa detected by direct sequencing and CBM at different taxonomic ranks. Bulk bulk soil, Rhizo rhizosphere soil.

The culturomics-based metagenomic (CBM) strategy based on full-length 16S rRNA gene sequencing recovered more ASVs than direct culture-independent sequencing (Fig. 2a). A total of 980 ASVs were recovered by direct sequencing, 83 of which were captured by CBM, which also detected an additional 2799 ASVs (Fig. 2a). Moreover, an average of 49 ASVs were detected in each culture-enriched sample. Simultaneously, taxonomic ranks from phylum to species level were also analyzed and the results revealed that at the genus level, 142 genera were detected by CBM only (102 genera by direct sequencing only) and 63 genera were shared by both CBM and direct sequencing approaches. Similarly, at the phylum level, three specific phyla (*Spirochaetota*, *Fusobacteriota* and *Synergistota*) were detected by CBM, but not found by direct sequencing. In contrast, 9 phyla (*Gemmatimonadota*, *Nitrospirota*, *Planctomycetota*, *Acidobacteriota*, *Chloroflexota*, *Armatimonadota*, *Thermomicrobiota*, *BRC1*, and *Rhodothermota*) were observed only from direct sequencing (Fig. 2b). Overall, these results indicated that the CBM strategy based on the full-length 16S rRNA gene amplicon sequencing could yield a considerable richness of ASVs and taxonomic diversity from desert soils, greatly filling in the vast majority omitted by direct sequencing.

### Bacterial biodiversity and taxonomic profiles of original soils and associated cultures

**Fig. 3:**
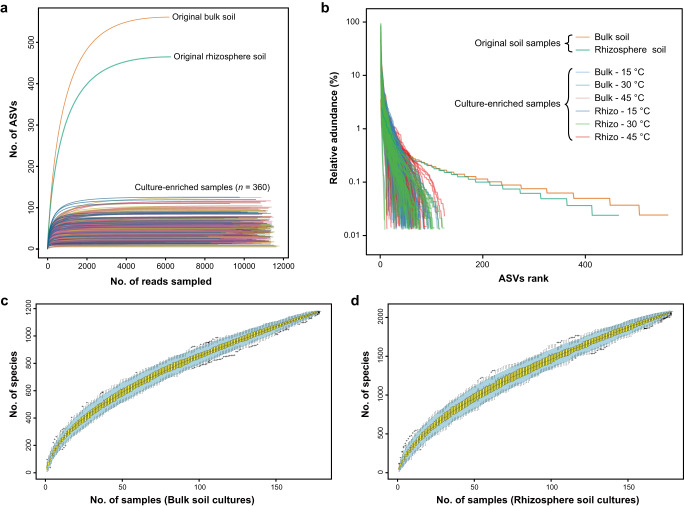
Rarefaction, rank abundance and species accumulation curves of all the samples. **a** Rarefaction curves. **b** Rank abundance curves. **c**, **d** Species accumulation curves. The species accumulation curves were along with 95% confidence intervals based on 1000 random samplings for both the cultures of bulk and rhizosphere soils.

**Fig. 4:**
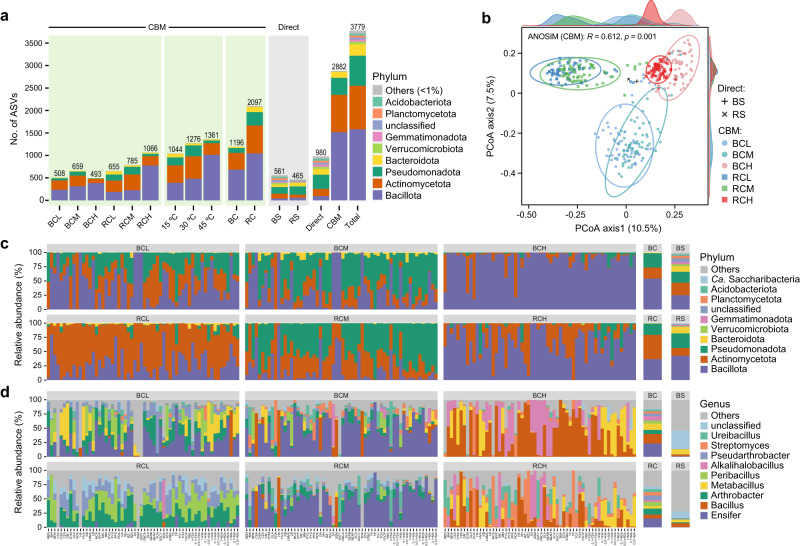
Bacterial diversity and taxonomic composition of original soil samples and associated cultures. **a** The number of ASVs (with phylum level classification and coloring) detected by direct sequencing of original soil samples and by CBM approach of cultures under different subgroups. **b** PCoA plots based on binary Jaccard distances shows the bacterial community similarity among samples. **c**, **d** The barcharts depict the taxonomic assignment of all ASVs annotated at the phylum and genus levels, respectively; The top 10 taxa with the highest relative abundances and “Others” are presented. BC bulk soil cultures, RC rhizosphere cultures, BS original bulk soil, RS original rhizosphere soil; other abbreviations are the same as those illustrated in Fig. 1.

The rarefaction curves all approached saturation, indicating that the microbial biodiversity for each sample was adequately covered, both in the original soils and the culture-enriched samples (Fig. 3a). The rank abundance curves suggested that the richness and evenness of original soil samples were much higher than any of their associated cultures (Fig. 3b). Furthermore, we showed that the bacterial communities between the two original soils and between three incubation temperatures differed significantly (Supplementary Fig. 1). Alpha diversity indices also showed that the diversity of each culture-enriched sample was significantly lower than the corresponding original soil (Supplementary Table 1). Additionally, the total ASV numbers under different subgroups were counted and assigned at the phylum level (Fig. 4a).

Different culture conditions played an important role in capturing the diversity of bacterial communities. Figure 4b–d showed the taxonomic distribution and β-diversity relationships reflected by direct and culture-enriched full-length 16S amplicon sequencing under different culture media and temperature conditions. The results revealed that the bacterial taxa were encouraged with the use of both selective culture media and different incubation temperatures (see Supplementary Dataset 1 for the images of all the plates generated by culturomics, *n* = 1800). Moreover, permutational multivariate analysis of variance (PERMANOVA) results showed that the cultured communities were significantly influenced (*p* = 0.001) by the temperature, medium, and soil, which could explain the total variance of 20.5%, 19.8%, and 6.3%, respectively (Supplementary Table 2). Linear discriminant analysis effect size (LEfSe) analysis was performed for further clarification of bacterial taxa under different culture conditions, and the results revealed the significant effect of different treatments on the composition and diversity of different taxa (Supplementary Fig. 2). These results showed that the bacterial taxa cultured from desert soils can be highly influenced by different temperature, media and soil characteristics conditions, and the metabolic functions of these unexplored taxa need further recognition.

**Fig. 5:**
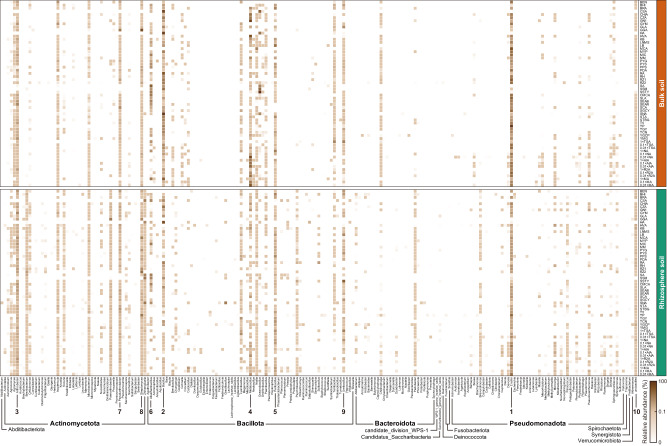
The average relative abundance of each genus obtained from the cultures of bulk (orange) and rhizosphere (green) soils across 60 media. The horizontal coordinates indicate the 204 genera (including the “unclassified”) of 12 phyla obtained via culturomics (in alphabetical order). The top 10 genera with the highest relative abundance (*Ensifer*, *Bacillus*, *Arthrobacter*, *Metabacillus*, *Peribacillus*, *Alkalihalobacillus*, *Pseudarthrobacter*, *Streptomyces*, *Ureibacillus* and unclassified) are numerically labeled (in order of rank).

At the phylum level, two original soil samples were dominated by *Bacillota*, *Actinomycetota*, *Pseudomonadota*, and *Bacteroidota*, which were also absolutely predominated in the entire culture-enriched samples (Fig. 4c). At the genus level, we found that the six subgroups (soil-temperature) of culture-enriched samples had visual differences, and the abundant genera detected by direct sequencing were remarkably different from that of CBM (Fig. 4d). Similarly, Fig. 5 showed the average relative abundances of 204 genera (including the “unclassified”) obtained by CBM. It was found that the use of two soils and different media drove a diverse collection of culturable bacterial genera. In addition, the relative abundances of the top 35 most abundant ASVs (>0.5%) in culture-enriched samples are shown in Supplementary Figs. 3 and 4. The most abundant member, ASV1, classified as *Ensifer meliloti* (synonym: *Sinorhizobium meliloti*), could be recovered by almost all the media (except for CSA and SA). Four ASVs (ASV5, ASV22, ASV23, and ASV34) belonging to the species *Alkalihalobacillus clausii* showed very similar distribution characteristics under different culture conditions (Supplementary Fig. 3). Overall, the combination of systematic culturomics and full-length 16S rRNA gene amplicon sequencing greatly improved the taxonomic diversity and resolution of desert soil microbiota.

### Strain-level analysis reveals a large number of cultivable novel bacterial taxa in desert soils

**Fig. 6:**
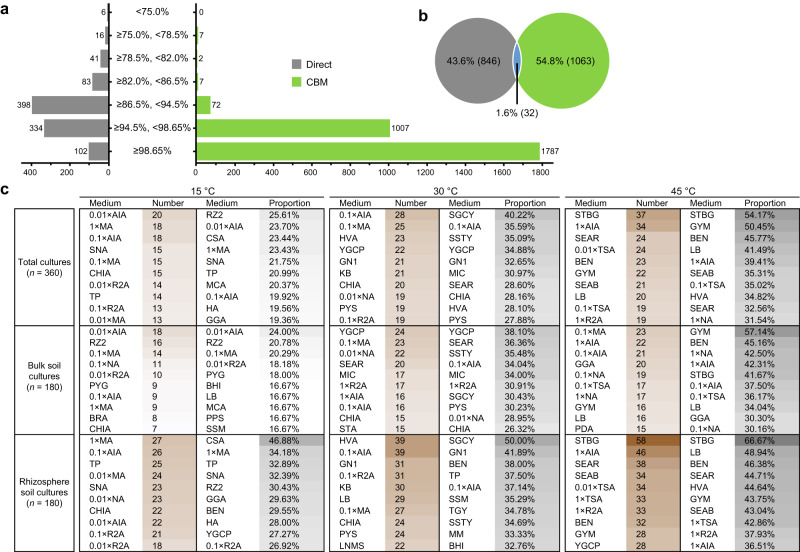
Profiles of potentially novel taxa and ranking of medium isolation efficiency. **a** The ASV number of potentially novel taxa detected by direct sequencing (gray) and CBM (green), based on the classification thresholds at different taxonomic levels (phylum: 75.0%; class: 78.5%; order: 82.0%; family: 86.5%; genus: 94.5%; species: 98.65%). **b** Venn diagram shows the ASV number of unique and shared potentially novel taxa detected by direct sequencing and CBM. **c** The top 10 media with the highest number or proportion (%) of potentially novel taxa.

The novelty of all 3779 ASVs was analyzed through a high-throughput BLAST search against the NCBI 16S rRNA database. Subsequently, a total of 1941 (51.4%) ASVs detected from all samples (*n* = 362) were identified as potentially novel taxa (PNT), leaving 1838 (48.6%) as known species (KS). As shown in Fig. 6a, a huge number of PNT (1095) were identified by CBM, most of which were attributed to potentially novel species (1007), far exceeded than that detected by direct sequencing. However, only 88 ASVs assigned to potentially novel genera or other higher taxonomic ranks were detected in all culture-enriched samples. Meanwhile, the PNT detected by direct sequencing was dominated by potentially novel genera with 398 ASVs, accounting for 40.6% of the total ASVs of original soil samples. Notably, 6 ASVs with less than 75.0% 16S rRNA gene sequence identities were also inferred as potentially novel phyla by direct sequencing (Fig. 6a). Furthermore, it was found that only 32 (1.6%) PNT were shared by both direct sequencing and CBM (Fig. 6b). Additionally, the effects of media on the cultivation of PNT were also analyzed (Supplementary Fig. 5 and Supplementary Table 3), and the top 10 media with the highest number or proportion of PNT were listed in Fig. 6c. These results revealed that the desert soils are a great treasure trove of novel bacterial taxa, while the use of CBM allowed the access to large amounts of potentially novel species that could not be captured by direct sequencing.

### CBM allows for the post hoc recovery of microbes of interest

**Fig. 7:**
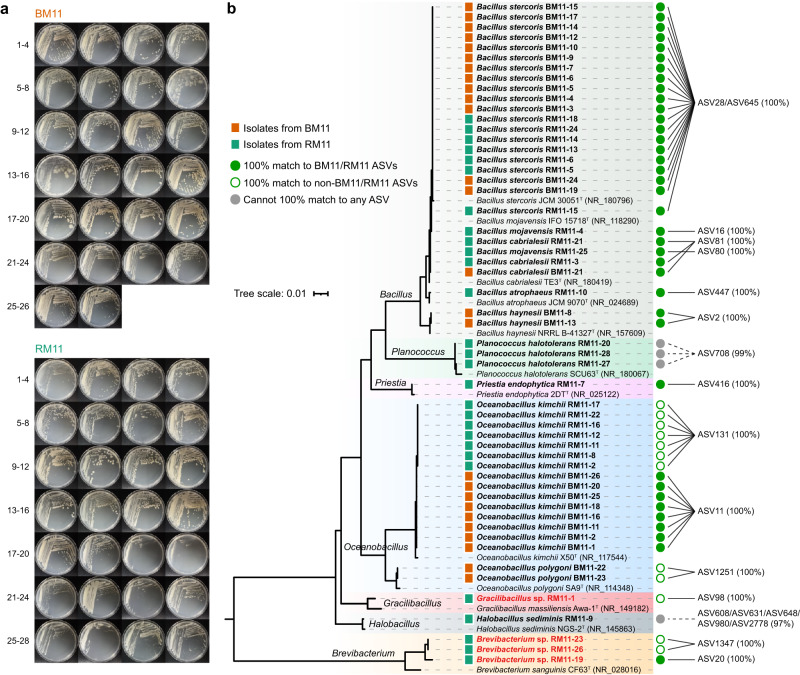
Phylogenetic tree and culture characteristics of the strains recovered. **a** The 26 strains from BM11 and 28 strains from RM11 harvested from M11 (HA) agar were imaged after culturing on HA agar for 5 days. **b** A maximum-likelihood tree based on the 16S rRNA gene sequences of the 54 recovered strains and their closely related type species. *Escherichia coli* ATCC 11775^T^ (X80725) was used as an outgroup. Blocks indicate the isolation source of the strains, BM11 (orange) or RM11 (green). Green solid circles indicate these strains that can 100% match to the ASVs of the corresponding original culture sample; green hollow circle indicate these strains that can 100% match to the ASVs of non-BM11 and non-RM11 culture(s); and gray solid circles indicate these strains that cannot 100% match to any ASVs generated in this study. The strains with red and bold names are classified as potentially novel species, while the others belong to known species. The closest ASVs detected via local blast were listed with the relevant 16S rRNA gene identities in brackets.

Based on the morphological de-duplication, 26 and 28 strains were isolated from the frozen bacterial stocks of BM11 and RM11, respectively (Fig. 7a). These strains were classified into 7 genera (*Bacillus*, *Planococcus*, *Priestia*, *Oceanobacillus*, *Gracilibacillus*, *Halobacillus*, and *Brevibacterium*). As shown in Fig. 7b, we found 50 strains (92.6% of the total) had 100% 16S rRNA identities to the ASVs generated in this study. Among these strains, 38 strains matched 100% BM11/RM11 ASVs, 12 strains matched 100% non-BM11/RM11 ASVs, and the remaining 4 strains couldn’t match 100% any ASVs. In addition, it was found that four strains belonging to the phylum *Bacillota* (*Gracilibacillus* sp. RM11-1) and *Actinomycetota* (*Brevibacterium* sp. RM11-19, *Brevibacterium* sp. RM11-23 and *Brevibacterium* sp. RM11-26) were classified as potentially novel species with 16S rRNA identities less than 98.65%^39^. Notably, the candidate *Brevibacterium* sp. RM11-19 which can 100% match to ASV20, had an extremely low relative abundance (0.03%) with only 3 CCSs detected in RM11. The taxonomic assignments of all isolates are listed in Supplementary Table 4. These evidences indicated that the targeted isolation focuses on the microorganisms of interest might be achieved via the CBM and post hoc recovery approaches.

### CBM greatly improves the harvestability, assembly quality of MAGs and the community functional resolution

**Fig. 8:**
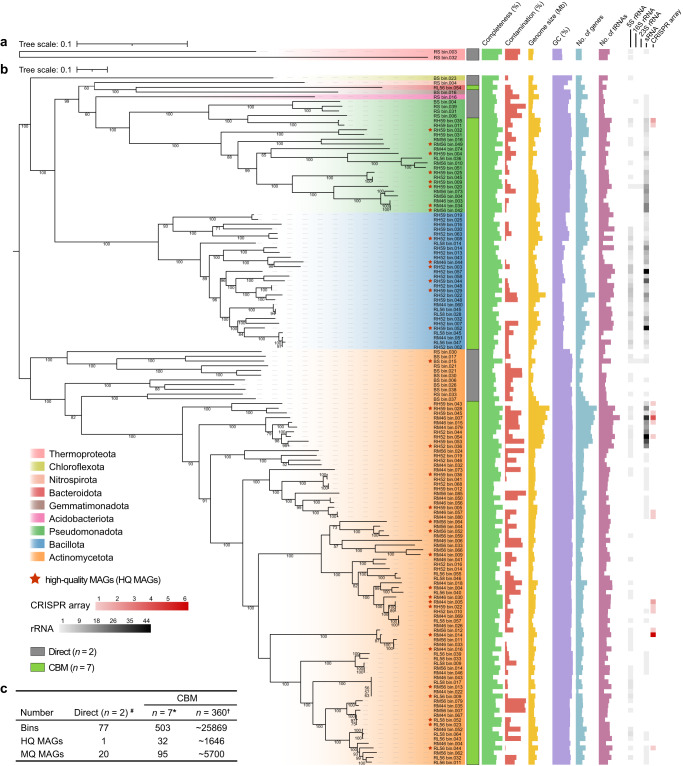
The phylogenomic trees and summaries of 148 high- or medium-quality MAGs. The phylogenomic trees of two archaeal (**a**) and 146 bacterial (**b**) MAGs. Red pentagrams indicate HQ MAGs (completeness >90% and contamination <5%). The taxonomic classifications of all MAGs at the phylum level were displayed by different background colors. Completeness, contamination, genome size, GC, number of predicted genes, tRNAs, 5/16/23s rRNA, sRNA as well as CRISPR arrays for each MAG were shown by bar charts or heatmaps (see Supplementary Table 6 for the detailed values). **c** The assembly results for metagenomic samples of original soils (direct, *n* = 2) and cultures (CBM, *n* = 7). “^#^” indicates the samples (Bulk and rhizosphere soils) for direct metagenomic sequencing; “*” indicates the culture samples (RL56, RL58, RM44, RM46, RM56, RH52, and RH59) for culture-enriched metagenomic sequencing; “^†^” shows the predicted values for all the culture-enriched samples based on the assembly results of seven selected cultures.

The assembly of shotgun sequencing data (300.14 Gbp) generated 790,432 contigs with length ≥1500 bp from two original soils and 7 cultures (Supplementary Table 5). In total, 32,515 contigs of ≥10 Kbp and 708 contigs of ≥100 Kbp were obtained, yielding a total of 580 bins through the binning process. Among these bins, 33 high-quality MAGs (HQ MAGs) and 115 medium-quality MAGs (MQ MAGs) were achieved (Supplementary Table 6). In total, 148 MAGs were assigned to 2 archaeal and 146 bacterial reference taxa (Fig. 8a, b) in the Genome Taxonomy Database (GTDB)^40^. Remarkably, 32 HQ MAGs were derived from the cultures of rhizosphere soil, while no HQ MAGs were assembled by direct shotgun-sequencing of original rhizosphere soil (Fig. 8a, b and Supplementary Table 6). Notably, two HQ MAGs (RM44_bin.034 and RM56_bin.042) were assigned to the species *Ensifer meliloti*, which was the most abundant species in the PacBio SMRT sequencing data. In addition, we found that the number of contigs with length ≥100 Kbp was significantly and positively correlated with the number of HQ MAGs obtained (*R* = 0.95, *p* = 9e^−5^) (Supplementary Fig. 6).

**Fig. 9:**
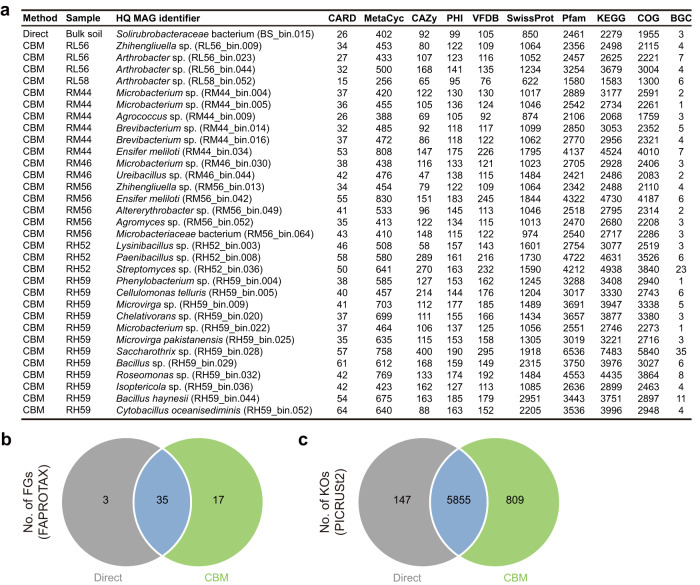
Functional diversity detected by CBM and direct metagenomic sequencing. **a** Taxonomic and functional profiles of all the HQ MAGs (*n* = 33). The numbers of predicted genes annotated to CARD, MetaCyc, CAZy, PHI, VFDB, SwissProt, Pfam, KEGG, COG and BGC are listed for each HQ MAG. **b**, **c** Venn diagrams of common/unique functional groups and KEGG orthology predicted based on the direct sequencing and CBM. The data used for FAPROTAX/PICRUSt2 prediction is derived from the full-length 16S rRNA gene sequencing of original soils and cultures. FGs functional groups, KOs KEGG orthology.

The increased taxonomic diversity obtained via CBM directly translates into increased functional diversity in the community. The functions of encoded proteins for each HQ MAG were derived by the exploration of databases such as KEGG, COG, antiSMASH, etc. As shown in Fig. 9a, the culture-enriched shotgun sequencing clearly provided a greater number of functional identifications. For example, RH52_bin.036 (*Streptomyces* sp.) and RH59_bin.028 (*Saccharothrix* sp.) which were only binned from cultures contained conspicuously large numbers of BGCs (Fig. 9a and Supplementary Table 7). It is noteworthy that these landscapes of MAG-based functional diversity derived from only 7 of 360 cultures. The other great advantage of CBM is that the MAGs obtained under different culture conditions have a quite wide range of taxonomic differences (Fig. 8), which could greatly complement the gaps in functional annotation not provided by direct shotgun sequencing of original soils (Fig. 9a).

Correspondingly, the functional metabolic potential of microbial community was predicted using Functional Annotation of Prokaryotic Taxa (FAPROTAX) and Phylogenetic Investigation of Communities by Reconstruction of Unobserved States 2 (PICRUSt2) based on the full-length 16S rRNA gene sequencing data. According to the FAPROTAX results, 1496 out of 3779 ASVs (39.6%) were assigned to at least one function of 55 functional groups (FGs). Among these, 38 (69.1%) FGs contributed by 207 ASVs were obtained by direct sequencing, and 52 (94.5%) FGs from 1326 ASVs appeared in the data of CBM. Seventeen FGs were culture-specific and only 3 FGs were original soil-specific (Fig. 9b). For both culture-enriched and original soil samples, chemoheterotrophy, aerobic chemoheterotrophy and nitrate reduction were the most three abundant FGs (Supplementary Fig. 7a, b). Based on the PICRUSt2 results, 6811 predicted KEGG ortholog (KOs) were recorded. Among which, 5855 KOs were shared by the original soils and culture-enriched samples, 147 were original soil-specific, and 809 were culture-specific (Fig. 9c). The functions related to metabolism were the most abundant in all samples, and the abundance profiles of the KEGG pathway (L2) are shown in Supplementary Fig. 7c, d. Actually, there was a highly significant positive correlation between the number of FGs and KOs (*R* = 0.71, *p* < 2.2e^−16^) (Supplementary Fig. 8).

## Discussion

Deserts are one of the most important ecosystems on Earth, yet they have not been concerned by microbiologists due to their extreme specificity and lifelessness. With further research and accumulation of sequencing data, the huge microbial biodiversity enclosed in unexplored extreme habitats has been gradually revealed^41^, including deserts^15–17,42,43^. However, due to the high complexity and low biomass of bacterial communities in desert soils, it is often difficult to capture typically rare but important microbes in environmental reservoirs through direct metagenomic sequencing^4,42^. Additionally, the standard culturomic approach based on high-throughput MALDI-TOF identification of colonies cannot fully capture information on all cultures^36^. In this study, we conducted a systematic culturomic approach and proposed the use of culturomics-based metagenomics (CBM) to both magnify signals of specific taxa selectively enriched by different culture conditions and considerably simplify microbial diversity of metagenomic samples (Fig. 1), as recently achieved when applied to the gut^38,44^, lung^37^, wastewater^45^, and sediment^46^ samples. We demonstrate that the CBM strategy which integrates large-scale culturomics, full-length 16S rRNA gene amplicon, and shotgun metagenomic sequencing can greatly improve the taxonomic and functional resolution, shedding light on the undiscovered novel microbial resources in desert soils.

Using the CBM strategy, we recover the majority of ASVs and microbial biodiversity that were missed in direct culture-independent sequencing (Fig. 2), which is consistent with previous studies using culture-enriched metagenomics in various environments^37,47–49^. These results Indicated that CBM can be a powerful booster in the surveys of microbial biodiversity, because it allows the exploration of rare biosphere in low-biomass samples, which are typically not detectable or difficultly detectable by direct sequencing. But this is not to detract from direct sequencing approaches; the phylogenetic coverage of culture-independent metagenomics will always be better than that from culturomics^50,51^. Combining culture-independent techniques with microbial culturomics is a valuable complementary approach that enables us to better understand the diversity and unculturability of bacterial communities. Additionally, we should be aware of the importance of a comprehensive and systematic culturomic approach is very important in support of CBM, since culture-enriched samples obtained from a single or a few culture conditions are always not sufficiently representative to reflect most of the characteristics of a culturable community^52,53^. Herein, although we used relatively comprehensive culture conditions (*n* = 180) for each soil sample, the species accumulation curves were still unsaturated (Fig. 3c, d), therefore, adding new culture conditions would lead to the detection of an extra microbial diversity.

Culturomics is an efficient strategy for isolating novel and understudied microorganisms^51^, which are excellent materials for mining environmental microbial resources. We showed that the desert soil is absolutely a great treasure trove of uncharted novel bacterial resources, according to the strain-level analysis results of full-length 16S sequences data generated by both direct sequencing and CBM approaches. Encouragingly, more than half of the ASVs (1941, 51.4%) can be classified into potentially novel bacterial taxa overall, with 1095 of them being cultivable. However, despite the use of comprehensively multiple conditions, culturing novel bacterial lineages at the high taxonomic levels still appeared to be very difficult, as evidenced by the large number of novel high-level taxonomic units detected by direct sequencing (Fig. 6a). This may be due to the majority of these huge novel and high-level bacterial lineages have very low nutrient requirements, while oligotrophic taxa are typically less amenable to in vitro cultivation-based investigations^53,54^.

Currently, the availability of microorganisms in pure culture remains the most important cornerstone in microbial physiology for in-depth studying the roles of genes, proteins and metabolic pathways^55,56^. The recovery validation results showed that the microorganisms of interest (e.g., novel or specific taxa) in cultures might be recovered after culture-enriched sequencing, as shown in Fig. 7. These results further illustrate that sequencing studies can prompt targeted culturomics to culture microorganisms of interest, emphasizing the complementarity between culturomics and culture-independent studies^51^. In fact, the rapidly expanding culture-independent metagenomic studies have generated so many correlative investigations and untested hypotheses, therefore, it is critical to isolate key strains from the environmental microbiota^35,53,57,58^, and CBM allows for such isolation attempts.

In this study, culture-enriched metagenomics also performed better than direct metagenomic sequencing in high-quality genome reconstruction, as reflected in the availability of MAGs at both the diversity, quantity and quality levels (Fig. 8 and Supplementary Table 6). In addition, total 32 HQ MAGs were obtained from only 1.94% of the total cultures using culture-enriched metagenomics. In contrast, direct metagenomic sequencing was far less favorable for achieving the above goals. Moreover, no HQ MAGs and only 1 HQ MAGs were recovered from rhizosphere and bulk soil metagenomes respectively, even at much high sequencing depths of over 30 Gb per sample. However, it is worth noting that compared to culture-enriched metagenomics, direct shotgun sequencing still has significant advantages in obtaining MQ MAGs of some uncultured microbes with deep branches, including the archaeal MAGs (e.g., RS_bin.003 and RS_bin.032) (Fig. 8a, b and Supplementary Table 6). Based on the results of existing culture-enriched metagenomics, we estimated that the whole cultures (*n* = 360) might potentially yield about 25,869 bins, 1646 HQ MAGs, and 5700 MQ MAGs (Fig. 8c). In addition to improve taxonomic classification, access to more comprehensive recovery of MAGs (as well as ASVs) via culture-enriched metagenomic sequencing fundamentally alters the resolution of functional analysis, which has been demonstrated in this study (Fig. 9), as well as previous microbiome studies^37,38,45,46^. An extended collection of HQ MAGs by culture-enriched metagenomic sequencing and genome-resolved metagenomic analysis is essential for the in-depth functional profiling of desert microorganisms. With these high-quality bacterial genomes, we can better study the metabolic functions and related mechanisms of microbial dark matter, and better understand the gene repertoire of individual microorganisms in desert environments^11,59^.

However, there are some limitations to this study. The application of selective culture conditions was allowed to proliferate the low-abundance microorganisms and to enhance the recovery of bacterial diversity from the studied samples. However, it was recorded that the results parallelly supports the abundance of specific taxa of 16S amplicon and shotgun sequencing compared to the sequencing from the original samples, which may cause the underestimation or ignorance of the uncultured taxa. Thus, the CBM approach may not reflect the true relative abundance of microbial composition and functional pathways in the respective environment. Also, only several representative cultures (1.94%) were selected as examples for shotgun sequencing, which makes it impossible to accurately determine how many available MAGs could be recovered from all cultures. In addition, the culturomic strategy used in this study was based on the traditional agar plates, in which the sample size and culture conditions may still be insufficient to represent enough sample heterogeneity and biodiversity. Promisingly, the combination of CBM strategy with some advanced cultivation techniques (e.g., microfluidics, dilution-to-extinction, and single-cell sorting, etc.) and long-read shotgun sequencing may overcome the above limitations, and achieve high-throughput diversity exploration and high-quality genome reconstruction in a faster, cheaper, more convenient and more efficient way. This will make the culturomics-based metagenomics proposed here more powerful tool in desert microbial dark matter mining.

We conclude that the integration of the high-resolution CBM strategy with direct metagenomic sequencing enables in-depth profiling of the microbial dark matter in desert soils. The CBM strategy, an approach that integrates culturomics and metagenomics (full-length 16S amplicon and shotgun sequencing) can greatly improve the taxonomic and functional resolution of desert soil microbiome, and importantly it allows the post hoc recovery of microbes of interest based on the metagenomics-guided isolation. Benefiting from species-level analyses, we have also revealed the huge underexplored potential of novel bacterial resources in desert soils. Furthermore, the results of culturomics under multiple conditions provide an important reference for the isolation of certain special or novel bacterial taxa in desert soils. With these data in hand, we can better understand the composition and distribution, microbe-microbe interactions, environmental adaptation mechanism and gene repertoire of desert microbiota. Culturomics-based metagenomics, as exemplified strategy here for desert soils, provides a new perspective for deeper understanding and mining microbial dark matter in microbiome samples, especially those from extreme habitats.

## Methods

### Sampling and pre-selection of experimental samples

Seven soil samples (~500 g for each) were collected on 21st June 2021 from two different sites (5–20 cm depth) in the Gurbantunggut Desert, Xinjiang, northwestern China (Site 1: 44°53′9″N, 86°18′21″E; Site 2: 45°15′59″N; 85°2′21″E; Supplementary Fig. 9). One bulk soil was collected from each sampling site. The rhizosphere soils of *Haloxylon ammodendron* and *Calligonum leucocladum* (*n* = 2) were sampled from Site 1, while the rhizosphere soils of *Haloxylon ammodendron*, *Tamarix chinensis* and *Populus euphratica* (*n* = 3) were taken from Site 2 (Supplementary Fig. 10). The samples for DNA extraction were immediately placed on dry ice and transported to the laboratory and stored at −80 °C until further processing, and the samples for cultivation were placed on ice and kept at 4 °C until the isolation procedure was performed.

Plant and soil types are the two main drivers of the soil microbial community^60^. Thus, prior to implementing large-scale cultivation (culturomics), a pre-experiment was carried out for the pilot screening of soil samples. An amount of 10.0 g of each soil was suspended in sterile phosphate buffer saline with glass beads (3 mm diameter) to make the final volume 100 ml and kept in a rotary shaker at 30 °C, 180 rpm for 1 h. The suspensions were 10-fold serially diluted, and aliquots of 100 μl of dilutions 10^−2^–10^−5^ were spread onto Reasoner’s 2A agar (R2A) and tryptic soy agar (TSA). After 2.5–5 days of incubation at 30 °C, all the plates were imaged and compared comprehensively (Supplementary Fig. 10). After that, the rhizosphere soil of *Calligonum leucocladum* was selected on the basis of visually highest colony forming unit and morphological diversity of culturable bacteria on agar plates as the subsequent experimental rhizosphere sample, and the associated bulk soil was also used.

### Culturing of soil samples via culturomics

Cultivation for each soil was conducted with a total of 180 different culture conditions, including 60 different agar media and 3 different temperatures. The media components and preparation details are included in Supplementary Table 8. Wherein, five medium categories (TSA, NA, AIA, R2A and MA) were employed and diluted 0, 10 and 100 times, yielding a total of 15 media (M46–M60). Trace salt and B-vitamins were filtered and supplemented as described previously^61,62^. Nalidixic acid (final concentration: 25 mg/l), cycloheximide (50 mg/l) and nystatin (50 mg/l) were supplied to inhibit the growth of fast-growing bacteria and fungi^42^. Catalase (15 U/l) was used to remove peroxides produced during autoclaving^63,64^.

Two experimental soils were pretreated in the same way as the pre-experiment mentioned above. A 100 μl from 10^−1^–10^−5^ dilutions were spread onto each test agar medium, and cultivated at 15, 30 and 45 °C for 3–15 days, generating a total of 1800 plates (360 treatments × 5 dilutions). During the long incubation process, double-layer sterile fresh-keeping bags were used to seal the agar plates to prevent the moisture loss and the external disturbances, and the Petri dishes were placed inverted to prevent the accumulation of water condensation and to lessen the contamination risks from airborne particles landing on the agar. The incubation time of each treatment (5 plates) mainly depended on the overall biomass and diversity of growing colonies, which would be harvested until the growth essentially stops increasing (Supplementary Table 9 and Supplementary Dataset 1). After that, all the colonies on each plate were harvested by adding 5 ml 1× phosphate buffer saline and scrapping off the surface with a sterile cell scraper. In addition, it is worth to be noted that the collected cultures from different dilutions (10^−1^–10^−5^) of each medium/soil/temperature pairing were combined. For each harvested suspension (about 3 ml), 1 ml was added to 1 ml of 50% sterile glycerol and stored at –80 °C, and the remaining 2 ml was used for the DNA extraction (see Supplementary Text 1 for the details).

### DNA extraction and PacBio SMRT sequencing

The genomic DNA was extracted from original soils and culture-enriched samples using the E.Z.N.A.^®^ Bacterial DNA Kit (OMEGA Bio-tek, Inc., Norcross, GA, USA) according to the manufacturer’s instructions with several modifications (see Supplementary Text 2 for the details). The full-length 16S rRNA gene from genomic DNA was amplified using the primers 27F (5′–AGRGTTTGATYNTGGCTCAG–3′) and 1492R (5′-TASGGHTACCTTGTTASGACTT–3′) tailed with sample-specific PacBio barcode sequences. PCR amplification was performed using the KOD One^TM^ PCR Master Mix (Toyobo) under the following conditions: denaturation at 95 °C for 5 min, followed by 30 cycles of 95 °C for 30 s, 50 °C for 30 s and 72 °C for 90 s, with a final extension at 72 °C for 7 min; then held at 4 °C. PCR products were quantified by ImageJ based on the electrophoresis results, and then recovered from the gel and purified using MagicPure Size Selection DNA Beads (TransGen Biotech, Beijing, China). Subsequently, the purified PCR products were pooled for multiplex sequencing and library construction with SMRTbell Template Prep Kit v1.0-SPv3. The sequencing of full-length 16S rRNA amplicons was performed on a PacBio Sequel II platform at Biomarker Technologies Co. Ltd. (Beijing, China).

### PacBio SMRT sequencing data processing

The CCSs were generated by correcting the raw subreads on SMRT-Link v8.0 with the following parameters: minPasses ≥5, minPredictedAccuracy ≥0.9. Then lima v1.7.0 was used to distinguish CCSs from different samples through the barcode sequence. Cutadapt v1.9.1 was applied to identify primers. The CCSs between 1200 to 1650 bp were kept after the length-based filtration. UCHIME v8.1 was used to remove the chimeras, allowing the generation of high-quality CCSs. ASVs were generated after denoising with the DADA2^65^ method in QIIME 2 (version 2020.06)^66^. The taxonomic identity for each ASV representative sequence was determined using the Ribosomal Database Project (RDP) Classifier^67^ with the 16S rRNA training set (version 18) requiring a 70% confidence threshold. Because RDP could only provide taxonomic resolution down to genus level, each sequence was also searched against the NCBI 16S rRNA database (version 2022.12.16) using BLASTn^68^. Resulting hits were sorted first by *e*-value, then score, finally identity, and the taxonomy of the highest identity sequence was reported^69^. Nearest-neighbor species with ≥98.65%^39^ identity was selected as a candidate for each sequence, otherwise it was recorded as “unclassified” for the species classification. Finally, we constructed the ASVs’ taxonomy table from domain to species level by combining the annotation results of the RDP and NCBI databases. Besides, the novelty of ASVs at species, genus, family, order, class and phylum levels were roughly matched based on sequence identity thresholds of 98.65%, 94.5%, 86.5%, 82%, 78.5% and 75%, respectively^39,70^. If the 16S rRNA sequence identity between one ASV and its closest species was less than 98.65%^39^, we considered the ASV to represent a potentially novel taxon, otherwise it will be recorded as known species.

### Full-length 16S rRNA sequencing data analysis

The rarefaction, rank abundance, and species accumulation curves were performed by R software (version 4.2.1; https://www.r-project.org/). Alpha diversity indices including observed ASVs, Shannon, Inverse-Simpson, Pielou’s evenness and phylogenetic diversity were computed using “vegan”^71^ and “picante”^72^ packages in R, and the differences between each group was tested by two-sample Student’s *t* test. For soil bacterial beta-diversity, distance matrices were calculated using binary Jaccard distance for the 16S rRNA data, and principal coordinate analysis (PCoA) was employed to visualize the dissimilarity in microbial taxa between samples. Analysis of similarity (ANOSIM) were adopted to test the differences in bacterial communities between and within groups. PERMANOVA on the basis of Bray-Curtis distance matrix was performed to disclose factors (soil, medium and temperature) shaping the culturable desert soil microbiota via the adonis2 function in “vegan” package. Venn diagrams were used to present the shared and unique components among groups using the Biozeron Cloud Platform (http://www.cloud.biomicroclass.com/CloudPlatform). Microbial biomarkers with statistical differences among groups were identified using LEfSe analysis according to the set screening criteria LDA score ≥4.0. FAPROTAX^73^ and PICRUSt2^74^ were used to predict the functions of ASVs.

To construct the ASV-based phylogenetic trees, ASV sequences were first aligned using MUSCLE v3.8.31^75^. Then, IQ-TREE v1.6.12^76^ was employed to implement the maximum-likelihood phylogenetic trees with the automated detection of the best evolutionary model (Total: GTR + F + R7; OSS: TNe + R10; CES: SYM + I + G4) using ModelFinder^77^ with 1000 replicates. The final consensus trees were visualized and annotated with iTOL v6.6 (https://itol.embl.de/)^78^.

### Recovery of isolates from frozen bacterial stocks

To verify the recoverability of frozen bacterial stocks, two samples (BM11 and RM11) cultured on HA (Halophilic Agar) medium with appropriate bacterial diversity were selected as an example for the second round of culturing. Ten-fold serial dilution was performed, and then 100 μl of frozen stocks and dilutions 10^−2^–10^−6^ were spread onto HA medium. After 2 weeks of incubation at 30 °C, the colonies with different morphology were picked and purified on HA medium. The genomic DNA of isolates was extracted by the E.Z.N.A.^®^ Bacterial DNA Kit according to the manufacturer’s instructions as in DNA extraction section. 16S rRNA genes were amplified in all isolates using 27F and 1492R primers as mentioned above and the sequencing was performed via Sanger sequencing. Forward and reverse sequences were aligned and assembled using the SeqMan program (DNAStar, v7.1.0). Almost full-length 16S sequences were identified via the NCBI 16S rRNA sequences (Bacteria and Archaea) database using BLASTn, and were also blasted locally against the ASVs generated from this study. The 16S rRNA gene sequences were aligned using ClustalW^79^, and a maximum-likelihood tree was constructed using the Tamura-Nei model with 1000 bootstrap replicates in MEGA11^80^.

### Shotgun metagenomic sequencing, data processing and analysis

To compare the difference between direct and culture-enriched metagenomic sequencing, the two original soil samples and seven culture-enriched samples (RL56, RL58, RM44, RM46, RM56, RH52 and RH59) were employed, and the source information of these cultures can be found in Supplementary Table 9. Herein, the culture-enriched samples for metagenomics were both from rhizosphere soil cultures at three different temperatures (2–3 for each) with appropriate microbial biodiversity based on the analysis of full-length 16S rRNA sequencing data. The genomic DNA of the nine samples was extracted and quantified as described above. The DNA concentration was measured using the Qubit^TM^ dsDNA HS Assay Kit in the Qubit 3.0 fluorometer (Invitrogen). Degradation and contamination of DNA were checked by 1% gel electrophoresis. About 100 ng of DNA from the selected culture samples and original soil samples were randomly fragmented to ~400 bp by acoustic sonication. The fragmented DNA ends were repaired, polyA-tailed and ligated with adaptors for Illumina sequencing. PCR amplification and DNA purification were performed using the AMPure XP system. Paired-end (PE) library preparations were made with the VAHTS^®^ Universal Plus DNA Library Pren Kit for Illumina. Library sequencing was performed on Illumina NoveSeq 6000 (Illumina Inc., San Diego, CA, USA) platform using NovaSeq 6000 S4 Reagent Kit at Biomarker Technologies Co. Ltd. (Beijing, China) and 2 × 150 bp paired-end reads were generated.

Metagenomic reads were trimmed using Trimmomatic v0.39^81^ with parameters (PE-threads 20 ILLUMINACLIP:TruSeq3-PE.fa:2:40:15 LEADING:3 TRAILING:3 MINLEN:50). The quality-filtered reads were mapped back to the assemblies using Bowtie2. High-quality reads were assembled by SPAdes v3.15.3^82^ using the parameters “-k 21, 33, 55, 77, 99, 127 -meta” with error correction. The quality of the assembled contigs was evaluated using QUAST (v5.2)^83^, and only those with ≥1500 bp were binned by MetaBAT2^84^. Completeness and contamination of MAGs were assessed using CheckM v1.1.3^85^. The MAGs with good quality (completeness ≥50% and contamination <10%) were kept for further analysis. We defined HQ MAGs as those with completeness >90%, contamination <5%, and MQ MAGs as those with completeness ≥50% and contamination <10% referring to the MIMAG standard^86^. All the MAGs were taxonomically classified with Genome Taxonomy Database Toolkit (GTDB-Tk, v1.7.0)^87^ and annotated using the GCM online tool (https://gctype.wdcm.org/)^88^. Secondary metabolism analysis was performed using antiSMASH (version 6.1.1)^89^ with the default parameters. The phylogenomic trees were implemented by IQ-TREE with 1000 replicates under the best evolutionary models (Bacteria: LG + F + R6; Archaea: LG + F + R3) and visualized by iTOL.

### Reporting summary

Further information on research design is available in the Nature Research Reporting Summary linked to this article.

### Supplementary information

Supplementary Information

Reporting Summary

## Data Availability

Raw reads of full-length 16S amplicon sequencing and shotgun sequencing, metagenome-assembled genomes, as well as the 16S sequences of isolates have been deposited into the NCBI Sequence Read Archive (SRA) database and are available under BioProject PRJNA889009. Other data that support the findings of this study are available from the corresponding author upon reasonable request.

## Code availability

The R scripts and relevant data are available on GitHub at https://github.com/lssuiqq/Culturomics-based-Metagenomics.
