# Supplementary material for: Capturing the microbial dark matter in desert soils using culturomics-based metagenomics and high-resolution analysis
Source: NPJ Biofilms Microbiomes. 2023 Sep 22;9:67. doi: 10.1038/s41522-023-00439-8 (PMC10516943; doi:10.1038/s41522-023-00439-8)

Supplementary Information for

Capturing the microbial dark matter in desert soils using culturomics-based metagenomics and high-resolution analysis

Shuai Li[1,2], Wen-Hui Lian[1], Jia-Rui Han[1], Mukhtiar Ali[1], Zhi-Liang Lin[1], Yong-Hong Liu[3], Li Li[3], Dong-Ya Zhang[4], Xian-Zhi Jiang[4], Wen-Jun Li[1,3,]* and Lei Dong[1,]*

[1] State Key Laboratory of Biocontrol, Guangdong Provincial Key Laboratory of Plant Resources and Southern Marine Science and Engineering Guangdong Laboratory (Zhuhai), School of Life Sciences, Sun Yat-sen University, Guangzhou 510275, China

[2] School of Life Science, Jiaying University, Meizhou 514015, China

[3] State Key Laboratory of Desert and Oasis Ecology, Xinjiang Institute of Ecology and Geography, Chinese Academy of Sciences, Urumqi 830011, China

[4] Microbiome Research Center, Moon (Guangzhou) Biotech Ltd., Guangzhou 510700, China

*Corresponding authors:

Wen-Jun Li (liwenjun3@mail.sysu.edu.cn);

Lei Dong (donglei6@mail.sysu.edu.cn)

Contents

Supplementary Text

Supplementary Text 1 | Preparation of culture enrichment samples.

Supplementary Text 2 | DNA extraction processes of culture-enriched samples and original soil samples.

Supplementary Figures

Supplementary Fig. 1 | Comparison of bacterial communities in different original soil samples and culture-enriched samples based on the full-length 16S rRNA gene sequencing.

Supplementary Fig. 2 | Taxonomic cladograms and histograms via LEfSe analysis for culture-enriched samples.

Supplementary Fig. 3 | Heatmap of the top 35 most abundant (>0.5%) bacterial ASVs under each culture condition.

Supplementary Fig. 4 | Boxplots of the top 35 most abundant (>0.5%) bacterial ASVs under different subgroups of culture-enriched samples.

Supplementary Fig. 5 | The number and proportion of potentially novel taxon detected in 360 different cultures.

Supplementary Fig. 6 | Correlations between the number of high-quality MAGs and other characteristics generated by assembly or binning process.

Supplementary Fig. 7 | Distribution of the functions predicted by FAPROTAX and PICRUSt2.

Supplementary Fig. 8 | The correlation between the results predicted by FAPROTAX and PICRUSt2.

Supplementary Fig. 9 | Diagram of sampling sites in the Gurbantunggut Desert.

Supplementary Fig. 10 | The pre-experiment results for the piolet screening of soil samples.

Supplementary Tables

Supplementary Table 1 | Alpha diversity of the two original soil samples and six subgroups of culture-enriched samples.

Supplementary Table 2 | PERMANOVA results of Bray–Curtis dissimilarity between cultures of bulk and rhizosphere soils and across the whole dataset

Supplementary Table 3 | The number and proportion of potentially novel bacterial taxa cultivated on different media.

Supplementary Table 4 | Sanger sequencing results for colonies re-isolated from frozen bacterial stocks of BM11 and RM11.

Supplementary Table 5 | Summary of sequencing, assembly and binning results of nine metagenomic samples.

Supplementary Table 6 | Summary of all the high-quality and medium-quality MAGs obtained in this study.

Supplementary Table 7 | Summary statistics of BGCs for 33 high-quality MAGs predicted by antiSMASH.

Supplementary Table 8 | Media composition used in the present study.

Supplementary Table 9 | Incubation time for each culture-enriched sample.

Supplementary Dataset

Supplementary Dataset 1 | Microbial culture characteristics of bulk and rhizosphere soil samples on 60 media at three temperatures. The left color blocks represent bulk soil (orange) and rhizosphere soil (green-cyan) and the top color blocks represent 15 °C (cyan-blue), 30 °C (green) and 45 °C (red), respectively.

Supplementary Text

Supplementary Text 1 Preparation of culture enrichment samples.

1) Add 5 mL 1× sterile phosphate buffered saline (PBS) buffer solution to the culture plate with a dilution of 10^{-5}, scrape the bacterial biomass on the agar with a sterile cell spatula and mix it well;
2) Transfer the mixed bacterial solution in step 1 to the culture plate of the upper dilution, perform the same operation as in step 1, and so on, and finally obtain about 3–4 mL of mixed bacterial solution;
3) Transfer 2 mL of mixed solution into a sterile 2 mL micro-centrifuge tube, centrifuge at 12,000× g for 10 min, discard the supernatant carefully;
4) Pre-freeze the remaining biomass at –80 °C for 24 h, and then perform freeze-drying treatment for ~48 h;
5) Stored at –80 °C for DNA extraction.

Supplementary Text 2 DNA extraction processes of culture-enriched samples and original soil samples.

The following steps are modified based on the E.Z.N.A. Bacterial DNA Kit (OMEGA). There are minor differences between the DNA extraction of culture enriched samples and original soil samples, mainly in the process of sample pretreatment.

A: For culture-enriched samples:

1) Add ~30 mg glass beads and 470 µL TE Buffer to the sample prepared as described in **Text S1**. Vortex to disperse cells at 4500×g for 3 times (10 s/time);
2) Add 80 µL lysozyme solution (75 mg/mL), vortex to mix well, and incubate at 37 °C for 1 h;
3) Add 55 µL SDS solution (20%, v/v) and 25 µL Proteinase K solution (20 mg/mL), vortex to mix well, and incubate at 56 °C for 50 min, briefly shake the samples every 15 min;
4) Add 5 µL RNase A to samples and invert tube 10 times to mix, incubate at room temperature for 5 minutes;
5) Centrifuge at 13,000× g for 10 min to pellet insoluble debris, and transfer the supernatant to a sterile 2 mL tube leaving behind any insoluble pellet;
6) Add 450 µL Buffer BDL (preheated at 65 °C before use) and briefly vortex to mix, incubate at 65 °C for 12 min;
7) Add 450 µL absolute ethanol (room temperature, 96~100%) and mix thoroughly by vortexing at maximum speed for 15~20 s until the solution is clear and there is no concentration disturbance;
8) Assemble a HiBind DNA Mini column in a 2 mL collection tube (provided by Kit). Transfer the entire sample from Step 7 into the column, including any precipitate that may have formed. Centrifuge at 12,000× g for 1 min to bind DNA. Discard the collection tube and filtrate;
9) Place the column into a second 2 mL tube and wash by adding 550 µL Buffer HB. Centrifuge at 10,000×g for 1 min. Discard flow-through and reuse the collection tube.

10) Place the column into the same collection tube and wash by adding 700 µL DNA Wash Buffer diluted with ethanol. Centrifuge at 10,000× g for 1 min. Discard flow-through and reuse the collection tube.
11) Wash the column with a second 700 µL DNA Wash Buffer and centrifuge as above. Discard flow-through and reuse the collection tube.
12) Using the same 2 mL collection tube, centrifuge HiBind DNA Mini column at maximum speed (≥12,000× g) for 2 min to dry the column.
13) Place the column into a nuclease-free 1.5 mL microfuge tube and add 65 µL of preheated (70 °C) Elution Buffer to HiBind DNA Mini column matrix. Allow columns to incubate for 5 min at 65 °C after addition of Elution Buffer. Centrifuge at 10,000× g for 1 min to elute DNA from the column.
14) Repeat the elution with a second 65 µL Elution Buffer.
15) Subpackage the combined DNA solution into two nuclease-free 1.5 mL microfuge tubes uniformly; one copy for concentration detection and sequencing (~65 µL), the other copy is reserved as backup at –80 °C.

B: For original soil samples:

1) Take 10.0 g of original soil sample in a 50 mL centrifuge tube (pre-added with ~5 g of glass beads added), and add 25 ml 1× PBS Buffer. Vortex at maximum speed for 10 min intermittently to dissociate cells from soil or sand particles and to remove salt and humus;
2) Centrifuge at 12,000×g for 15 min. Discard the supernatant carefully;
3) Add 12 mL TE Buffer to precipitate. vortex to mix well;
4) Add 500 µL lysozyme solution (75 mg/mL), vortex to mix well, and incubate at 37 °C for 1 hour;
5) Add 1.4 mL SDS solution (20%, v/v) and 100 µL Proteinase K solution (20 mg/mL), vortex to mix well, and incubate at 56 °C for 50 min, briefly shake the samples every 15 min;
6) Add 30 µL RNase A to samples and invert tube 10 times to mix, incubate at room temperature for 5 min;
7) Centrifuge at 13,000× g for 15 min to pellet insoluble debris, and transfer the supernatant to a sterile 50 mL tube leaving behind any insoluble pellet;
8) Add 12 mL Buffer BDL (preheated at 65 °C before use) and briefly vortex to mix, incubate at 65 °C for 12 min;
9) Add 12 mL absolute ethanol (room temperature, 96–100%) and mix thoroughly by vortexing at maximum speed for 15~20 s until the solution is clear and there is no concentration disturbance;
10) Assemble a HiBind DNA Mini column in a 2 mL collection tube (provided by Kit). Transfer the entire sample from Step 9 into the column, including any precipitate that may have formed. Centrifuge at 12,000× g for 30 s to bind DNA. Discard the collection tube and filtrate;
11) The subsequent processing steps are consistent with the Steps 9–15 of DNA extraction of culture-enriched samples.

Supplementary Figures

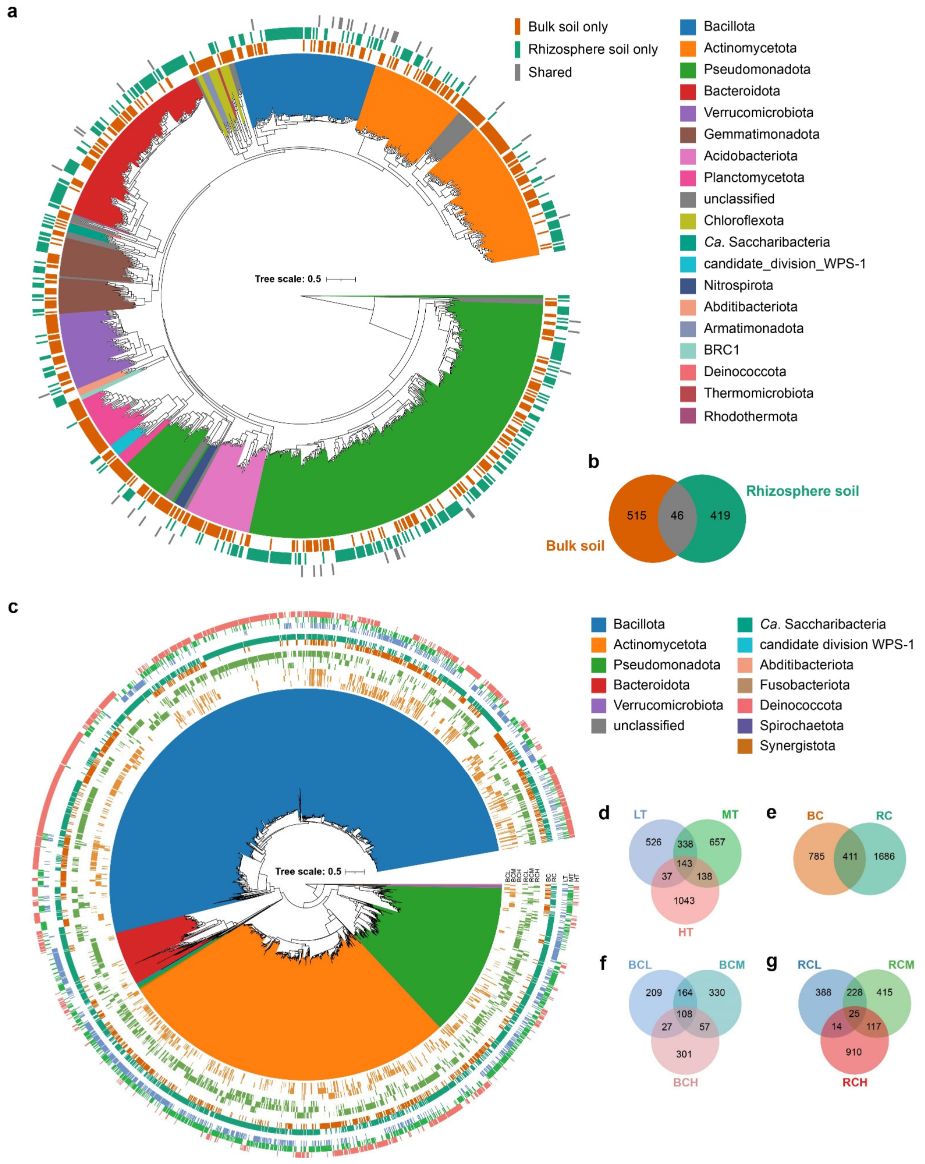

Supplementary Fig. 1 Comparison of bacterial communities in different original soil samples and culture-enriched samples based on the full-length 16S rRNA gene sequencing. a A phylogenetic tree of ASVs detected in both two soils. The colours of rings represented the ASVs detected in the bulk soil only (orange), rhizosphere soil only (green-cyan) and shared by the two soils (grey). **b** Venn diagram of the ASVs detected in two different soils. **c** A phylogenetic tree of ASVs detected in the culture-enriched samples. The outer ring shows the comparison of cultured communities under different subgroups. **d-g** Venn diagrams showing the number of common and unique ASVs in the culture-enriched samples under different groups. Abbreviations: LT (low temperature, 15 °C), MT (moderate temperature, 30 °C), HT (high temperature, 45 °C), BC (bulk soil cultures), RC (rhizosphere cultures), BCL (bulk soil cultures at 15 °C), BCM (bulk soil cultures at 30 °C), BCH (bulk soil cultures at 45 °C), RCL (rhizosphere cultures at 15 °C), RCM (rhizosphere cultures at 30 °C) and RCH (rhizosphere cultures at 45 °C).

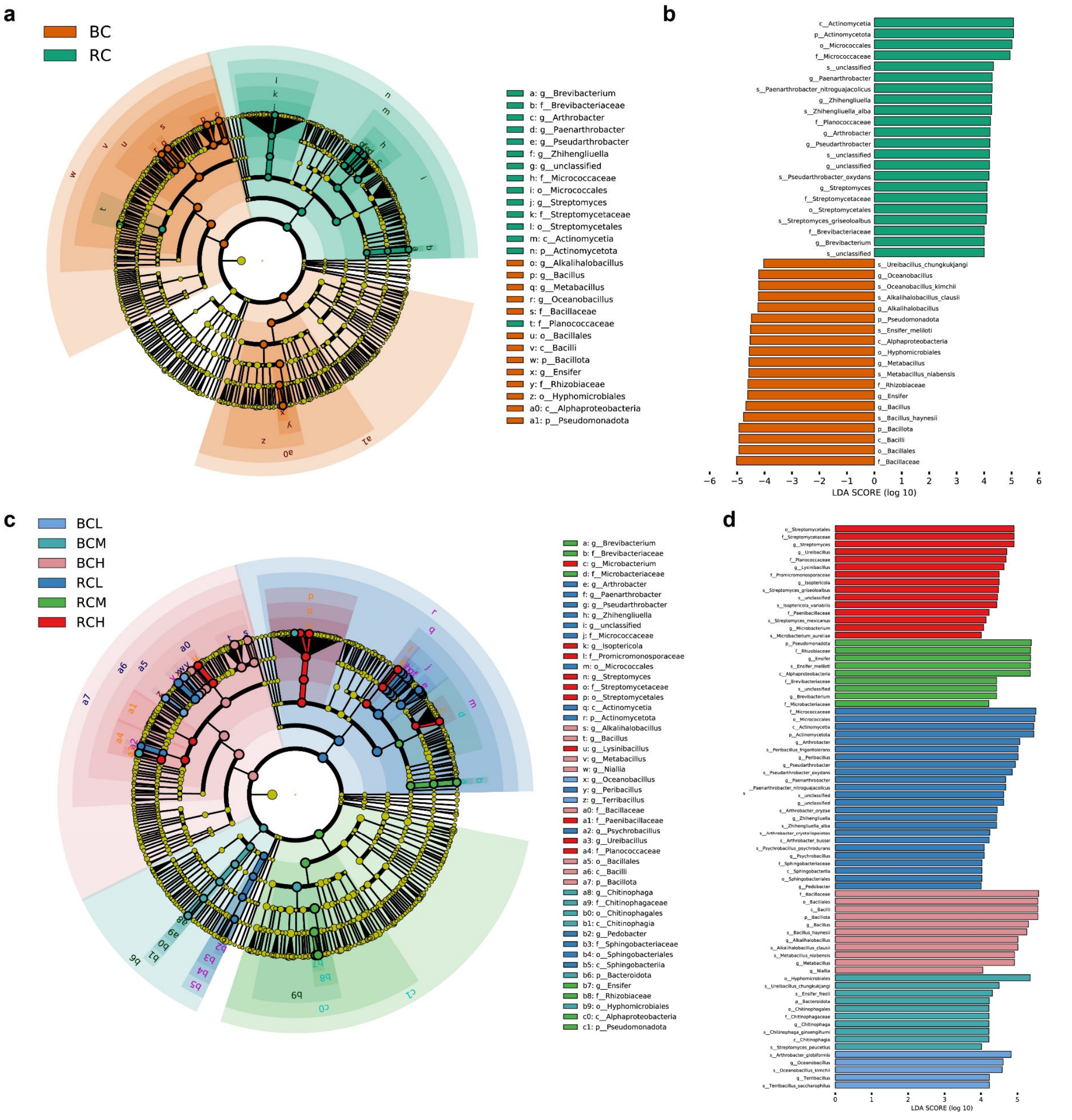

Supplementary Fig. 2 Taxonomic cladograms and histograms via LEfSe analysis for culture-enriched samples. a, c The cladograms of taxa with statistical difference. **b**, **d** The histograms of taxa with statistical difference. The size of each circle in cladograms is proportional to the taxon's abundance. The colours in the histograms indicate enriched taxa in different groups. Abbreviations are the same as those illustrated in Supplementary Fig. 1.

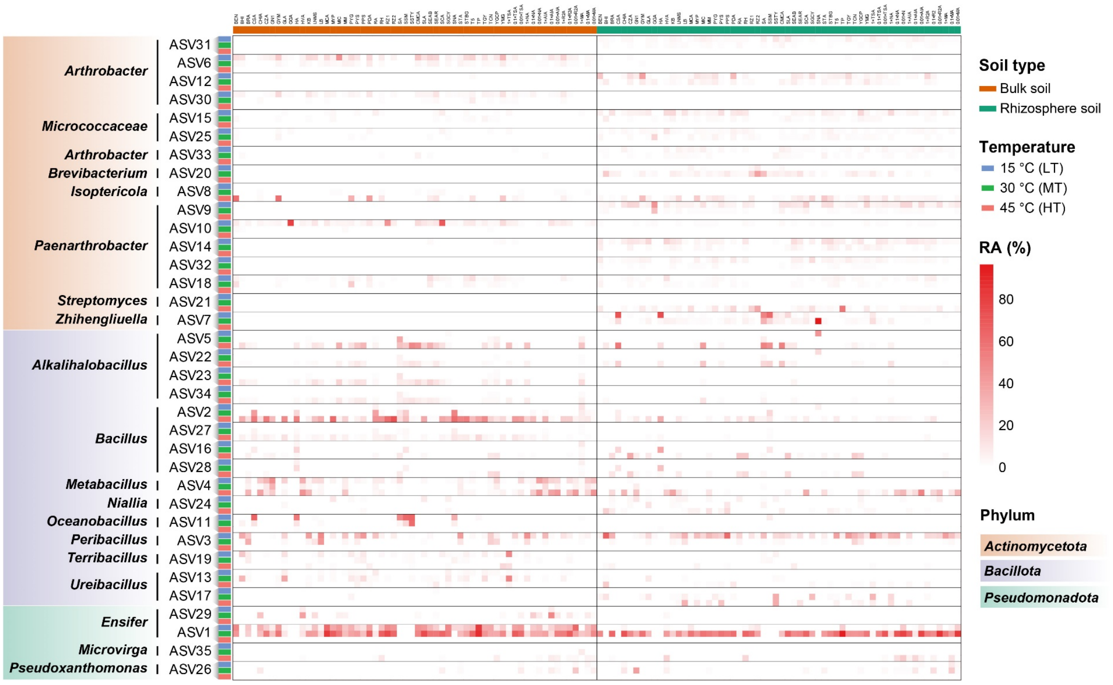

Supplementary Fig. 3 Heatmap of the top 35 most abundant (>0.5%) bacterial ASVs under each culture condition. The 60 columns on the left and right sides indicate the cultures of bulk soil (orange) and rhizosphere soil (green-cyan), respectively. ASVs are arranged in order by their taxonomic phyla (*Actinomycetota* in orange, *Bacillota* in violet and *Pseudomonadota* in green) and genera/families. Each ASV row includes three sub rows differentiated by incubation temperature (blue, 15 °C; green, 30 °C; red, 45 °C). The colour of each individual block in the heat map indicates the relative abundance of ASV under each culture condition. RA, relative abundance.

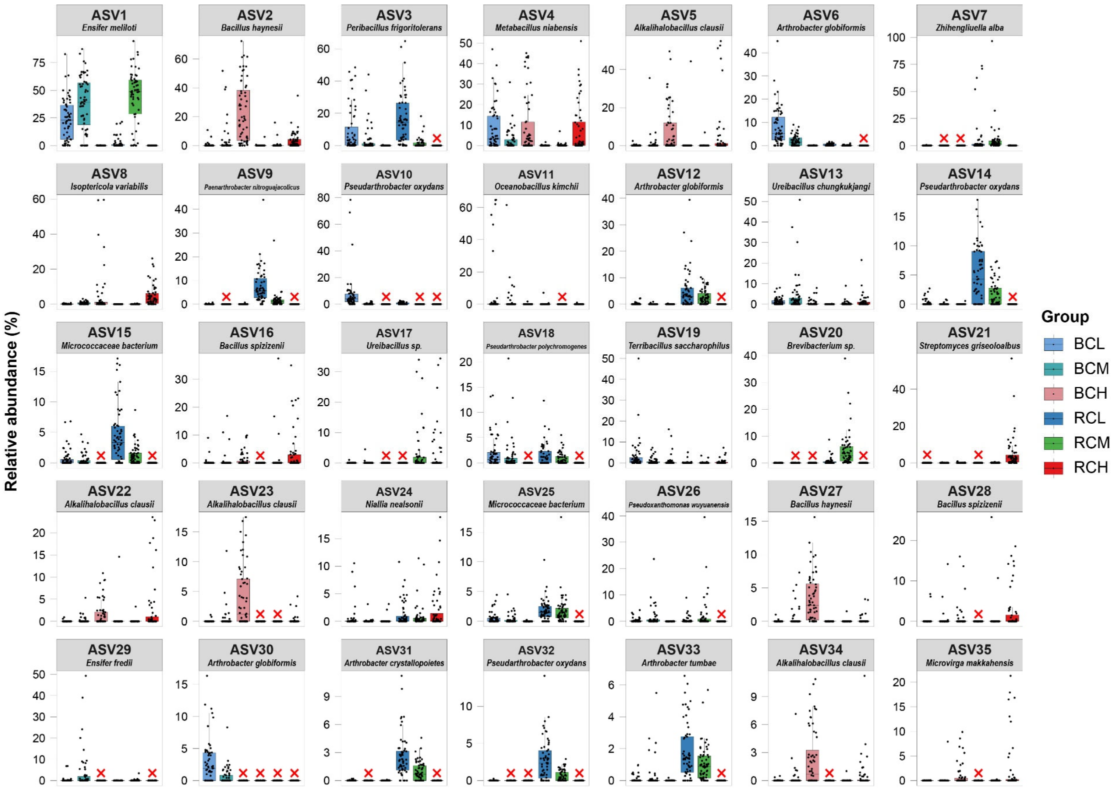

Supplementary Fig. 4 Boxplots of the top 35 most abundant (>0.5%) bacterial ASVs under different subgroups of culture-enriched samples. The corresponding taxonomic status is shown under each ASV identifer. Each scatter indicates an individual sample, which is represented as a separate color block in Supplementary Fig. 3. The red cross symbol indicates that the ASV's relative abundance in all samples of the group is 0, i.e., not detected at all. Abbreviations are the same as those illustrated in Supplementary Fig. 1.

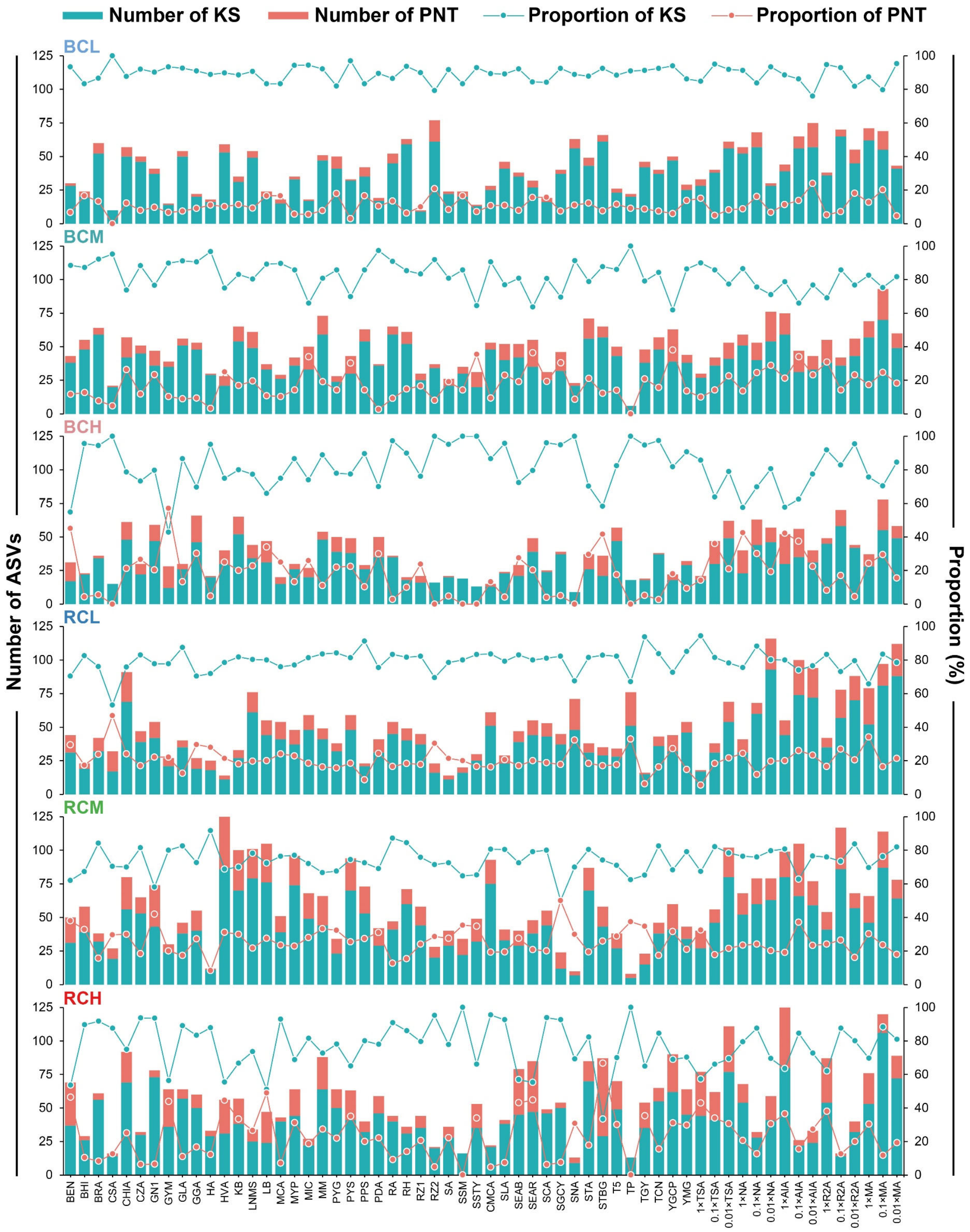

Supplementary Fig. 5 The number and proportion of potentially novel taxon detected in 360 different cultures. KS, know species; PNT, potentially novel taxon. The number (left y-axis) is shown as bar and the proportion (right y-axis) is shown as line. X-axis showing the abbreviation of 60 media used in this study. The six subgraphs from top to bottom indicate groups BCL, BCM, BCH, RCL, RCM and RCH, respectively. Abbreviations are the same as those illustrated in Supplementary Fig. 1.

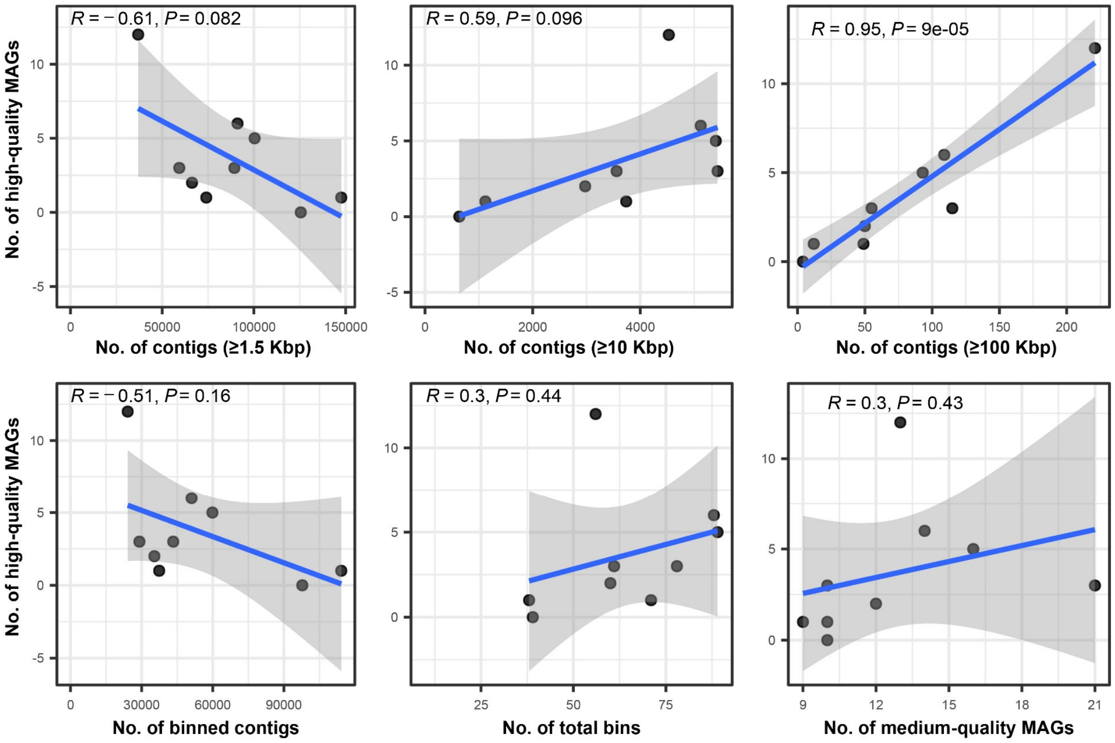

Supplementary Fig. 6 Correlations between the number of high-quality MAGs and other characteristics generated by assembly or binning process. The statistical significance analysis was based on the Pearson method. The number of obtained high-quality MAGs showed a highly significant positive correlation ($R = 0.95$, $p = 9e^{-5}$) with the number of contigs greater than or equal to 100 Kbp in length. However, the number of high-quality MAGs showed no statistical correlation ($p \geq 0.05$) with the other five values, including the number of ≥1.5 Kbp contigs, ≥10 Kbp contigs, bined contigs, total bins and medium-quality MAGs.

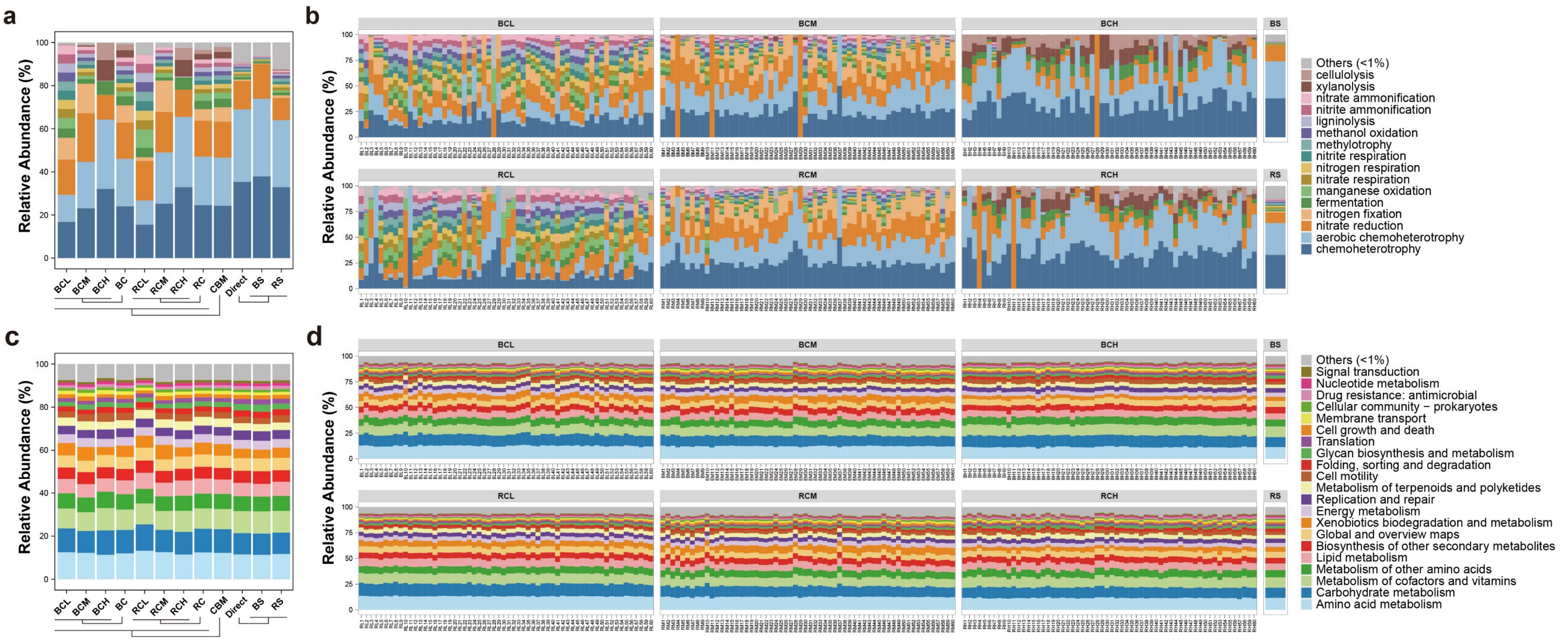

Supplementary Fig. 7 Distribution of the functions predicted by FAPROTAX and PICRUSt2. a, b showing the predicted results by FAPROTAX of grouped or all samples respectively. **c, d** showing the predicted results by PICRUSt2 of grouped or all samples respectively. The functional groups or KEGG orthology with less than 1 % relative abundance were grouped to "Others". BS (original bulk soil) and RS (original rhizosphere soil); Other abbreviations are the same as those illustrated in Supplementary Fig. 1.

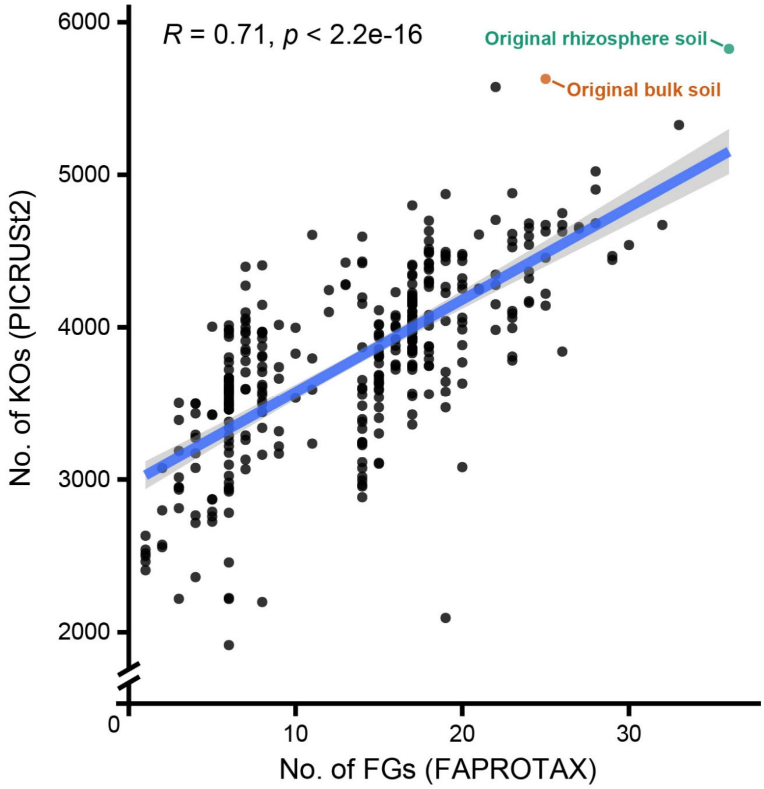

Supplementary Fig. 8 The correlation between the results predicted by FAPROTAX and PICRUSt2. Each dot indicates an individual sample. Two original soil samples are labeled and the others are culture-enriched samples. FGs, functional groups; KOs, KEGG orthology.

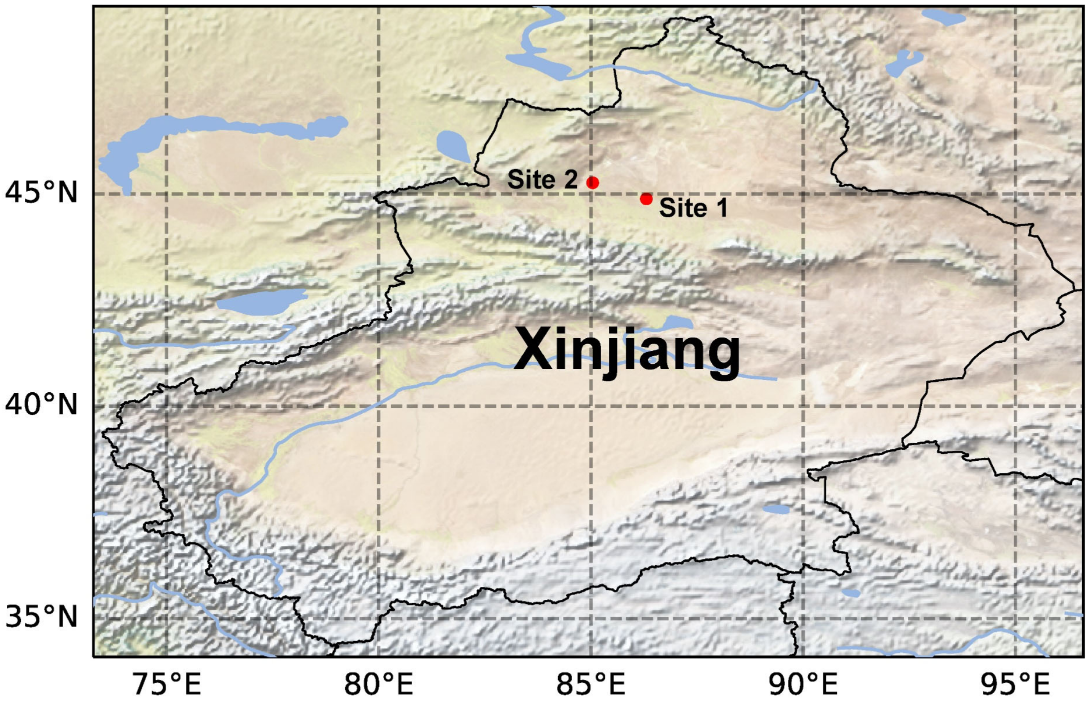

Supplementary Fig. 9 Diagram of sampling sites in the Gurbantunggut Desert. A total of seven soil samples were collected from two sampling sites (Site 1 and Site 2). Of which, one bulk soil and two rhizosphere soils of two plant species (*Haloxylon ammodendron* and *Calligonum leucocladum*) were collected from Site 1, while one bulk soil and three rhizosphere soils of three plant species (*Tamarix chinensis*, *Populus euphratica* and *Haloxylon ammodendron*) were collected from Site 2.

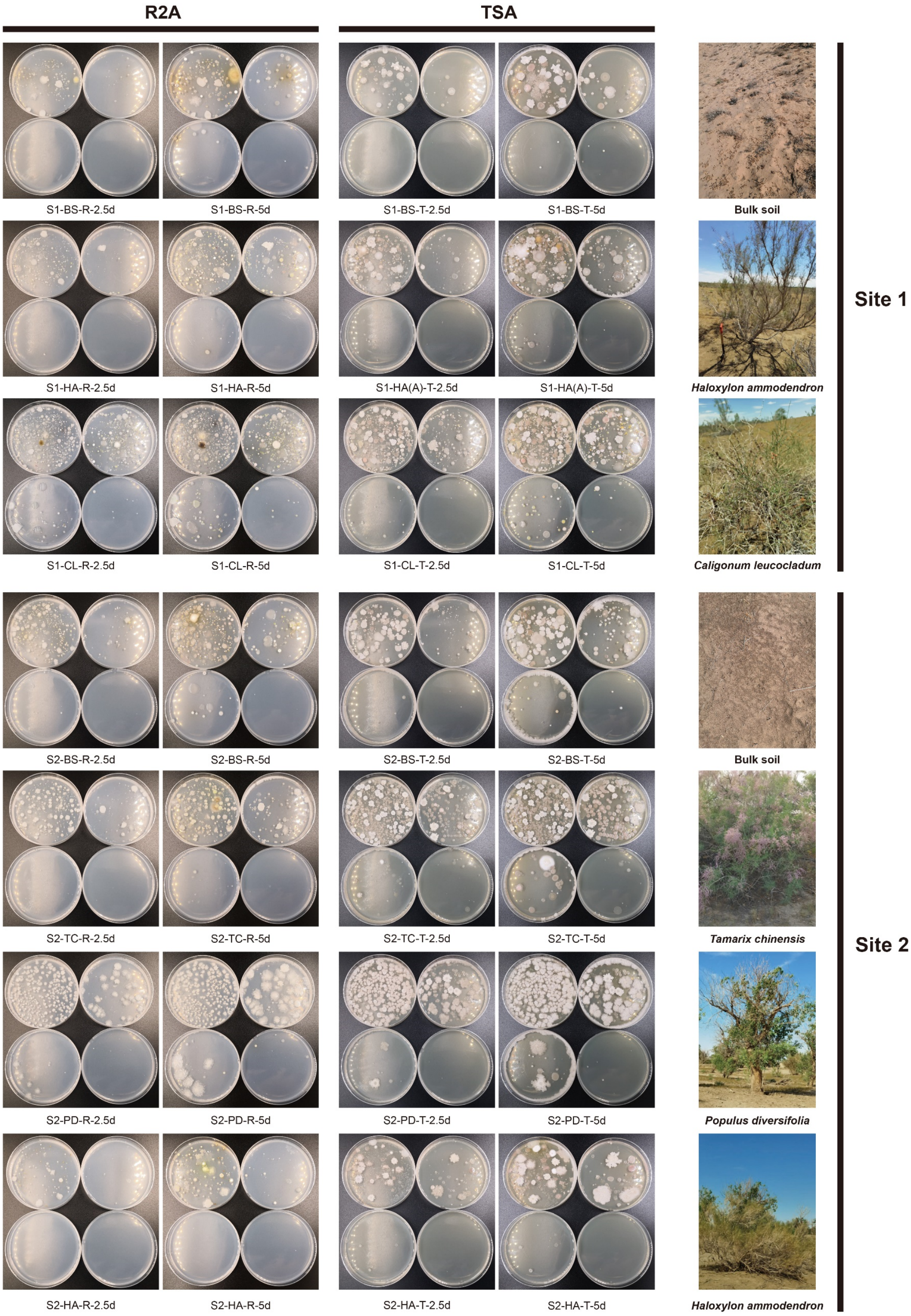

Supplementary Fig. 10 The pre-experiment results for the pilot screening of soil samples. All the seven soil samples collected from two different sites were used for the pre-experiment of sample selection, and two different agar media (R2A and TSA) were applied for test the culturability (mainly evaluated through the colony number and morphological diversity) of bacteria in these soils. All agar plates were imaged at 2.5 and 5 days of incubation time. Finally, we selected the rhizosphere soil of *Calligonum leucocladum* for the subsequent experiments since its significant advantages in both colony number and bacterial diversity, as well as the bulk soil of Site 1.

Supplementary Tables

Supplementary Table 1 Alpha diversity of the two original soil samples and six subgroups of culture-enriched samples.

Sample	Observed ASVs	Shannon	Inverse-Simpson	Pielou	PD
BS	561	8.4	1.0	0.9	112
RS	465	7.9	1.0	0.9	84.9
BCL	41.6 ± 18.0	3.3 ± 0.9	0.8 ± 0.1	0.6 ± 0.1	7.9 ± 2.9
BCM	48.8 ± 15.8	3.2 ± 0.8	0.7 ± 0.2	0.6 ± 0.1	8.8 ± 3.1
BCH	37.9 ± 16.6	3.5 ± 0.7	0.8 ± 0.1	0.7 ± 0.1	5.4 ± 2.1
RCL	50.5 ± 24.1	4.1 ± 0.9	0.9 ± 0.1	0.7 ± 0.1	8.9 ± 3.7
RCM	61.6 ± 27.8	3.1 ± 1.0	0.7 ± 0.2	0.5 ± 0.2	11.4 ± 4.0
RCH	56.4 ± 26.2	4.2 ± 0.9	0.9 ± 0.1	0.7 ± 0.1	7.8 ± 3.2

Pielou, Pielou's evenness; PD, Phylogenetic diversity; Other abbreviations are the same as those illustrated in Supplementary Fig. 1.

Supplementary Table 2 PERMANOVA results of Bray–Curtis dissimilarity between cultures of bulk and rhizosphere soils and across the whole dataset.

Culture-enriched samples	Factor	df	Sums of Sqs	R^2	Pseudo-F	p-value	Sig.
Total cultures	Soil	1	8.9866	0.0630	35.0095	0.001	***
(n = 360)	Medium	59	28.3091	0.1983	1.8692	0.001	***
	Temperature	2	29.2141	**0.2047**	56.9050	0.001	***
	Residual	297	76.2374	0.5341			
	Total	359	142.7473	1.0000			
Bulk soil cultures	Medium	59	23.5965	**0.3697**	2.0010	0.001	***
(n = 180)	temperature	2	16.6480	0.2608	41.6476	0.001	***
	Residual	118	23.5843	0.3695			
	Total	179	63.8288	1.0000			
Rhizosphere soil cultures	Medium	59	18.4329	0.2636	1.2561	0.001	***
(n = 180)	Temperature	2	22.1500	**0.3167**	44.5280	0.001	***
	Residual	118	29.3489	0.4197			
	Total	179	69.9318	1.0000			

Note: Sig. indicates significance; "***", $p = 0.001$; The highest R^2 under each group is displayed in bold.

Supplementary Table 3 The number and proportion of potentially novel bacterial taxa cultivated on different media.

Medium No.	Medium name	Average number			Average proportion (%)		
		Total	BC	RC	Total	BC	RC
M1	BEN	14.2	7.0	21.3	29.6	21.2	38.0
M2	BHI	6.3	4.0	8.7	15.7	11.3	20.2
M3	BRA	6.0	5.0	7.0	12.4	8.9	15.9
M4	CSA	4.3	0.3	8.3	15.6	1.6	29.7
M5	CHIA	17.3	11.7	23.0	23.2	20.0	26.4
M6	CZA	6.7	6.0	7.3	14.7	15.5	13.9
M7	GN1	12.5	9.0	16.0	20.7	17.8	23.5
M8	GYM	10.2	7.0	13.3	26.7	24.7	28.7
M9	GLA	5.5	4.3	6.7	11.8	9.9	13.6
M10	GGA	10.0	9.0	11.0	20.4	16.3	24.5
M11	HA	2.7	1.3	4.0	11.3	6.4	16.2
M12	HVA	15.0	7.7	22.3	26.2	20.1	32.4
M13	KB	13.8	9.3	18.3	21.6	16.1	27.2
M14	LNMS	12.2	9.0	15.3	19.9	17.2	22.7
M15	LB	14.5	8.0	21.0	26.4	20.5	32.2
M16	MCA	6.5	3.7	9.3	17.8	17.3	18.2
M17	MYP	10.8	4.0	17.7	18.4	11.1	25.7
M18	MIC	10.0	8.3	11.7	21.8	21.8	21.7
M19	MM	13.0	8.0	18.0	19.2	12.7	25.6
M20	PYG	9.2	8.0	10.3	20.7	18.1	23.3
M21	PYS	13.7	8.3	19.0	22.5	18.6	26.4
M22	PPS	8.2	6.3	10.0	16.2	13.8	18.7
M23	PDA	9.0	6.0	12.0	20.1	14.4	25.8
M24	RA	5.5	4.7	6.3	10.7	8.5	12.8
M25	RH	6.7	5.0	8.3	13.1	10.4	15.9
M26	RZ1	7.0	3.7	10.3	18.8	16.8	20.8
M27	RZ2	5.8	6.3	5.3	15.4	9.6	21.3
M28	SA	5.0	2.7	7.3	17.3	10.8	23.7
M29	SSM	4.2	3.0	5.3	14.4	10.3	18.4
M30	SSTY	8.7	4.0	13.3	21.3	14.2	28.4
M31	CMCA	6.5	3.3	9.7	12.3	11.2	13.4
M32	SLA	5.8	6.0	5.7	14.3	12.7	15.8
M33	SEAB	12.3	7.0	17.7	23.7	18.2	29.2
M34	SEAR	15.7	11.7	19.7	26.3	24.1	28.5
M35	SCA	5.7	3.3	8.0	14.0	13.1	15.0
M36	SGCY	7.2	6.3	8.0	19.7	14.4	25.1
M37	SNA	6.5	3.0	10.0	18.8	6.6	31.1
M38	STA	11.8	10.7	13.0	19.8	21.0	18.5
M39	STBG	17.8	9.3	26.3	28.5	20.5	36.6
M40	T5	9.7	6.7	12.7	20.0	14.4	25.5
M41	TP	5.0	0.7	9.3	13.3	3.0	23.5
M42	TGY	7.2	5.0	9.3	18.5	11.6	25.4
M43	TCN	6.3	4.3	8.3	12.5	8.6	16.4
M44	YGCP	15.0	10.3	19.7	25.4	20.8	30.0
M45	YMG	8.2	4.3	12.0	17.0	12.3	21.8
M46	1×TSA	9.7	3.7	15.7	20.1	13.2	27.0
M47	0.1×TSA	10.5	8.3	12.7	20.9	18.5	23.4
M48	0.01×TSA	16.8	10.0	23.7	21.0	17.3	24.7
M49	1×NA	11.7	10.0	13.3	22.2	21.6	22.8
M50	0.1×NA	12.3	14.3	10.3	19.9	23.6	16.1
M51	0.01×NA	15.3	11.7	19.0	20.9	18.3	23.5
M52	1×AIA	19.8	14.3	25.3	25.1	25.0	25.2
M53	0.1×AIA	19.2	15.3	23.0	27.3	28.5	26.2
M54	0.01×AIA	14.3	12.3	16.3	24.0	23.3	24.7
M55	1×R2A	12.7	7.7	17.7	20.5	14.8	26.2
M56	0.1×R2A	12.8	7.7	18.0	17.4	12.9	22.0
M57	0.01×R2A	10.3	8.3	12.3	17.1	15.3	18.9
M58	1×MA	16.7	10.0	23.3	24.9	18.1	31.6
M59	0.1×MA	19.5	20.0	19.0	21.1	24.8	17.3
M60	0.01×MA	12.8	7.3	18.3	16.2	12.8	19.5

Abbreviations: BC (bulk soil cultures) and RC (rhizosphere cultures).

Supplementary Table 4 Sanger sequencing results for colonies re-isolated from frozen bacterial stocks of BM11 and RM11.

Isolate ID	GenBank acc. no.	NCBI Blast						Local Blast	
		**Identity**	**Top-hit species**	**Query (nt)**	**Max score**	**Query cover**	**E-value**	**Top-hit ASV**	**Local blast identity (%)**
BM11-1	OQ146905	99.79%	*Oceanobacillus kimchii* X50^{T}	1415	2591	99%	0	ASV11	1412/1412 (100%)
BM11-2	OQ146906	99.79%	*Oceanobacillus kimchii* X50^{T}	1416	2593	99%	0	ASV11	1413/1413 (100%)
BM11-3	OQ146907	99.93%	*Bacillus stercoris* JCM 30051^{T}	1395	2571	100%	0	ASV28/ASV645	1395/1395 (100%)
BM11-4	OQ146908	99.93%	*Bacillus stercoris* JCM 30051^{T}	1395	2571	100%	0	ASV28/ASV645	1395/1395 (100%)
BM11-5	OQ146909	99.93%	*Bacillus stercoris* JCM 30051^{T}	1395	2571	100%	0	ASV28/ASV645	1395/1395 (100%)
BM11-6	OQ146910	99.93%	*Bacillus stercoris* JCM 30051^{T}	1395	2571	100%	0	ASV28/ASV645	1395/1395 (100%)
BM11-7	OQ146911	99.93%	*Bacillus stercoris* JCM 30051^{T}	1395	2571	100%	0	ASV28/ASV645	1395/1395 (100%)
BM11-8	OQ146912	99.93%	*Bacillus haynesii* NRRL B-41327^{T}	1399	2579	100%	0	ASV2	1399/1399 (100%)
BM11-9	OQ146913	99.93%	*Bacillus stercoris* JCM 30051^{T}	1395	2571	100%	0	ASV28/ASV645	1395/1395 (100%)
BM11-10	OQ146914	99.93%	*Bacillus stercoris* JCM 30051^{T}	1395	2571	100%	0	ASV28/ASV645	1395/1395 (100%)
BM11-11	OQ146915	99.79%	*Oceanobacillus kimchii* X50^{T}	1414	2595	100%	0	ASV11	1414/1414 (100%)
BM11-12	OQ146916	99.93%	*Bacillus stercoris* JCM 30051^{T}	1399	2579	100%	0	ASV28/ASV645	1399/1399 (100%)
BM11-13	OQ146917	99.93%	*Bacillus haynesii* NRRL B-41327^{T}	1400	2580	100%	0	ASV2	1400/1400 (100%)
BM11-14	OQ146918	99.93%	*Bacillus stercoris* JCM 30051^{T}	1395	2571	100%	0	ASV28/ASV645	1395/1395 (100%)
BM11-15	OQ146919	99.93%	*Bacillus stercoris* JCM 30051^{T}	1395	2571	100%	0	ASV28/ASV645	1395/1395 (100%)
BM11-16	OQ146920	99.79%	*Oceanobacillus kimchii* X50^{T}	1415	2597	100%	0	ASV11	1415/1415 (100%)
BM11-17	OQ146921	99.93%	*Bacillus stercoris* JCM 30051^{T}	1395	2571	100%	0	ASV28/ASV645	1395/1395 (100%)
BM11-18	OQ146922	99.79%	*Oceanobacillus kimchii* X50^{T}	1415	2597	100%	0	ASV11	1415/1415 (100%)
BM11-19	OQ146923	99.93%	*Bacillus stercoris* JCM 30051^{T}	1395	2571	100%	0	ASV28/ASV645	1395/1395 (100%)
BM11-20	OQ146924	99.79%	*Oceanobacillus kimchii* X50^{T}	1414	2595	100%	0	ASV11	1414/1414 (100%)
BM11-21	OQ146925	99.93%	*Bacillus cabrialesii* TE3^{T}	1399	2579	100%	0	ASV81	1399/1399 (100%)
BM11-22	OQ146926	99.22%	*Oceanobacillus polygoni* SA9^{T}	1408	2540	100%	0	ASV1251	1408/1408 (100%)
BM11-23	OQ146927	99.22%	*Oceanobacillus polygoni* SA9^{T}	1408	2540	100%	0	ASV1251	1408/1408 (100%)
BM11-24	OQ146928	99.93%	*Bacillus stercoris* JCM 30051^{T}	1399	2579	100%	0	ASV28/ASV645	1399/1399 (100%)
BM11-25	OQ146929	99.79%	*Oceanobacillus kimchii* X50^{T}	1414	2595	100%	0	ASV11	1414/1414 (100%)
BM11-26	OQ146930	99.79%	*Oceanobacillus kimchii* X50^{T}	1415	2597	100%	0	ASV11	1415/1415 (100%)
RM11-1	OQ146931	98.08%	*Gracilibacillus massiliensis* Awa-1^{T}	1405	2446	100%	0	ASV98	1405/1405 (100%)
RM11-2	OQ146932	99.72%	*Oceanobacillus kimchii* X50^{T}	1415	2591	100%	0	ASV131	1415/1415 (100%)
RM11-3	OQ146933	99.93%	*Bacillus cabrialesii* TE3^{T}	1399	2579	100%	0	ASV81	1399/1399 (100%)
RM11-4	OQ146934	100.00%	*Bacillus mojavensis* IFO 15718^{T}	1399	2584	100%	0	ASV16	1399/1399 (100%)
RM11-5	OQ146935	99.93%	*Bacillus stercoris* JCM 30051^{T}	1398	2577	100%	0	ASV28/ASV645	1398/1398 (100%)
RM11-6	OQ146936	99.93%	*Bacillus stercoris* JCM 30051^{T}	1349	2486	100%	0	ASV28/ASV645	1349/1349 (100%)
RM11-7	OQ146937	99.71%	*Priestia endophytica* 2DT^{T}	1382	2534	100%	0	ASV416	1382/1382 (100%)
RM11-8	OQ146938	99.72%	*Oceanobacillus kimchii* X50^{T}	1415	2591	100%	0	ASV131	1415/1415 (100%)
RM11-9	OQ146939	99.65%	*Halobacillus sediminis* NGS-2^{T}	1416	2588	100%	0	ASV608/ASV631/ASV648/ASV980/ASV2778	1371/1416 (97%)
RM11-10	OQ146940	99.93%	*Bacillus atrophaeus* JCM 9070^{T}	1383	2549	100%	0	ASV447	1383/1383 (100%)
RM11-11	OQ146941	99.72%	*Oceanobacillus kimchii* X50^{T}	1414	2590	100%	0	ASV131	1414/1414 (100%)
RM11-12	OQ146942	99.72%	*Oceanobacillus kimchii* X50^{T}	1414	2590	100%	0	ASV131	1414/1414 (100%)
RM11-13	OQ146943	99.93%	*Bacillus stercoris* JCM 30051^{T}	1399	2579	100%	0	ASV28/ASV645	1399/1399 (100%)
RM11-14	OQ146944	99.93%	*Bacillus stercoris* JCM 30051^{T}	1399	2579	100%	0	ASV28/ASV645	1399/1399 (100%)
RM11-15	OQ146945	100.00%	*Bacillus stercoris* JCM 30051^{T}	1393	2573	100%	0	ASV16	1393/1393 (100%)
RM11-16	OQ146946	99.72%	*Oceanobacillus kimchii* X50^{T}	1414	2582	99%	0	ASV131	1410/1410 (100%)
RM11-17	OQ146947	99.72%	*Oceanobacillus kimchii* X50^{T}	1410	2582	100%	0	ASV131	1410/1410 (100%)
RM11-18	OQ146948	99.93%	*Bacillus stercoris* JCM 30051^{T}	1394	2569	100%	0	ASV28/ASV645	1394/1394 (100%)
RM11-19	OQ146949	97.22%	*Brevibacterium sanguinis* CF63^{T}	1370	2311	99%	0	ASV20	1370/1370 (100%)
RM11-20	OQ146950	98.78%	*Planococcus halotolerans* SCU63^{T}	1391	2508	100%	0	ASV708	1380/1391 (99%)
RM11-21	OQ146951	99.93%	*Bacillus cabrialesii* TE3^{T}	1391	2564	100%	0	ASV81	1391/1391 (100%)
RM11-22	OQ146952	99.72%	*Oceanobacillus kimchii* X50^{T}	1410	2582	100%	0	ASV131	1410/1410 (100%)
RM11-23	OQ146953	97.52%	*Brevibacterium sanguinis* CF63^{T}	1372	2333	99%	0	ASV1347	1372/1372 (100%)
RM11-24	OQ146954	99.93%	*Bacillus stercoris* JCM 30051^{T}	1394	2569	100%	0	ASV28/ASV645	1394/1394 (100%)
RM11-25	OQ146955	100.00%	*Bacillus mojavensis* IFO 15718^{T}	1394	2575	100%	0	ASV80	1394/1394 (100%)
RM11-26	OQ146956	97.52%	*Brevibacterium sanguinis* CF63^{T}	1372	2333	99%	0	ASV1347	1372/1372 (100%)
RM11-27	OQ146957	98.92%	*Planococcus halotolerans* SCU63^{T}	1396	2471	99%	0	ASV708	1383/1396 (99%)
RM11-28	OQ146958	98.92%	*Planococcus halotolerans* SCU63^{T}	1394	2470	99%	0	ASV708	1381/1394 (99%)

Supplementary Table 5 Summary of sequencing, assembly and binning results of nine metagenomic samples.

Characteristic	BS	RS	RL56	RL58	RM44	RM46	RM56	RH52	RH59
Bases (Gbp)	31.24	37.42	35.62	33.06	30.86	30.25	35.29	30.47	35.92
Paired reads (Millions)	104.56	125.02	119.00	110.99	103.12	101.37	118.01	101.86	120.01
Contigs									
≥1.5 Kbp	147617	125540	89353	74022	91070	66245	100375	59308	36902
≥10 Kbp	1120	636	3559	3737	5122	2975	5402	5435	4529
≥100 Kbp	12	4	55	49	109	50	93	115	221
Binned contigs	114200	97766	43336	37410	51074	35379	59862	29074	24129
Total bins	38	39	61	71	88	60	89	78	56
High-quality MAGs	**1**	**0**	**3**	**1**	**6**	**2**	**5**	**3**	**12**
Medium-quality MAGs	10	10	10	9	14	12	16	21	13
No. of domains	1	2	1	1	1	1	1	1	1
No. of phyla	4	5	4	2	3	3	2	2	3
No. of classes	6	7	4	2	3	3	2	3	4
No. of orders	7	8	4	2	6	4	5	3	10

Abbreviations: BC (bulk soil cultures) and RC (rhizosphere cultures).

Supplementary Table 6 Summary of all the high-quality and medium-quality MAGs obtained in this study.

Bin ID	Completeness (%)	Contamination (%)	Strain heterogeneity (%)	No. of contigs	Genome size (bp)	coverage (x)	*N*50 (bp)	CDS	Coding density (%)	GC (%)	tRNA	5S rRNA	16S rRNA	23S rRNA	sRNA	CRISPR array	fastani_ani	Taxonomy
BS_bin.004	71.61	2.45	36.84	408	2104626	176	5753	2366	85.72	59.96	25	0	1	0	1	0	N/A	d__Bacteria;p__Proteobacteria;c__Gammaproteobacteria;o__Ectothiorhodospirales;f__;g__;s__
BS_bin.006	66.41	3.56	16.67	695	2137925	173	3435	2690	92.45	70.87	29	1	0	0	1	0	N/A	d__Bacteria;p__Actinobacteriota;c__UBA4738;o__UBA4738;f__HRBIN12;g__;s__
BS_bin.015	97.86	0.28	100	31	3150515	117	195896	3187	94.03	73.11	49	1	1	1	2	0	80.79	d__Bacteria;p__Actinobacteriota;c__Thermoleophilia;o__Solirubrobacterales;f__Solirubrobacteraceae;g__;s__
BS_bin.016	51.44	3.4	50	808	2427524	152	3211	2848	92.51	65.67	16	0	0	0	0	0	N/A	d__Bacteria;p__Gemmatimonadota;c__Gemmatimonadetes;o__Gemmatimonadales;f__Gemmatimonadaceae;g__;s__
BS_bin.017	53.61	2.41	28.57	511	1327022	279	2645	1726	93.22	71.30	18	2	0	0	1	0	N/A	d__Bacteria;p__Actinobacteriota;c__Thermoleophilia;o__Solirubrobacterales;f__Thermoleophilaceae;g__;s__
BS_bin.021	50.22	7.67	30.43	425	1165852	317	2838	1547	94.51	71.00	12	0	0	0	0	0	N/A	d__Bacteria;p__Actinobacteriota;c__Thermoleophilia;o__Gaiellales;f__Gaiellaceae;g__;s__
BS_bin.023	80.77	1.47	50	417	2230629	166	7166	2525	91.55	71.48	36	1	0	0	0	0	79.18	d__Bacteria;p__Chloroflexota;c__Ellin6529;o__QHBO01;f__QHBO01;g__CF-167;s__
BS_bin.026	56.67	2.64	60	408	1207314	306	3185	1548	93.37	65.48	25	0	0	0	0	0	N/A	d__Bacteria;p__Actinobacteriota;c__UBA4738;o__UBA4738;f__UBA4738;g__;s__
BS_bin.030	64.02	7.76	27.27	691	2221209	166	3476	2899	92.67	70.95	31	1	0	0	0	0	N/A	d__Bacteria;p__Actinobacteriota;c__Thermoleophilia;o__Gaiellales;f__;g__;s__
BS_bin.037	82.26	3.42	20	621	3477731	106	7170	3946	93.04	69.58	31	0	0	0	0	0	76.96	d__Bacteria;p__Actinobacteriota;c__Acidimicrobiia;o__IMCC26256;f__PALSA-555;g__;s__
BS_bin.038	68.62	3.85	41.67	578	1634545	226	2998	2182	92.89	71.24	28	0	0	0	0	0	N/A	d__Bacteria;p__Actinobacteriota;c__UBA4738;o__UBA4738;f__;g__;s__
RS_bin.003	96.12	6.8	0	131	2322072	129	36692	2476	75.10	33.27	44	1	0	0	1	0	80.11	d__Archaea;p__Thermoproteota;c__Nitrososphaeria;o__Nitrososphaerales;f__Nitrososphaeraceae;g__Nitrosocosmicus;s__
RS_bin.004	52.4	0	0	414	1800992	166	4597	2090	86.65	55.86	15	0	0	0	0	0	N/A	d__Bacteria;p__Nitrospirota;c__Nitrospiria;o__Nitrospirales;f__;g__;s__
RS_bin.006	71.58	7.65	8.33	1003	2873341	104	3023	3876	95.45	66.62	24	0	0	0	1	0	79.85	d__Bacteria;p__Proteobacteria;c__Gammaproteobacteria;o__Burkholderiales;f__SG8-39;g__SCGC-AG-212-J23;s__
RS_bin.016	67.57	6.41	9.09	1425	4701967	64	3666	4973	92.18	67.42	19	0	0	0	1	0	79.39	d__Bacteria;p__Acidobacteriota;c__Thermoanaerobaculia;o__Gp7-AA8;f__Gp7-AA8;g__QHVT01;s__
RS_bin.021	58.32	2.59	75	592	1736965	172	3111	2231	93.08	72.60	12	0	0	0	0	0	N/A	d__Bacteria;p__Actinobacteriota;c__Thermoleophilia;o__Gaiellales;f__;g__;s__
RS_bin.030	55.08	1.75	66.67	502	1533162	195	3291	1951	87.91	63.90	26	0	0	0	0	0	77.42	d__Bacteria;p__Actinobacteriota;c__Rubrobacteria;o__Rubrobacterales;f__Rubrobacteraceae;g__SIRX01;s__
RS_bin.031	59.26	5.45	55.56	335	1678343	178	5722	1831	80.54	56.36	21	0	0	0	1	0	N/A	d__Bacteria;p__Proteobacteria;c__Gammaproteobacteria;o__Ectothiorhodospirales;f__J044;g__;s__
RS_bin.032	78.72	5.83	28.57	397	2006643	149	6111	2485	75.52	35.82	36	1	0	0	0	0	N/A	d__Archaea;p__Thermoproteota;c__Nitrososphaeria;o__Nitrososphaerales;f__Nitrososphaeraceae;g__;s__
RS_bin.033	56.3	2.14	33.33	932	2354141	127	2601	3075	90.47	71.64	31	1	0	0	1	0	77.78	d__Bacteria;p__Actinobacteriota;c__Acidimicrobiia;o__Acidimicrobiales;f__AC-14;g__;s__
RS_bin.039	60.53	9.3	55.93	434	1969053	152	5017	2240	79.02	56.59	20	0	0	0	1	0	N/A	d__Bacteria;p__Proteobacteria;c__Gammaproteobacteria;o__Ectothiorhodospirales;f__c;g__;s__
RL56_bin.009	99.72	1.3	0	23	3159331	103	239842	2809	90.05	70.77	51	3	0	0	0	0	81.27	d__Bacteria;p__Actinobacteriota;c__Actinomycetia;o__Actinomycetales;f__Micrococcaceae;g__Zhihengliuella;s__
RL56_bin.011	68.1	4.31	100	207	2826505	115	23528	2735	90.59	64.80	37	0	0	0	1	0	87.35	d__Bacteria;p__Actinobacteriota;c__Actinomycetia;o__Actinomycetales;f__Micrococcaceae;g__Pseudarthrobacter;s__
RL56_bin.023	92.24	2.19	42.86	221	3003528	108	22858	2950	91.00	63.83	44	0	0	0	0	0	81.36	d__Bacteria;p__Actinobacteriota;c__Actinomycetia;o__Actinomycetales;f__Micrococcaceae;g__Arthrobacter_D;s__
RL56_bin.032	70.69	5.17	50	219	3735861	87	40567	3623	90.76	66.40	51	2	0	0	1	0	92.76	d__Bacteria;p__Actinobacteriota;c__Actinomycetia;o__Actinomycetales;f__Micrococcaceae;g__Pseudarthrobacter;s__
RL56_bin.036	77.2	2.79	92.31	594	2189524	148	3980	2583	90.80	69.15	29	0	0	0	4	0	86.09	d__Bacteria;p__Proteobacteria;c__Alphaproteobacteria;o__Caulobacterales;f__Caulobacteraceae;g__Brevundimonas;s__
RL56_bin.039	50.89	1.72	100	834	2825771	115	3645	3235	90.39	73.83	30	0	0	0	1	0	97.86	d__Bacteria;p__Actinobacteriota;c__Actinomycetia;o__Actinomycetales;f__Micrococcaceae;g__Kocuria;s__Kocuria rosea
RL56_bin.040	69.94	7.02	83.33	618	2527798	128	4811	2912	93.32	68.53	29	1	0	0	0	0	82.31	d__Bacteria;p__Actinobacteriota;c__Actinomycetia;o__Actinomycetales;f__Microbacteriaceae;g__Microbacterium;s__
RL56_bin.043	98.28	7.18	94.44	74	4239492	76	137506	4008	91.02	61.76	53	1	0	0	1	0	91.80	d__Bacteria;p__Actinobacteriota;c__Actinomycetia;o__Actinomycetales;f__Micrococcaceae;g__Paenarthrobacter;s__
RL56_bin.044	96.7	0.27	0	113	4329954	75	82535	4103	89.23	62.08	55	1	0	0	1	1	N/A	d__Bacteria;p__Actinobacteriota;c__Actinomycetia;o__Actinomycetales;f__Micrococcaceae;g__Arthrobacter_I;s__
RL56_bin.045	51.72	0	0	106	2453387	132	31875	2440	83.11	41.49	51	3	0	0	3	0	96.95	d__Bacteria;p__Firmicutes;c__Bacilli;o__Bacillales;f__Bacillaceae_A;g__Bacillus_X;s__Bacillus_X frigoritolerans
RL56_bin.047	77.59	1.72	0	372	3754071	86	14257	3916	85.05	38.66	49	6	0	0	5	0	82.82	d__Bacteria;p__Firmicutes;c__Bacilli;o__Bacillales;f__Bacillaceae_A;g__Bacillus_AC;s__
RL56_bin.054	74.29	2.59	100	392	3945686	82	12614	3761	91.35	41.37	31	0	0	0	0	0	79.26	d__Bacteria;p__Bacteroidota;c__Bacteroidia;o__Sphingobacteriales;f__Sphingobacteriaceae;g__Pedobacter;s__
RL56_bin.055	64.67	2.47	75	518	1808750	179	3943	2117	93.76	71.14	22	0	0	0	0	0	83.63	d__Bacteria;p__Actinobacteriota;c__Actinomycetia;o__Actinomycetales;f__Microbacteriaceae;g__Microbacterium;s__
RL58_bin.009	60.79	5.17	33.33	640	2001338	140	3378	2270	91.26	74.14	24	0	0	0	1	0	87.02	d__Bacteria;p__Actinobacteriota;c__Actinomycetia;o__Actinomycetales;f__Micrococcaceae;g__Kocuria;s__
RL58_bin.014	58.04	0	0	604	4359234	64	8971	4859	85.48	35.42	17	1	0	0	8	0	97.87	d__Bacteria;p__Firmicutes;c__Bacilli;o__Bacillales;f__Bacillaceae_G;g__Bacillus_A;s__Bacillus_A anthracis
RL58_bin.017	74.46	1.15	0	15	2703124	104	241091	2417	90.00	70.96	39	3	0	0	0	0	81.23	d__Bacteria;p__Actinobacteriota;c__Actinomycetia;o__Actinomycetales;f__Micrococcaceae;g__Zhihengliuella;s__
RL58_bin.028	81.03	0	0	187	4721928	60	40450	4597	81.73	40.62	55	7	0	0	5	0	96.65	d__Bacteria;p__Firmicutes;c__Bacilli;o__Bacillales;f__Bacillaceae_A;g__Bacillus_X;s__Bacillus_X frigoritolerans
RL58_bin.033	80.61	1.99	100	446	2613055	108	10651	2728	89.70	73.68	37	0	0	0	1	0	90.38	d__Bacteria;p__Actinobacteriota;c__Actinomycetia;o__Actinomycetales;f__Micrococcaceae;g__Kocuria;s__
RL58_bin.045	77.57	3.84	43.75	528	3287022	86	7529	3530	85.54	38.96	19	2	0	0	3	0	82.82	d__Bacteria;p__Firmicutes;c__Bacilli;o__Bacillales;f__Bacillaceae_A;g__Bacillus_AC;s__
RL58_bin.046	64.11	3.94	21.43	628	1850856	152	3042	2240	93.86	63.82	45	0	0	0	0	0	83.83	d__Bacteria;p__Actinobacteriota;c__Actinomycetia;o__Actinomycetales;f__Microbacteriaceae;g__Microbacterium;s__
RL58_bin.052	94.98	2.65	37.5	233	3195510	88	23485	3163	91.23	69.20	25	0	0	0	0	0	81.61	d__Bacteria;p__Actinobacteriota;c__Actinomycetia;o__Actinomycetales;f__Micrococcaceae;g__Arthrobacter_D;s__
RL58_bin.057	62.14	2.15	50	576	1685659	167	3143	2067	93.40	61.95	45	1	0	0	1	0	82.64	d__Bacteria;p__Actinobacteriota;c__Actinomycetia;o__Actinomycetales;f__Microbacteriaceae;g__Microbacterium;s__
RL58_bin.064	81.38	1.46	66.67	63	3855687	73	151027	3642	90.34	61.95	45	4	0	0	1	0	91.65	d__Bacteria;p__Actinobacteriota;c__Actinomycetia;o__Actinomycetales;f__Micrococcaceae;g__Paenarthrobacter;s__
RM44_bin.004	96.4	4.73	36.84	79	3586665	105	135733	3508	92.31	68.02	55	0	0	0	0	0	82.79	d__Bacteria;p__Actinobacteriota;c__Actinomycetia;o__Actinomycetales;f__Microbacteriaceae;g__Microbacterium;s__
RM44_bin.005	98.91	2.44	57.14	34	3217884	117	173822	2977	92.57	70.10	48	0	0	0	0	2	82.62	d__Bacteria;p__Actinobacteriota;c__Actinomycetia;o__Actinomycetales;f__Microbacteriaceae;g__Microbacterium;s__

RM44_bin.009	91.32	3.28	80	231	2561281	148	19733	2574	92.08	73.23	44	0	0	0	0	0	86.98	d__Bacteria;p__Actinobacteriota;c__Actinomycetia;o__Actinomycetales;f__Microbacteriaceae;g__Agrococcus;s__
RM44_bin.014	98.26	0	0	44	3831447	99	142238	3412	90.31	65.86	50	1	0	0	0	6	85.22	d__Bacteria;p__Actinobacteriota;c__Actinomycetia;o__Actinomycetales;f__Brevibacteriaceae;g__Brevibacterium;s__
RM44_bin.016	92.83	0.93	66.67	247	3462013	109	20726	3235	90.94	67.90	51	1	0	0	0	0	86.25	d__Bacteria;p__Actinobacteriota;c__Actinomycetia;o__Actinomycetales;f__Brevibacteriaceae;g__Brevibacterium;s__
RM44_bin.018	85.36	7.68	17.95	717	4250686	89	7471	4709	92.73	66.71	54	1	0	0	0	0	85.42	d__Bacteria;p__Actinobacteriota;c__Actinomycetia;o__Actinomycetales;f__Microbacteriaceae;g__Microbacterium;s__
RM44_bin.022	51.71	0	0	10	1030523	367	156200	900	90.54	69.86	21	1	0	0	0	0	81.34	d__Bacteria;p__Actinobacteriota;c__Actinomycetia;o__Actinomycetales;f__Micrococcaceae;g__Zhihengliuella;s__
RM44_bin.032	62.38	0.95	33.33	870	2551107	148	3030	3139	92.35	73.48	32	0	0	0	0	0	82.14	d__Bacteria;p__Actinobacteriota;c__Actinomycetia;o__Propionibacteriales;f__Nocardioidaceae;g__Nocardioides;s__
RM44_bin.034	94.98	0.72	33.33	173	5005351	76	49493	4755	87.59	62.73	39	0	0	0	20	0	98.88	d__Bacteria;p__Proteobacteria;c__Alphaproteobacteria;o__Rhizobiales;f__Rhizobiaceae;g__Sinorhizobium;s__Sinorhizobium meliloti_A
RM44_bin.035	66.61	8.84	65.85	403	2830568	134	8572	3022	90.44	62.69	38	0	0	0	0	0	79.74	d__Bacteria;p__Actinobacteriota;c__Actinomycetia;o__Actinomycetales;f__Micrococcaceae;g__Arthrobacter_D;s__
RM44_bin.046	81.02	1.46	50	317	2651943	143	13527	2647	89.75	73.98	44	1	0	0	1	0	86.21	d__Bacteria;p__Actinobacteriota;c__Actinomycetia;o__Actinomycetales;f__Micrococcaceae;g__Kocuria;s__
RM44_bin.050	88.54	5.78	43.75	660	3199727	118	6042	3454	91.43	75.80	40	0	0	0	1	0	86.27	d__Bacteria;p__Actinobacteriota;c__Actinomycetia;o__Actinomycetales;f__Cellulomonadaceae;g__Cellulomonas;s__
RM44_bin.051	56.9	1.72	0	418	2474124	153	6552	2672	85.28	38.85	9	2	0	0	2	0	82.64	d__Bacteria;p__Firmicutes;c__Bacilli;o__Bacillales;f__Bacillaceae_A;g__Bacillus_AC;s__
RM44_bin.060	74.14	0	0	343	3246240	116	11491	3301	83.81	41.24	30	4	0	0	4	0	96.88	d__Bacteria;p__Firmicutes;c__Bacilli;o__Bacillales;f__Bacillaceae_A;g__Bacillus_X;s__Bacillus_X frigoritolerans
RM44_bin.067	59.48	0	0	271	2503470	151	12857	2538	91.42	69.12	35	0	0	0	0	0	81.52	d__Bacteria;p__Actinobacteriota;c__Actinomycetia;o__Actinomycetales;f__Micrococcaceae;g__Arthrobacter_D;s__
RM44_bin.069	84.98	2.4	83.33	313	2369406	160	11522	2469	93.01	63.99	36	0	0	0	0	0	82.76	d__Bacteria;p__Actinobacteriota;c__Actinomycetia;o__Actinomycetales;f__Microbacteriaceae;g__Microbacterium;s__
RM44_bin.073	78.11	7.08	20	662	3138098	120	5736	3402	92.23	73.18	46	1	0	0	1	0	90.88	d__Bacteria;p__Actinobacteriota;c__Actinomycetia;o__Actinomycetales;f__Actinomycetaceae;g__Oceanitalea;s__
RM44_bin.074	62.22	2.42	50	421	2031538	186	6016	2378	92.62	66.54	26	0	0	0	2	0	88.23	d__Bacteria;p__Proteobacteria;c__Alphaproteobacteria;o__Rhodobacterales;f__Rhodobacteraceae;g__Paracoccus;s__
RM44_bin.079	91.23	6.63	27.91	1152	6204841	61	6760	6374	88.19	73.43	56	1	0	0	15	0	87.88	d__Bacteria;p__Actinobacteriota;c__Actinomycetia;o__Streptomycetales;f__Streptomycetaceae;g__Streptomyces;s__
RM44_bin.080	66.82	2.3	50	374	3430740	110	14783	3321	90.67	75.78	38	0	0	0	1	1	86.66	d__Bacteria;p__Actinobacteriota;c__Actinomycetia;o__Actinomycetales;f__Cellulomonadaceae;g__Cellulomonas;s__
RM46_bin.003	72.41	1.72	100	540	3661137	68	8958	3784	88.26	62.95	19	0	0	0	13	0	98.80	d__Bacteria;p__Proteobacteria;c__Alphaproteobacteria;o__Rhizobiales;f__Rhizobiaceae;g__Sinorhizobium;s__Sinorhizobium meliloti_A
RM46_bin.004	63.79	0	0	137	1756220	142	21826	1692	89.91	65.30	29	0	0	0	0	0	83.31	d__Bacteria;p__Actinobacteriota;c__Actinomycetia;o__Actinomycetales;f__Micrococcaceae;g__Arthrobacter;s__
RM46_bin.006	86.6	7.23	10	542	3246857	77	8062	3481	91.82	69.60	46	0	0	0	0	0	81.81	d__Bacteria;p__Actinobacteriota;c__Actinomycetia;o__Actinomycetales;f__Microbacteriaceae;g__Leucobacter;s__
RM46_bin.007	97.35	5.96	34.88	627	8578216	29	23611	7963	87.95	72.11	85	2	0	0	36	4	99.51	d__Bacteria;p__Actinobacteriota;c__Actinomycetia;o__Streptomycetales;f__Streptomycetaceae;g__Streptomyces;s__Streptomyces alfalfae
RM46_bin.015	80.6	6.03	40	1072	7265233	34	8885	7354	87.26	72.91	59	0	0	0	17	1	87.43	d__Bacteria;p__Actinobacteriota;c__Actinomycetia;o__Streptomycetales;f__Streptomycetaceae;g__Streptomyces;s__
RM46_bin.026	81.03	1.05	75	320	2193326	113	10247	2323	93.07	69.15	35	0	0	0	0	0	82.59	d__Bacteria;p__Actinobacteriota;c__Actinomycetia;o__Actinomycetales;f__Microbacteriaceae;g__Microbacterium;s__
RM46_bin.030	98.91	1.91	57.14	29	3365850	74	320176	3205	93.27	68.76	49	1	0	0	0	0	82.59	d__Bacteria;p__Actinobacteriota;c__Actinomycetia;o__Actinomycetales;f__Microbacteriaceae;g__Microbacterium;s__
RM46_bin.033	64.24	0.78	66.67	457	2371518	105	5952	2444	91.31	68.27	32	1	0	0	0	0	86.01	d__Bacteria;p__Actinobacteriota;c__Actinomycetia;o__Actinomycetales;f__Brevibacteriaceae;g__Brevibacterium;s__
RM46_bin.041	91.86	5.11	46.88	409	2846041	87	10729	3010	92.69	71.20	47	2	0	0	0	0	85.14	d__Bacteria;p__Actinobacteriota;c__Actinomycetia;o__Actinomycetales;f__Microbacteriaceae;g__Microbacterium;s__
RM46_bin.043	53.67	0	0	9	1293035	192	319320	1162	90.33	70.81	23	1	0	0	0	0	81.51	d__Bacteria;p__Actinobacteriota;c__Actinomycetia;o__Actinomycetales;f__Micrococcaceae;g__Zhihengliuella;s__
RM46_bin.044	91.39	0.55	50	158	2829882	88	27902	2791	84.87	34.73	23	1	0	0	11	0	80.84	d__Bacteria;p__Firmicutes;c__Bacilli;o__Bacillales;f__Planococcaceae;g__Ureibacillus;s__
RM46_bin.052	50.86	6.9	60	542	4391313	57	9776	4536	90.30	62.04	44	2	0	0	0	0	91.20	d__Bacteria;p__Actinobacteriota;c__Actinomycetia;o__Actinomycetales;f__Micrococcaceae;g__Paenarthrobacter;s__
RM46_bin.056	58.39	0	0	826	2235968	111	2800	2713	91.80	76.08	30	0	0	0	1	0	85.64	d__Bacteria;p__Actinobacteriota;c__Actinomycetia;o__Actinomycetales;f__Cellulomonadaceae;g__Cellulomonas;s__
RM46_bin.057	68.76	5.17	100	843	3908104	64	5684	4099	90.51	75.74	45	0	0	0	1	1	86.14	d__Bacteria;p__Actinobacteriota;c__Actinomycetia;o__Actinomycetales;f__Cellulomonadaceae;g__Cellulomonas;s__
RM56_bin.004	65.52	0	0	586	3987477	102	8500	4099	87.65	62.39	30	0	0	0	13	0	84.99	d__Bacteria;p__Proteobacteria;c__Alphaproteobacteria;o__Rhizobiales;f__Rhizobiaceae;g__Sinorhizobium;s__
RM56_bin.007	84.48	9.25	81.58	280	2598132	156	12569	2660	91.48	63.89	36	1	0	0	0	0	81.44	d__Bacteria;p__Actinobacteriota;c__Actinomycetia;o__Actinomycetales;f__Micrococcaceae;g__Arthrobacter_D;s__
RM56_bin.010	73.16	0.76	66.67	493	1913177	212	4541	2337	91.78	71.14	30	1	0	0	1	0	87.97	d__Bacteria;p__Proteobacteria;c__Alphaproteobacteria;o__Caulobacterales;f__Caulobacteraceae;g__Brevundimonas;s__
RM56_bin.011	65.15	0	0	590	2593573	157	4950	2759	91.23	68.32	31	1	0	0	0	0	85.77	d__Bacteria;p__Actinobacteriota;c__Actinomycetia;o__Actinomycetales;f__Brevibacteriaceae;g__Brevibacterium;s__
RM56_bin.012	79.75	3.76	76.92	504	3112897	131	7163	3131	90.71	66.35	45	1	0	0	0	1	84.88	d__Bacteria;p__Actinobacteriota;c__Actinomycetia;o__Actinomycetales;f__Brevibacteriaceae;g__Brevibacterium;s__
RM56_bin.013	99.72	1.34	20	15	3146349	129	483389	2793	90.03	70.77	51	3	0	0	0	0	81.22	d__Bacteria;p__Actinobacteriota;c__Actinomycetia;o__Actinomycetales;f__Micrococcaceae;g__Zhihengliuella;s__
RM56_bin.014	87.94	0.66	100	375	3010941	135	12071	3011	88.05	73.77	45	1	0	0	1	0	86.09	d__Bacteria;p__Actinobacteriota;c__Actinomycetia;o__Actinomycetales;f__Micrococcaceae;g__Kocuria;s__
RM56_bin.016	70.94	4.91	38.1	580	2662356	153	5295	3002	92.04	67.07	31	1	0	0	1	0	85.53	d__Bacteria;p__Proteobacteria;c__Alphaproteobacteria;o__Sphingomonadales;f__Sphingomonadaceae;g__Sphingomonas;s__
RM56_bin.024	79.97	8.61	19.35	393	3032895	134	11816	3279	93.09	72.71	38	1	0	0	0	0	92.73	d__Bacteria;p__Actinobacteriota;c__Actinomycetia;o__Propionibacteriales;f__Nocardioidaceae;g__Aeromicrobium_A;s__
RM56_bin.033	78.61	7.93	32.26	728	2482544	164	3854	2956	92.87	73.37	29	0	0	0	0	0	88.74	d__Bacteria;p__Actinobacteriota;c__Actinomycetia;o__Actinomycetales;f__Microbacteriaceae;g__Agrococcus;s__
RM56_bin.042	95.89	0.62	0	182	5231989	78	52226	4972	87.69	62.70	44	0	0	0	20	0	98.78	d__Bacteria;p__Proteobacteria;c__Alphaproteobacteria;o__Rhizobiales;f__Rhizobiaceae;g__Sinorhizobium;s__Sinorhizobium meliloti_A
RM56_bin.044	62.44	5.26	0	459	3158583	129	10373	3231	92.71	70.48	44	0	0	0	0	0	83.35	d__Bacteria;p__Actinobacteriota;c__Actinomycetia;o__Actinomycetales;f__Microbacteriaceae;g__Agromyces;s__
RM56_bin.049	98.92	1.48	33.33	108	3168910	128	48349	3066	91.41	66.43	45	1	0	0	1	0	89.86	d__Bacteria;p__Proteobacteria;c__Alphaproteobacteria;o__Sphingomonadales;f__Sphingomonadaceae;g__Altererythrobacter_A;s__
RM56_bin.052	93.77	1.78	66.67	325	2942220	138	14527	2979	93.08	70.58	40	0	0	0	0	0	84.50	d__Bacteria;p__Actinobacteriota;c__Actinomycetia;o__Actinomycetales;f__Microbacteriaceae;g__Agromyces;s__
RM56_bin.059	82	1.77	83.33	463	2563848	159	7137	2707	93.50	70.92	28	1	0	0	0	0	83.67	d__Bacteria;p__Actinobacteriota;c__Actinomycetia;o__Actinomycetales;f__Microbacteriaceae;g__Agromyces;s__
RM56_bin.062	56.9	0	0	81	3085392	132	99573	2875	91.25	64.09	43	0	0	0	1	0	N/A	d__Bacteria;p__Actinobacteriota;c__Actinomycetia;o__Actinomycetales;f__Micrococcaceae;g__Pseudarthrobacter;s__
RM56_bin.064	98.74	1.21	0	24	3163755	128	723580	3108	93.23	70.18	48	0	0	0	0	0	N/A	d__Bacteria;p__Actinobacteriota;c__Actinomycetia;o__Actinomycetales;f__Microbacteriaceae;g__;s__
RM56_bin.066	61.11	0	0	126	1937932	210	28160	1918	93.32	72.75	34	0	0	0	0	0	89.60	d__Bacteria;p__Actinobacteriota;c__Actinomycetia;o__Actinomycetales;f__Microbacteriaceae;g__Agrococcus;s__
RM56_bin.073	73.81	4.87	52	590	4567801	89	10600	4719	86.63	62.57	34	0	0	0	17	0	93.46	d__Bacteria;p__Proteobacteria;c__Alphaproteobacteria;o__Rhizobiales;f__Rhizobiaceae;g__Sinorhizobium;s__
RM56_bin.079	65.52	9.48	63.64	387	2779032	146	8299	2911	91.11	62.77	42	1	0	0	0	0	79.39	d__Bacteria;p__Actinobacteriota;c__Actinomycetia;o__Actinomycetales;f__Micrococcaceae;g__Arthrobacter_D;s__
RM56_bin.085	54.31	9.48	14.29	1493	3722569	109	2358	4539	91.92	75.28	29	0	0	0	0	0	79.80	d__Bacteria;p__Actinobacteriota;c__Actinomycetia;o__Actinomycetales;f__Cellulomonadaceae;g__Actinotalea;s__

RH52_bin.002	62.07	1.72	0	331	2613693	118	9353	2733	85.65	38.81	25	3	0	0	2	0	82.93	d__Bacteria;p__Firmicutes;c__Bacilli;o__Bacillales;f__Bacillaceae_A;g__Bacillus_AC;s__
RH52_bin.003	98.01	2.1	20	31	3333832	92	315399	3206	86.54	37.29	56	8	0	0	2	0	87.94	d__Bacteria;p__Firmicutes;c__Bacilli;o__Bacillales;f__Planococcaceae;g__Lysinibacillus;s__
RH52_bin.007	80.36	5.95	28.57	486	5426557	57	15472	5694	84.70	38.28	60	4	0	0	9	0	87.74	d__Bacteria;p__Firmicutes;c__Bacilli;o__Bacillales;f__Bacillaceae_A;g__Bacillus_W;s__
RH52_bin.008	92.75	0.59	100	60	6357382	48	175192	5638	90.29	59.55	57	5	0	0	3	0	93.16	d__Bacteria;p__Firmicutes;c__Bacilli;o__Paenibacillales;f__Paenibacillaceae;g__Paenibacillus;s__
RH52_bin.010	77.33	3.79	33.33	621	2954542	104	5884	3250	92.51	70.59	37	0	0	0	1	1	82.34	d__Bacteria;p__Actinobacteriota;c__Actinomycetia;o__Actinomycetales;f__Microbacteriaceae;g__Microbacterium;s__
RH52_bin.013	66.38	0	0	274	2139111	144	9407	2307	87.43	40.94	14	4	0	0	11	0	79.38	d__Bacteria;p__Firmicutes;c__Bacilli;o__Bacillales;f__Domibacillaceae;g__Domibacillus;s__
RH52_bin.014	84.76	0	0	74	2205529	140	57575	2064	93.16	70.94	30	0	0	0	0	0	83.30	d__Bacteria;p__Actinobacteriota;c__Actinomycetia;o__Actinomycetales;f__Microbacteriaceae;g__Microbacterium;s__
RH52_bin.016	71.88	2.74	100	575	1956850	157	3755	2298	92.56	72.69	31	0	0	0	0	0	84.61	d__Bacteria;p__Actinobacteriota;c__Actinomycetia;o__Actinomycetales;f__Microbacteriaceae;g__Microbacterium;s__
RH52_bin.019	88.9	1.21	50	588	3165493	97	6485	3427	93.07	72.04	31	1	0	0	1	0	81.36	d__Bacteria;p__Actinobacteriota;c__Actinomycetia;o__Propionibacteriales;f__Nocardioidaceae;g__Nocardioides;s__
RH52_bin.022	62.5	5.36	25	607	7773149	40	28356	8001	80.27	36.06	61	8	0	0	17	0	86.85	d__Bacteria;p__Firmicutes;c__Bacilli;o__Bacillales;f__Bacillaceae;g__Bacillus_AY;s__
RH52_bin.025	56.14	0	0	374	3506575	88	10325	3674	89.21	48.01	40	0	0	0	2	0	95.62	d__Bacteria;p__Firmicutes;c__Bacilli;o__Brevibacillales;f__Brevibacillaceae;g__Brevibacillus;s__Brevibacillus formosus
RH52_bin.032	68.97	0	0	313	3867924	80	14871	3896	83.14	43.21	50	2	0	0	14	0	97.66	d__Bacteria;p__Firmicutes;c__Bacilli;o__Bacillales;f__Bacillaceae_A;g__Bacillus_AZ;s__Bacillus_AZ foraminis
RH52_bin.036	94.3	0.2	100	247	5803410	53	32287	5250	88.16	72.44	61	1	0	0	20	0	94.27	d__Bacteria;p__Actinobacteriota;c__Actinomycetia;o__Streptomycetales;f__Streptomycetaceae;g__Streptomyces;s__
RH52_bin.041	87.89	0.78	100	368	3225108	96	12989	3136	92.95	74.00	39	0	0	0	1	0	87.29	d__Bacteria;p__Actinobacteriota;c__Actinomycetia;o__Actinomycetales;f__Cellulomonadaceae;g__Isoptericola_B;s__
RH52_bin.043	75.44	0	0	164	3792305	81	37031	3733	83.16	74.00	18	3	0	0	9	0	N/A	d__Bacteria;p__Firmicutes;c__Bacilli;o__Bacillales;f__Planococcaceae;g__Lysinibacillus_B;s__
RH52_bin.044	68.42	1.72	100	328	6811752	45	32857	6211	86.98	72.67	56	2	0	0	20	0	92.59	d__Bacteria;p__Actinobacteriota;c__Actinomycetia;o__Streptomycetales;f__Streptomycetaceae;g__Streptomyces;s__
RH52_bin.045	81.03	0	0	200	4324792	71	33416	4293	86.91	63.52	41	1	0	0	7	0	86.94	d__Bacteria;p__Proteobacteria;c__Alphaproteobacteria;o__Rhizobiales;f__Beijerinckiaceae;g__Microvirga;s__
RH52_bin.046	65.52	5.17	66.67	874	2791714	110	3441	3308	93.05	72.36	24	0	0	0	1	0	80.10	d__Bacteria;p__Actinobacteriota;c__Actinomycetia;o__Propionibacteriales;f__Nocardioidaceae;g__Nocardioides;s__
RH52_bin.048	80.36	1.79	0	115	4598803	67	76761	4493	82.16	35.27	62	7	0	0	7	0	94.82	d__Bacteria;p__Firmicutes;c__Bacilli;o__Bacillales;f__Bacillaceae;g__Bacillus_AY;s__
RH52_bin.054	53.02	3.45	66.67	751	7323165	42	16569	6756	88.22	72.59	65	2	0	0	44	1	98.51	d__Bacteria;p__Actinobacteriota;c__Actinomycetia;o__Streptomycetales;f__Streptomycetaceae;g__Streptomyces;s__Streptomyces calvus
RH52_bin.057	78.57	0	0	39	4259100	72	286611	4162	86.30	37.69	65	8	0	0	44	0	95.16	d__Bacteria;p__Firmicutes;c__Bacilli;o__Bacillales;f__Bacillaceae_L;g__Bacillus_Q;s__Bacillus_Q onubensis_A
RH52_bin.058	81.03	0	0	20	3588415	86	343986	3620	88.87	41.72	50	2	0	0	7	0	95.42	d__Bacteria;p__Firmicutes;c__Bacilli;o__Bacillales;f__Bacillaceae;g__Bacillus;s__Bacillus pumilus
RH52_bin.063	84.7	2.94	44.44	334	5084755	61	22722	4732	90.41	61.09	21	3	0	0	1	0	84.83	d__Bacteria;p__Firmicutes;c__Bacilli;o__Paenibacillales;f__Paenibacillaceae;g__Paenibacillus;s__
RH52_bin.068	55.85	0	0	298	2931122	105	15527	2836	92.70	74.62	36	0	0	0	1	0	86.48	d__Bacteria;p__Actinobacteriota;c__Actinomycetia;o__Actinomycetales;f__Cellulomonadaceae;g__Isoptericola_B;s__
RH59_bin.004	97.44	4.97	22.22	343	3893446	66	17791	4136	92.17	69.70	38	0	0	0	9	0	85.24	d__Bacteria;p__Proteobacteria;c__Alphaproteobacteria;o__Caulobacterales;f__Caulobacteraceae;g__Phenylobacterium;s__
RH59_bin.005	99.6	0	0	113	4031281	64	55836	3684	91.16	75.64	45	0	0	0	1	0	98.74	d__Bacteria;p__Actinobacteriota;c__Actinomycetia;o__Actinomycetales;f__Cellulomonadaceae;g__Cellulomonas;s__Cellulomonas telluris
RH59_bin.009	94.2	1	25	93	4560140	56	81189	4437	86.55	63.35	42	0	0	0	7	0	86.87	d__Bacteria;p__Proteobacteria;c__Alphaproteobacteria;o__Rhizobiales;f__Beijerinckiaceae;g__Microvirga;s__
RH59_bin.011	72.76	3.45	100	122	5113483	50	74322	4477	88.88	67.78	45	1	0	0	5	1	92.38	d__Bacteria;p__Proteobacteria;c__Gammaproteobacteria;o__Burkholderiales;f__Burkholderiaceae;g__Cupriavidus;s__
RH59_bin.012	58.48	0	0	272	2747290	93	14757	2708	92.79	74.57	35	0	0	0	1	0	86.58	d__Bacteria;p__Actinobacteriota;c__Actinomycetia;o__Actinomycetales;f__Cellulomonadaceae;g__Isoptericola_B;s__
RH59_bin.014	81.03	0	0	18	3060189	84	299814	3078	87.32	44.95	61	7	0	0	18	0	85.28	d__Bacteria;p__Firmicutes;c__Bacilli;o__Bacillales;f__Domibacillaceae;g__Domibacillus;s__
RH59_bin.016	84.85	1.72	100	452	5201514	49	14184	4992	86.86	49.83	39	0	0	0	3	0	95.35	d__Bacteria;p__Firmicutes;c__Bacilli;o__Paenibacillales;f__Paenibacillaceae;g__Paenibacillus;s__Paenibacillus sp001860525
RH59_bin.019	61.4	0	0	178	4003889	64	37486	3918	88.22	47.70	59	0	0	0	3	0	95.44	d__Bacteria;p__Firmicutes;c__Bacilli;o__Brevibacillales;f__Brevibacillaceae;g__Brevibacillus;s__Brevibacillus formosus
RH59_bin.020	98.51	2.54	0	64	4370912	59	127384	4242	89.31	64.44	52	1	1	1	15	0	N/A	d__Bacteria;p__Proteobacteria;c__Alphaproteobacteria;o__Rhizobiales;f__Rhizobiaceae;g__Chelativorans;s__
RH59_bin.022	99.49	1.18	0	17	3226898	79	326149	2977	92.58	70.08	47	0	0	0	0	1	82.75	d__Bacteria;p__Actinobacteriota;c__Actinomycetia;o__Actinomycetales;f__Microbacteriaceae;g__Microbacterium;s__
RH59_bin.025	95.56	1.96	50	154	3672269	70	45435	3552	88.17	64.67	40	0	0	0	8	0	98.37	d__Bacteria;p__Proteobacteria;c__Alphaproteobacteria;o__Rhizobiales;f__Beijerinckiaceae;g__Microvirga;s__Microvirga pakistanensis
RH59_bin.028	99.51	2.58	40	125	9455374	27	125502	8764	89.04	71.32	66	2	0	0	21	0	89.88	d__Bacteria;p__Actinobacteriota;c__Actinomycetia;o__Mycobacteriales;f__Pseudonocardiaceae;g__Saccharothrix;s__
RH59_bin.029	96.16	2.15	7.69	119	4582535	56	71438	4462	82.08	35.29	58	9	0	0	6	0	94.89	d__Bacteria;p__Firmicutes;c__Bacilli;o__Bacillales;f__Bacillaceae;g__Bacillus_AY;s__
RH59_bin.030	74.14	1.72	0	31	4333349	59	310706	4010	86.37	55.26	63	3	0	0	4	0	92.23	d__Bacteria;p__Firmicutes;c__Bacilli;o__Paenibacillales;f__Paenibacillaceae;g__Paenibacillus_G;s__
RH59_bin.031	77.58	3.08	62.5	1206	4736193	54	4434	5369	89.32	70.10	33	0	0	0	3	0	N/A	d__Bacteria;p__Proteobacteria;c__Alphaproteobacteria;o__Acetobacterales;f__Acetobacteraceae;g__;s__
RH59_bin.032	99.5	1.37	16.67	36	5694357	45	370051	5429	90.33	69.68	46	0	0	0	5	0	81.61	d__Bacteria;p__Proteobacteria;c__Alphaproteobacteria;o__Acetobacterales;f__Acetobacteraceae;g__Roseomonas_A;s__
RH59_bin.035	75.82	2.59	100	94	5474079	47	119459	4756	88.31	67.67	52	3	0	0	6	2	92.39	d__Bacteria;p__Proteobacteria;c__Gammaproteobacteria;o__Burkholderiales;f__Burkholderiaceae;g__Cupriavidus;s__
RH59_bin.036	91.4	0.39	100	356	3246806	79	14999	3191	92.63	73.91	42	1	0	0	1	0	87.44	d__Bacteria;p__Actinobacteriota;c__Actinomycetia;o__Actinomycetales;f__Cellulomonadaceae;g__Isoptericola_B;s__
RH59_bin.043	61.65	0	0	1170	4724990	54	4788	5414	89.31	69.82	36	0	0	0	5	1	93.89	d__Bacteria;p__Actinobacteriota;c__Actinomycetia;o__Mycobacteriales;f__Pseudonocardiaceae;g__Saccharomonospora;s__
RH59_bin.044	96.3	1.2	100	193	3816350	67	27981	3948	88.38	46.16	34	4	0	0	28	0	96.78	d__Bacteria;p__Firmicutes;c__Bacilli;o__Bacillales;f__Bacillaceae;g__Bacillus;s__Bacillus haynesii
RH59_bin.045	83.9	7.35	61.54	455	7796851	33	27262	7459	88.79	72.24	57	1	0	0	4	1	90.69	d__Bacteria;p__Actinobacteriota;c__Actinomycetia;o__Mycobacteriales;f__Pseudonocardiaceae;g__Saccharothrix;s__
RH59_bin.048	94.14	5.76	35.71	438	5158805	50	16526	5218	81.51	35.51	47	6	0	0	12	0	81.66	d__Bacteria;p__Firmicutes;c__Bacilli;o__Bacillales;f__Bacillaceae;g__Bacillus_AY;s__
RH59_bin.051	61.21	1.21	100	547	1764443	145	3521	2221	92.31	71.07	24	0	0	0	2	0	85.37	d__Bacteria;p__Proteobacteria;c__Alphaproteobacteria;o__Caulobacterales;f__Caulobacteraceae;g__Brevundimonas;s__
RH59_bin.052	91.79	1.91	0	196	4129399	62	30077	4202	85.15	41.72	56	4	0	0	43	0	99.44	d__Bacteria;p__Firmicutes;c__Bacilli;o__Bacillales;f__Bacillaceae_A;g__Cytobacillus;s__s__Cytobacillus oceanisediminis_B
RH59_bin.053	79.7	8.67	45.71	1635	6775119	38	4905	7075	88.38	72.79	52	1	0	0	31	0	93.35	d__Bacteria;p__Actinobacteriota;c__Actinomycetia;o__Streptomycetales;f__Streptomycetaceae;g__Streptomyces;s__

Note: The high-quality MAGs (completeness >90% and contamination <5%) are displayed with a green background.

Supplementary Table 7 Summary statistics of BGCs for 33 high-quality MAGs predicted by antiSMASH.

MAG ID	Region		Type	From	To	Most similar known cluster		Similarity	Length
BS_bin.015	Region 2.1		NRPS	1	33,412				33,411
	Region 8.1		betalactone	36,938	67,033	auroramycin	Polyketide	2%	30,095
	Region 9.1		betalactone	2,089	43,615				41,526
RL56_bin.009	Region 2.1		RRE-containing	282,466	302,852	SF2575	Polyketide:Type II + Saccharide:Hybrid/tailoring	4%	20,386
	Region 4.1		siderophore	218,269	230,818	desferrioxamine	Other	50%	12,549
	Region 6.1		T3PKS	88,442	129,836				41,394
	Region 10.1		betalactone	36,203	63,126	microansamycin	Polyketide	7%	26,923
RL56_bin.023	Region 4.1		NAPAA	1,600	35,475				33,875
	Region 6.1		betalactone	1	27,672	rimosamide	NRP	14%	27,671
	Region 26.1		terpene	1,288	22,202	carotenoid	Terpene	37%	20,914
	Region 38.1		T3PKS	1	20,902				20,901
	Region 46.1		NAPAA	1	20,779				20,778
	Region 55.1		redox-cofactor	2,742	17,076	lankacidin C	NRP + Polyketide	13%	14,334
	Region 140.1		NRPS-like	1	5,728				5,727
RL56_bin.044	Region 2.1		betalactone	146,272	172,244	microansamycin	Polyketide	7%	25,972
	Region 3.1		siderophore	97,950	109,815	desferrioxamine E	Other	100%	11,865
	Region 5.1		NRPS-like,butyrolactone	74,066	116,705				42,639
	Region 41.1		T3PKS	1	26,921				26,920
RL58_bin.052	Region 1.1		terpene,NAPAA	38,993	94,028	carotenoid	Terpene	33%	55,035
	Region 21.1		betalactone	1	17,849	microansamycin	Polyketide	7%	17,848
	Region 25.1		T3PKS	2,660	29,317				26,657
	Region 45.1		NRPS-like	1	21,260				21,259
	Region 60.1		redox-cofactor	2,742	17,076	lankacidin C	NRP + Polyketide	13%	14,334
	Region 73.1		NAPAA	1	14,724				14,723
RM44_bin.004	Region 12.1		betalactone,T3PKS	41,676	87,360	microansamycin	Polyketide	7%	45,684
	Region 19.1		terpene	38,460	59,377	carotenoid	Terpene	28%	20,917
RM44_bin.005	Region 11.1		betalactone,T3PKS	106,363	142,587	microansamycin	Polyketide	7%	36,224
RM44_bin.009	Region 12.1		betalactone	1,164	26,946	microansamycin	Polyketide	7%	25,782
	Region 14.1		terpene	26,809	38,666	carotenoid	Terpene	50%	11,857
	Region 71.1		T3PKS	1	11,041				11,040
RM44_bin.014	Region 3.1		RiPP-like	246,261	257,085				10,824
	Region 5.1		ectoine	72,326	82,730	ectoine	Other	75%	10,404
	Region 8.1		siderophore	138,938	151,571				12,633
	Region 10.1		T3PKS	38,221	79,354	hygromycin A	Saccharide	6%	41,133
	Region 22.1		NRPS-like	1	31,042				31,041
RM44_bin.016	Region 41.1		T3PKS	1	23,297	hygromycin A	Saccharide	6%	23,296
	Region 46.1		siderophore	6,844	19,366	desferrioxamine	Other	33%	12,522
	Region 61.1		ectoine	1,436	11,840	ectoine	Other	75%	10,404
	Region 100.1		NRPS-like	1	12,596				12,595
RM44_bin.034	Region 7.1		T1PKS	1	33,919				33,918
	Region 12.1		hserlactone	52,469	73,113				20,644
	Region 16.1		thioamitides	47,113	69,132				22,019
	Region 30.1		hserlactone	1	13,290				13,289
	Region 46.1		redox-cofactor	11,707	33,894	lankacidin C	NRP + Polyketide	13%	22,187
	Region 57.1		terpene	16,497	27,348				10,851
	Region 79.1		RiPP-like	1,831	12,862				11,031
RM46_bin.030	Region 2.1		RRE-containing	335,804	356,091				20,287
	Region 5.1		betalactone,T3PKS	246,885	292,325	microansamycin	Polyketide	7%	45,440
	Region 7.1		terpene	55,752	76,621	carotenoid	Terpene	50%	20,869
RM46_bin.044	Region 2.1		T3PKS	56,813	97,895	salecan	Saccharide	10%	41,082
	Region 37.1		terpene	5,944	22,959				17,015
RM56_bin.013	Region 1.1		T3PKS	476,546	517,940				41,394
	Region 3.1		RRE-containing	180,454	200,840	SF2575	Polyketide:Type II + Saccharide:Hybrid/tailoring	4%	20,386
	Region 6.1		siderophore	46,289	58,838	desferrioxamine	Other	50%	12,549
	Region 9.1		betalactone	35,957	62,880	microansamycin	Polyketide	7%	26,923
RM56_bin.042	Region 1.1		thioamitides	130,161	152,180				22,019
	Region 11.1		T1PKS	1	36,140				36,139
	Region 22.1		hserlactone	40,962	61,585				20,623
	Region 75.1		hserlactone	13,507	25,458				11,951
	Region 76.1		redox-cofactor	1	20,574	lankacidin C	NRP + Polyketide	13%	20,573
	Region 102.1		RiPP-like	800	11,831				11,031
RM56_bin.049	Region 11.1		terpene	57,483	68,667				11,184
	Region 33.1		terpene	1	13,571	zeaxanthin	Terpene	66%	13,570
RM56_bin.052	Region 19.1		T3PKS	1	23,122				23,121
	Region 52.1		RRE-containing	1	15,582				15,581
	Region 66.1		betalactone	1	12,813	microansamycin	Polyketide	7%	12,812
RM56_bin.064	Region 1.1		terpene	176,465	197,331	carotenoid	Terpene	66%	20,866
	Region 1.2		T3PKS	262,466	303,521	alkylresorcinol	Polyketide	100%	41,055
	Region 7.1		betalactone	82,380	109,522	microansamycin	Polyketide	7%	27,142
RH52_bin.003	Region 5.1		T3PKS	112,552	153,634				41,082
	Region 8.1		betalactone	117,345	141,564	fengycin	NRP	46%	24,219
	Region 12.1		NRPS-like	44,720	84,454	kijanimicin	Polyketide	4%	39,734
RH52_bin.008	Region 1.1		T3PKS	281,879	322,961	arsono-polyketide	Polyketide	8%	41,082
	Region 1.2		ectoine	359,488	369,871	ectoine	Other	75%	10,383
	Region 6.1		cyclic-lactone-autoinducer	246,534	267,058				20,524
	Region 16.1		proteusin	7,771	28,016				20,245
	Region 17.1		lassopeptide	40,768	64,645	paeninodin	RiPP	80%	23,877
	Region 41.1		cyclic-lactone-autoinducer	338	20,892				20,554
RH52_bin.036	Region 1.1		terpene	12,078	38,711	hopene	Terpene	92%	26,633
	Region 2.1		terpene	7,613	29,472	SCO-2138	RiPP	21%	21,859
	Region 3.1		ectoine	19,724	30,122	ectoine	Other	100%	10,398
	Region 8.1		siderophore	63,614	75,386	desferrioxamin B / desferrioxamine E	Other	83%	11,772
	Region 20.1		NRPS	1	42,769	amychelin	NRP	62%	42,768
	Region 21.1		terpene	40,258	54,654	geosmin	Terpene	100%	14,396
	Region 28.1		RiPP-like	26,651	38,573	sisomicin	Saccharide	5%	11,922
	Region 33.1		NRPS-like,T1PKS,lipolanthine,lanthipeptide-class-iii	1	32,435	cylindrospermopsin	NRP + Polyketide	66%	32,434
	Region 38.1		T3PKS	1	26,527	zorbamycin	NRP:Glycopeptide + Polyketide:Modular type I + Saccharide:Hybrid/tailoring	8%	26,526
	Region 41.1		NRPS,other	1	37,290	polyoxypeptin	NRP + Polyketide	54%	37,289
	Region 44.1		T1PKS,NRPS,other	1	36,552	polyoxypeptin	NRP + Polyketide	18%	36,551
	Region 56.1		siderophore	10,199	23,325	lividomycin	Saccharide	10%	13,126
	Region 64.1		siderophore	15,361	27,343	ficellomycin	NRP	3%	11,982
	Region 68.1		furan,butyrolactone	7,875	28,891	methylenomycin A	Other	23%	21,016
	Region 78.1		NRPS,T1PKS	1	27,032				27,031
	Region 79.1		T3PKS	1	27,026	herboxidiene	Polyketide	7%	27,025
	Region 84.1		terpene	1	12,278	triacsins	Other	6%	12,277
	Region 107.1		lassopeptide	1	18,510	citrulassin D	RiPP	80%	18,509
	Region 171.1		terpene	1	10,765	albaflavenone	Terpene	100%	10,764
	Region 180.1		melanin	2,396	9,653	istamycin	Saccharide	5%	7,257
	Region 190.1		butyrolactone	1	8,255	griseoviridin / fijimycin A	NRP:Cyclic depsipeptide +	8%	8,254

							Polyketide:Trans-AT type I		
	Region 220.1		terpene	1	5,690	isorenieratene	Terpene	71%	5,689
	Region 221.1		RiPP-like	1	5,671	informatipeptin	RiPP:Lanthipeptide	28%	5,670
RH59_bin.004	Region 191.1		T3PKS	1	6,701				6,700
RH59_bin.005	Region 1.1		siderophore	38,044	49,969	desferrioxamin B / desferrioxamine E	Other	66%	11,925
	Region 2.1		lanthipeptide-class-iii	57,160	79,817	formicamycins A-M	Polyketide	4%	22,657
	Region 10.1		amglyccycl	8,198	29,385	acarbose	Saccharide	17%	21,187
	Region 14.1		T3PKS	1	39,351	alkylresorcinol	Polyketide	100%	39,350
	Region 49.1		NAPAA	4,584	30,323				25,739
	Region 63.1		terpene	1	20,387	carotenoid	Terpene	50%	20,386
RH59_bin.009	Region 6.1		NRPS-like	92,641	135,622	lasalocid	Polyketide	5%	42,981
	Region 7.1		NRPS-like	101,193	135,419				34,226
	Region 21.1		terpene	15,764	37,422				21,658
	Region 31.1		terpene	33,606	54,448				20,842
	Region 44.1		hserlactone	1	17,927				17,926
RH59_bin.020	Region 19.1		terpene	30,282	51,127				20,845
	Region 26.1		hserlactone	27,945	48,586				20,641
	Region 28.1		hserlactone	20,216	40,857				20,641
RH59_bin.022	Region 3.1		T3PKS,betalactone	81,003	126,330	microansamycin	Polyketide	7%	45,327
RH59_bin.025	Region 18.1		terpene	20,584	41,426				20,842
	Region 39.1		hserlactone	8,207	28,839				20,632
	Region 59.1		terpene	10,778	22,724				11,946
RH59_bin.028	Region 2.1		NRPS,lanthipeptide-class-i,lanthipeptide-class-ii	62,238	120,209	daptomycin	NRP	3%	57,971
	Region 2.2		NRPS	245,356	311,545	ishigamide	NRP + Polyketide	11%	66,189
	Region 3.1		T1PKS,NRPS,NRPS-like	21,159	72,308	matlystatin A	NRP + Polyketide	11%	51,149
	Region 4.1		terpene	27,169	47,904	actagardine	RiPP:Lanthipeptide	9%	20,735
	Region 4.2		terpene,NRPS	124,064	169,439	brasilicardin A	Terpene + Saccharide	38%	45,375
	Region 4.3		blactam,RiPP-like,thiopeptide,LAP	199,894	262,325	valclavam / (-)-2-(2-hydroxyethyl)clavam	Other:Non-NRP beta-lactam	64%	62,431
	Region 5.1		arylpolyene	117,443	158,609				41,166
	Region 7.1		NRPS	35,292	74,816	kedarcidin	Polyketide:Iterative type I + Polyketide:Enediyne type I	6%	39,524
	Region 7.2		T1PKS	97,142	156,880	lavendiol	Polyketide	25%	59,738
	Region 7.3		NRPS	190,820	234,014	azicemicin B	Polyketide	11%	43,194
	Region 10.1		RRE-containing	1	16,691				16,690
	Region 10.2		T1PKS	89,750	157,197	argimycin PI / argimycin PII / nigrifactin / argimycin PIV / argimycin PV / argimycin PVI / argimycin PIX	Polyketide:Modular type I	21%	67,447
	Region 11.1		NRPS-like,lassopeptide	59,612	101,315	rimosamide	NRP	14%	41,703
	Region 12.1		terpene	24,323	50,997	isorenieratene	Terpene	42%	26,674
	Region 12.2		lanthipeptide-class-iii	115,018	137,555	Ery-9 / Ery-6 / Ery-8 / Ery-7 / Ery-5 / Ery-4 / Ery-3	RiPP:Lanthipeptide	75%	22,537
	Region 18.1		T1PKS,NRPS	1	67,801	selvamicin	Polyketide	25%	67,800
	Region 21.1		terpene	99,090	118,061	2-methylisoborneol	Terpene	50%	18,971
	Region 22.1		thiopeptide	25,698	53,896	capreomycin IA / capreomycin IB / capreomycin IIA / capreomycin IIB	NRP	12%	28,198
	Region 23.1		lanthipeptide-class-iv	1	20,862	meilingmycin	Polyketide	2%	20,861
	Region 23.2		terpene	72,313	94,523	geosmin	Terpene	100%	22,210
	Region 30.1		NRPS-like	56,932	98,098	livipeptin	NRP	100%	41,166
	Region 33.1		lanthipeptide-class-i	46,197	70,673	enduracidin	NRP	4%	24,476
	Region 36.1		NRPS-like,T1PKS,NRPS	3	53,742	enduracidin	NRP	8%	53,739
	Region 37.1		indole	22,319	43,419	fortimicin	Saccharide	4%	21,100
	Region 39.1		redox-cofactor	32,836	54,845	lankacidin C	NRP + Polyketide	26%	22,009
	Region 40.1		NRPS-like	21,396	63,789	daptomycin	NRP	4%	42,393
	Region 41.1		RiPP-like	28,295	39,092				10,797
	Region 43.1		hglE-KS	1	36,362				36,361
	Region 43.2		lanthipeptide-class-ii	46,178	68,656				22,478
	Region 45.1		NRPS,T1PKS	1	66,630	amychelin	NRP	68%	66,629
	Region 47.1		amglyccycl	28,390	49,466	cetoniacytone A	Other:Cyclitol	9%	21,076
	Region 57.1		T1PKS,NRPS-like,NRPS,LAP	1	54,040	catenulisporolides	NRP + Polyketide	16%	54,039
	Region 58.1		terpene	41,702	54,006	SF2575	Polyketide:Type II + Saccharide:Hybrid/tailoring	6%	12,304
	Region 62.1		lassopeptide	454	22,828	citrulassin F	RiPP	100%	22,374
	Region 74.1		T1PKS	1	27,607	primycin	Polyketide	5%	27,606
RH59_bin.029	Region 4.1		T3PKS	1	23,656				23,655
	Region 4.2		terpene	66,353	88,227				21,874
	Region 4.3		NRPS	144,050	171,043				26,993
	Region 7.1		ladderane	101	41,297				41,196
	Region 17.1		T3PKS	1	33,810				33,809
	Region 31.1		lassopeptide	1	16,058	paeninodin	RiPP	60%	16,057
RH59_bin.032	Region 1.1		terpene	695,504	716,310				20,806
	Region 2.1		T3PKS	1	37,279				37,278
	Region 4.1		arylpolyene	331,311	372,513	APE Vf	Other	10%	41,202
	Region 4.2		betalactone	408,231	437,270	oxalomycin B	NRP + Polyketide	6%	29,039
	Region 5.1		T1PKS	261,701	308,936				47,235
	Region 10.1		betalactone	47,451	69,984				22,533
	Region 13.1		redox-cofactor	25,504	47,613	lankacidin C	NRP + Polyketide	13%	22,109
	Region 13.2		terpene	143,260	164,852				21,592
RH59_bin.036	Region 51.1		siderophore	2,661	15,156	desferrioxamin B	Other	60%	12,495
	Region 64.1		T3PKS	1	14,516	alkylresorcinol	Polyketide	100%	14,515
	Region 90.1		NAPAA	1	11,204				11,203
	Region 317.1		RRE-containing	1	2,031				2,030
RH59_bin.044	Region 7.1		siderophore	24,786	40,252				15,466
	Region 20.1		CDPS	5,688	26,437				20,749
	Region 30.1		betalactone	4,375	32,890	fengycin	NRP	53%	28,515
	Region 31.1		RiPP-like	9,667	20,011				10,344
	Region 34.1		NRPS	1	26,444	lichenysin	NRP	50%	26,443
	Region 37.1		T3PKS	1	23,621				23,620
	Region 44.1		lanthipeptide-class-ii	10,722	28,703	geobacillin II	RiPP:Lanthipeptide	50%	17,981
	Region 114.1		RiPP-like	1	8,848				8,847
	Region 116.1		NRPS	1	12,124	lichenysin	NRP	35%	12,123
	Region 132.1		NRPS	1	9,835	lichenysin	NRP	14%	9,834
	Region 137.1		terpene	1	8,998				8,997
RH59_bin.052	Region 20.1		siderophore	4,159	17,663	petrobactin	Other	33%	13,504
	Region 57.1		T3PKS	1	25356				25,355
	Region 84.1		terpene	756	16611				15,855
	Region 106.1		LAP,RiPP-like	1	14032				14,031

Supplementary Table 8 Media composition used in the present study.

No.	Culture media (Abbreviation)	Source	Target bacterial groups	Composition (Per liter)
M1	Bennet's agar (BEN)	SPL[a]	*Nocardia*	Glucose 10.0g, Casein acid hydrolysate 2.0g, Beef extract 1.0g, Yeast extract 0.5g, pH 7.2±0.1.
M2	Brain heart infusion agar (BHI)	Coolaber[b]	Fastidious bacteria	Calf brains 12.5g, Peptone 10.0g, Beef heart 5.0g, NaCl 5.0g, Na_2HPO_4 2.5g, Glucose 2.0g, pH 7.2±0.1.
M3	Brooks agar (BRA)	SPL	Actinomycetes	Cornmeal, 5.0g, Yeast extract 2.0g, KH2PO4 1.5g, Malt extract 1.0g, Tryptone 1.0g, $MgSO_4·7H_2O$ 0.5g, pH 7.2±0.1.
M4	Casein salt agar (CSA)	SPL	Halophilic bacteria	NaCl 100.0g, Sea salt 10.0g, Casein acid hydrolysate 2.0g, KCl 2.0g, $MgSO_4·7H_2O$ 2.0g, Yeast extract 1.0g, Sodium glutamate 1.0g, Trisodium citrate 1.0g, pH 7.2±0.1.
M5	Chitin agar (CHIA)	SPL	Chitin-degrading bacteria	Chitin 4.0g, K_2HPO_4 0.7g, $MgSO_4·7H_2O$ 0.5g, KH_2PO_4 0.5g, pH 8.0±0.1.
M6	Czapek's agar (CZA)	SPL	Actinomycetes	Sucrose 30.0g, $NaNO_3$ 2.0g, K_2HPO_4 1.0g, $MgSO_4·7H_2O$ 0.5g, KCl 0.5g, pH 7.2±0.1.
M7	Gauze's no. 1 agar (GN1)	SPL	Actinomycetes	Soluble starch 20.0g, KNO_3 1.0g, NaCl 0.5g, K_2HPO_4 0.5g, $MgSO_4·7H_2O$ 0.5g, pH 7.2±0.1.
M8	Glucose yeast malt agar (GYM)	SPL	Actinomycetes (*Streptomyces*)	Malt extract 10.0g, Yeast extract 4.0g, Glucose 4.0g, $CaCO_3$ 2.0g, pH 7.2±0.1.
M9	Glycerol agar (GLA)	SPL	Fastidious bacteria	Glycerol 60.0g, Proteose peptone 7.0g, NaCl 3.0g, Peptone 2.5g, Yeast extract 1.0g, Beef extract 0.5g, pH 7.2±0.1.
M10	Glycerol glycine agar (GGA)	SPL	*Streptomyces*	Glycerol 20.0g, Glycine 2.5g, NaCl 1.0g, K_2HPO_4 1.0g, $CaCO_3$ 0.1g, $FeSO_4·7H_2O$ 0.02g, $MgSO_4·7H_2O$ 0.01g, pH 7.2±0.1.
M11	Halophilic agar (HA)	SPL	Halophilic bacteria	NaCl 100.0g, Sea salt 10.0g, $MgSO_4·7H_2O$ 10.0g, Casamino acids 3.0g, Yeast extract 3.0g, Proteose peptone 3.0g, Trisodium citrate 2.5g, KCl 2.0g, Sodium glutamate 1.0g, pH 7.2±0.1.
M12	Humic acid-vitamin agar (HVA)	SPL	Actinomycetes	Humic acid 0.1g, Na_2HPO_4 0.5g, KCl 1.71g, $MgSO_4·7H_2O$ 0.05g, $CaCO_3$ 0.02g, B-vitamins 1mL, pH 7.2±0.1.
M13	King's B agar (KB)	SPL	*Pseudomonas*	Proteose peptone 20.0g, K_2HPO_4 1.5g, $MgSO_4·7H_2O$ 1.5g, Glycerol 10.0g, pH 7.2±0.1.
M14	Low nutrient mineral salts agar (LNMS)	SPL	Oligotrophic bacteria	K_2HPO_4 2.0g, NaCl 0.5g, Soluble starch 0.1g, Yeast extract 0.1g, $MgSO_4·7H_2O$ 0.5g, $CaCO_3$ 0.02g, pH 7.2±0.1.
M15	Luria-Bertani agar (LB)	SPL	Heterotrophic bacteria	Tryptone 10.0g, Yeast extract 5.0g, NaCl 10.0g, pH 7.2±0.1.
M16	Mannitol casein agar (MCA)	SPL	Actinomycetes	$(NH_4)_2SO_4$ 2.0g, Mannitol 1.8g, KNO_3 1.0g, N-Z-Case® Plus 0.5g, KH_2PO_4 0.5g, $MgSO_4·7H_2O$ 0.2g, $CaCO_3$ 0.5g, pH 7.2±0.1.
M17	Mannitol Yeast Peptone Agar (MYP)	SPL	Actinomycetes	Mannitol 10.0g, Yeast extract 2.0g, Soya peptone 2.0g, KH_2PO_4 0.5g, $MgSO_4·7H_2O$ 0.2g, $CaCO_3$ 1.0g, NaCl 1.0g, pH 7.2±0.1.
M18	Microcrystalline cellulose agar (MIC)	SPL	Cellulose-degrading bacteria	Microcrystalline cellulose 10.0g, K_2HPO_4 1.0g, $(NH_4)_2SO_4$ 0.5g, L-Asparagine 0.5g, KCl 0.5g, Yeast extract 0.5g, $MgSO_4·7H_2O$ 0.2g, $CaCl_2·2H_2O$ 0.1g, pH 7.2±0.1.
M19	Minimal Medium agar (MM)	SPL	Oligotrophic bacteria	Glucose 0.5g, Yeast extract 0.5g, K_2HPO_4 1.0g, $MgSO_4·7H_2O$ 0.5g, NaCl 0.5g, pH 7.2±0.1.
M20	Peptone yeast glucose agar (PYG)	SPL	Fastidious bacteria	Soya peptone 5.0g, Yeast extract 5.0g, Glucose 5.0g, pH 7.2±0.1.
M21	Peptone seast sodium succinate agar (PYS)	SPL	*Hymenobacter*	Soya peptone 3.0g, Yeast extract 3.0g, Sodium succinate 2.3g, pH 7.2±0.1.
M22	Polypeptone starch agar (PPS)	SPL	*Bacillus*	Polypeptone 10.0g, Soluble starch 5.0g, K_2HPO_4 3.0g, $MgSO_4·7H_2O$ 1.0g, pH 7.2±0.1.
M23	Potato dextrose agar (PDA)	Oxoid[c]	Heterotrophic bacteria	Glucose 20.0g, Potato infusion: Boil 300 g scrubbed and sliced potatoes in 1L water for 1 hour. Pass through fine sieve; pH 7.2±0.1.
M24	R agar (RA)	SPL	Actinomycetes	Peptone 10.0g, Casamino acids 5.0g, Malt extract 5.0g, Yeast extract 5.0g, Beef extract 2.0g, Glycerol 2.0g, $MgSO_4·7H_2O$ 1.0g, Tween™ 80 50.0mg, pH 7.2±0.1.
M25	Raffinose histidine agar (RH)	SPL	Actinomycetes	Raffinose 5.0g, Histidine 1.0g, KNO_3 1.0g, NaCl 1.0g, $CaCl_2$ 2.0g, K_2HPO_4 1.0g, $MgSO_4·7H_2O$ 1.0g, pH 7.2±0.1.
M26	Rhizobium 1 agar (RZ1)	SPL	*Rhizobiaceae*	Mannitol 10.0g, Yeast extract 1.0g, Sodium glutamate 0.5g, KH_2PO_4 0.5g, $MgSO_4·7H_2O$ 0.1g, $CaCl_2·2H_2O$ 0.04g, pH 7.2±0.1.
M27	Rhizobium 2 agar (RZ2)	SPL	*Rhizobiaceae*	Glycerol 4.6g, $CaSO_4$ 1.3g, Yeast extract 1.0g, KH_2PO_4 1.0g, L-Arabinose 1.0g, KNO_3 0.7g, $MgSO_4·7H_2O$ 0.36g, $CaCl_2·2H_2O$ 0.04g, $FeCl_3·6H_2O$ 0.004g, pH 7.2±0.1.
M28	Salt agar (SA)	SPL	Halophilic bacteria	NaCl 58.4g, Proteose peptone 5.0g, Tryptone 5.0g, pH 7.2±0.1.
M29	Salt sodium molybdate agar (SSM)	SPL	Halophilic bacteria	NaCl 100.0g, Sea salt 5.0g, Na_2MoO_4 5.0g, Soluble Starch 2.0g, Beef extract 1.0g, Yeast extract 1.0g, Casein acid hydrolysate 1.0g, $CaCO_3$ 1.0g, pH 7.2±0.1.
M30	Sea salt tryptone yeast agar (SSTY)	SPL	Halophilic bacteria	Sea salt 35.0g, Tryptone 5.0g, Yeast extract 5.0g, pH 7.2±0.1.
M31	Carboxymethyl Cellulose Agar (CMCA)	SPL	Cellulose-degrading bacteria	CMC-Na 5.0g, $(NH_4)_2SO_4$ 2.0g, K_2HPO_4 2.0g, NaCl 0.5g, $MgSO_4·7H_2O$ 0.5g, Yeast extract 0.4g, $CaCl_2$ 0.3g, pH 7.2±0.1.
M32	Sodium Lactate Agar (SLA)	SPL	*Propionibacterium*	Sodium lactate 10.0g, K_2HPO_4 1.67g, Yeast extract 1.0g, KH_2PO_4 0.87g, $MgSO_4·7H_2O$ 0.5g, NaCl 0.5g, $CaCl_2$ 0.2g, pH 7.2±0.1
M33	Soil extract agar (Bulk soil) (SEAB)	SPL	Oligotrophic bacteria	Glucose 1.0g, Yeast extract 1.0g, Peptone 1.0g, Beef extract 0.5g, K_2HPO_4 0.5g, Soil extract 1 L (400 g bulk soil+1 L H_2O), pH 7.2±0.1.
M34	Soil extract agar (Rhizosphere) (SEAR)	SPL	Oligotrophic bacteria	Glucose 1.0g, Yeast extract 1.0g, Peptone 1.0g, Beef extract 0.5g, K_2HPO_4 0.5g, Soil extract 1 L (400 g Rhizosphere soil+1 L H_2O), pH 7.2±0.1.
M35	Starch casein agar (SCA)	SPL	Actinomycetes	Soluble starch 10.0g, K_2HPO_4 2.0g, KNO_3 2.0g, NaCl 2.0g, Casein 0.3g, $MgSO_4·7H_2O$ 0.05g, $CaCO_3$ 0.02g, $FeSO_4·7H_2O$ 0.01g, pH 7.2±0.1.
M36	Starch glucose casein yeast agar (SGCY)	SPL	Actinomycetes (*Streptomyces*)	Soluble starch 20.0g, Glucose 5.0g, Casein acid hydrolysate 5.0g, Yeast extract 5.0g, CaCO3 1.0g, pH 7.2±0.1.
M37	Starch Nitrate Agar (SNA)	SPL	*Saccharomonospora*, *Microbispora*	NaCl 100.0g, Soluble starch 20.0g, $CaCO_3$ 3.0g, KNO_3 2.0g K_2HPO_4 1.0g, $MgSO_4·7H_2O$ 0.5g, pH 7.2±0.1.
M38	Starch Tryptone Agar (STA)	SPL	Actinomycetes	Soluble starch 5.0g, Tryptone 2.5g, $MgSO_4·7H_2O$ 0.5g, K_2HPO_4 0.25g, pH 7.2±0.1.
M39	Sucrose tryptone beef glucose agar (STBG)	SPL	*Saccharococcus*	Sucrose 5.0g, Tryptone 5.0g, Beef extract 3.0g, Glucose 1.0g, pH 7.2±0.1.
M40	Tang 5 agar (T5)	SPL	Actinomycetes	Yeast extract 2.0g, Glucose 1.0g, Lotus root starch 1.0g, Tryptone 0.5g, $CaCO_3$ 1.0g, pH 7.2±0.1.
M41	Trehalose proline agar (TP)	SPL	Actinomycetes	Trehalose 5.0g, Proline 3.0g, $CaCl_2$ 2.0g, $(NH_4)_2SO_4$ 1.0g, NaCl 1.0g, K_2HPO_4 1.0g, pH 7.2±0.1.
M42	Tryptic glucose yeast agar (TGY)	SPL	Heterotrophic bacteria	Tryptone 5.0g, Yeast extract 3.0g, Glucose 1.0g, K2HPO4 1.0g, pH 7.2±0.1.
M43	Tyrosine casein nitrate agar (TCN)	SPL	*Streptomyces*	Casein acid hydrolysate 12.5g, $NaNO_3$ 5.0g, L-Tyrosine 0.5g, pH 7.2±0.1.
M44	Yeast glucose carbonate peptone agar (YGCP)	SPL	Heterotrophic bacteria	$CaCO_3$ 10.0g, Yeast extract 2.5g, Glucose 2.5g, Peptone 2.5g, Triammonium citrate 2.0g, Sodium acetate 2.0g, NaCl 1.0g, K2HPO4 1.0g, MgSO4·7H2O 0.5g, pH 7.2±0.1.
M45	Yeast milk glucose agar (YMG)	SPL	*Lysobacter*	Skim milk powder 10.0g, Yeast extract 1.0g, Glucose 1.0g, pH 7.2±0.1.
M46	Tryptic soy agar (1×TSA)	BD[d]	Heterotrophic and fast-growing bacteria	Tryptone 17.0g, NaCl 5.0g, Soya peptone 3.0g, Glucose 2.5g, K_2HPO_4 2.5g, pH 7.2±0.1.
M47	1/10 Tryptic soy agar (0.1×TSA)	BD	Heterotrophic and oligotrophic bacteria	1/10 strength TSA
M48	1/100 Tryptic soy agar (0.01×TSA)	BD	Heterotrophic and oligotrophic bacteria	1/100 strength TSA
M49	Nutrient agar (1×NA)	Oxoid	Heterotrophic and fast-growing bacteria	Peptone 10.0g, Beef extract 3.0g, NaCl 5.0g, pH 7.2±0.1.
M50	1/10 Nutrient agar (0.1×NA)	Oxoid	Heterotrophic and oligotrophic bacteria	1/10 strength NA
M51	1/100 Nutrient agar (0.01×NA)	Oxoid	Heterotrophic and oligotrophic bacteria	1/100 strength NA
M52	Actinomycete isolation agar (1×AIA)	SPL	Actinomycetes	Sodium propionate 4.0g, Sodium caseinate 2.0g, K_2HPO_4 0.5g, L-Asparagine 0.1g, $MgSO_4·7H_2O$ 0.1g, pH 8.1±0.1.
M53	1/10 Actinomycete isolation agar (0.1×AIA)	SPL	Actinomycetes	1/10 strength AIA
M54	1/100 Actinomycete isolation agar (0.01×AIA)	SPL	Actinomycetes	1/100 strength AIA
M55	Reasoner's 2A agar (1×R2A)	BD	Heterotrophic and oligotrophic bacteria	Tryptone 0.5g, Yeast extract 0.5g, Casein acid hydrolysate 0.5g, Glucose 0.5g, Soluble starch 0.5g, K_2HPO_4 0.3g, Sodium pyruvate 0.3g, Peptic digest of animal tissue 0.25g, $MgSO_4$ anhydrous 0.024g, pH 7.2±0.1.
M56	1/10 Reasoner's 2A agar (0.1×R2A)	BD	Heterotrophic and oligotrophic bacteria	1/10 strength R2A
M57	1/100 Reasoner's 2A agar (0.01×R2A)	BD	Heterotrophic and oligotrophic bacteria	1/100 strength R2A
M58	Marine 2216E agar (1×MA)	BD	Heterotrophic bacteria	NaCl 19.45g, $MgCl_2$ 8.8g, Peptone 5.0g, Na_2SO_3 3.24g, $CaCl_2$ 1.8g, Yeast extract 1.0g, KCl 0.55g, $NaHCO_3$ 0.16g, Ferric citrate 0.1g, KBr 0.08g, $SrCl_2$ 0.03g, H_3BO_3 0.02g, Na_2HPO_4 8.0mg, Na_2SiO_3 4.0mg, NaF 2.4mg, NH_4NO_3 1.6mg, pH 7.2±0.1 (BD).
M59	1/10 Marine 2216E agar (0.1×MA)	BD	Heterotrophic and oligotrophic bacteria	1/10 strength MA
M60	1/100 Marine 2216E agar (0.01×MA)	BD	Heterotrophic and oligotrophic bacteria	1/100 strength MA

Note: All the media were added to 1.8% agar and autoclaved at 121 °C for 20 min. pH values were measured at 25 °C. [a] SPL, self-prepared in the laboratory; [b] Beijing, CN; [c] Basingstoke, UK; [d] Sparks, USA.

Supplementary Table 9 Incubation time for each culture-enriched sample.

Medium No.	Abbr.	Bulk soil						Rhizosphere soil					
		15 °C	Incubation time (d)	30 °C	Incubation time (d)	45 °C	Incubation time (d)	15 °C	Incubation time (d)	30 °C	Incubation time (d)	45 °C	Incubation time (d)
M1	BEN	BL1	8	BM1	3	BH1	6	RL1	9	RM1	3	RH1	3
M2	BHI	BL2	11	BM2	7	BH2	3	RL2	9	RM2	3	RH2	3
M3	BRA	BL3	6	BM3	3	BH3	3	RL3	7	RM3	3	RH3	3
M4	CSA	BL4	13	BM4	7	BH4	6	RL4	15	RM4	8	RH4	13
M5	CHIA	BL5	8	BM5	5	BH5	5	RL5	11	RM5	3	RH5	3
M6	CZA	BL6	6	BM6	3	BH6	3	RL6	8	RM6	3	RH6	3
M7	GN1	BL7	8	BM7	3	BH7	3	RL7	9	RM7	4	RH7	3
M8	GYM	BL8	7	BM8	3	BH8	6	RL8	10	RM8	3	RH8	4
M9	GLA	BL9	6	BM9	3	BH9	3	RL9	7	RM9	3	RH9	4
M10	GGA	BL10	10	BM10	7	BH10	8	RL10	10	RM10	4	RH10	9
M11	HA	BL11	8	BM11	3	BH11	6	RL11	13	RM11	4	RH11	13
M12	HVA	BL12	10	BM12	5	BH12	5	RL12	13	RM12	3	RH12	6
M13	KB	BL13	6	BM13	3	BH13	3	RL13	7	RM13	3	RH13	3
M14	LNMS	BL14	8	BM14	5	BH14	3	RL14	9	RM14	3	RH14	9
M15	LB	BL15	6	BM15	3	BH15	3	RL15	7	RM15	3	RH15	3
M16	MCA	BL16	10	BM16	3	BH16	6	RL16	13	RM16	3	RH16	6
M17	MYP	BL17	6	BM17	3	BH17	3	RL17	8	RM17	3	RH17	3
M18	MIC	BL18	9	BM18	5	BH18	5	RL18	10	RM18	3	RH18	6
M19	MM	BL19	6	BM19	5	BH19	3	RL19	8	RM19	3	RH19	3
M20	PYG	BL20	7	BM20	3	BH20	6	RL20	8	RM20	3	RH20	3
M21	PYS	BL21	6	BM21	3	BH21	5	RL21	7	RM21	3	RH21	3
M22	PPS	BL22	8	BM22	3	BH22	3	RL22	7	RM22	3	RH22	4
M23	PDA	BL23	7	BM23	3	BH23	6	RL23	8	RM23	3	RH23	3
M24	RA	BL24	6	BM24	3	BH24	3	RL24	7	RM24	3	RH24	3
M25	RH	BL25	6	BM25	3	BH25	5	RL25	10	RM25	3	RH25	6
M26	RZ1	BL26	13	BM26	5	BH26	5	RL26	13	RM26	3	RH26	6
M27	RZ2	BL27	14	BM27	8	BH27	6	RL27	15	RM27	4	RH27	9
M28	SA	BL28	7	BM28	5	BH28	3	RL28	10	RM28	4	RH28	6
M29	SSM	BL29	14	BM29	9	BH29	9	RL29	15	RM29	8	RH29	13
M30	SSTY	BL30	8	BM30	3	BH30	3	RL30	8	RM30	3	RH30	4
M31	CMCA	BL31	10	BM31	5	BH31	5	RL31	11	RM31	3	RH31	4
M32	SLA	BL32	7	BM32	3	BH32	3	RL32	9	RM32	3	RH32	3
M33	SEA-R	BL33	6	BM33	3	BH33	5	RL33	7	RM33	3	RH33	3
M34	SEA-B	BL34	7	BM34	3	BH34	5	RL34	7	RM34	3	RH34	4
M35	SCA	BL35	13	BM35	5	BH35	5	RL35	13	RM35	3	RH35	6
M36	SGCY	BL36	7	BM36	3	BH36	5	RL36	8	RM36	3	RH36	4
M37	SNA	BL37	14	BM37	13	BH37	12	RL37	15	RM37	12	RH37	15
M38	STA	BL38	6	BM38	3	BH38	3	RL38	7	RM38	3	RH38	4
M39	STBG	BL39	6	BM39	3	BH39	3	RL39	7	RM39	3	RH39	4
M40	T5	BL40	7	BM40	3	BH40	5	RL40	7	RM40	3	RH40	6
M41	TP	BL41	14	BM41	9	BH41	12	RL41	15	RM41	6	RH41	10
M42	TGY	BL42	7	BM42	3	BH42	3	RL42	8	RM42	3	RH42	3
M43	TCN	BL43	6	BM43	3	BH43	3	RL43	7	RM43	3	RH43	4
M44	YGCP	BL44	6	BM44	3	BH44	3	RL44	7	RM44	3	RH44	3
M45	YMG	BL45	8	BM45	3	BH45	6	RL45	10	RM45	3	RH45	4
M46	1×TSA	BL46	6	BM46	3	BH46	3	RL46	8	RM46	3	RH46	3
M47	0.1×TSA	BL47	7	BM47	3	BH47	3	RL47	8	RM47	3	RH47	3
M48	0.01×TSA	BL48	8	BM48	3	BH48	3	RL48	10	RM48	3	RH48	6
M49	1×NA	BL49	7	BM49	3	BH49	3	RL49	7	RM49	3	RH49	3
M50	0.1×NA	BL50	7	BM50	5	BH50	3	RL50	8	RM50	3	RH50	6
M51	0.01×NA	BL51	9	BM51	5	BH51	9	RL51	13	RM51	3	RH51	10
M52	1×AIA	BL52	7	BM52	3	BH52	3	RL52	8	RM52	3	RH52	3
M53	0.1×AIA	BL53	8	BM53	5	BH53	5	RL53	9	RM53	3	RH53	4
M54	0.01×AIA	BL54	11	BM54	5	BH54	9	RL54	13	RM54	3	RH54	9
M55	1×R2A	BL55	7	BM55	3	BH55	5	RL55	8	RM55	3	RH55	4
M56	0.1×R2A	BL56	7	BM56	3	BH56	5	RL56	7	RM56	3	RH56	6
M57	0.01×R2A	BL57	10	BM57	5	BH57	9	RL57	13	RM57	3	RH57	10
M58	1×MA	BL58	6	BM58	3	BH58	3	RL58	7	RM58	3	RH58	3
M59	0.1×MA	BL59	6	BM59	3	BH59	3	RL59	10	RM59	3	RH59	9
M60	0.01×MA	BL60	10	BM60	5	BH60	9	RL60	13	RM60	3	RH60	10

Abbreviations: BL (bulk soil cultures at low temperature), BM (bulk soil cultures at medium temperature), BH (bulk soil cultures at high temperature), RL (rhizosphere cultures at low temperature), RM (rhizosphere cultures at medium temperature) and RH (rhizosphere cultures at high temperature).

Supplementary Dataset

Supplementary Dataset 1 Microbial culture characteristics of bulk and rhizosphere soil samples on 60 media at three temperatures. The left color blocks represent bulk soil (orange) and rhizosphere soil (green-cyan) and the top color blocks represent 15 ℃ (cyan-blue), 30 ℃ (green) and 45 ℃ (red), respectively. The full names of the culture media are shown in Supplementary Table 8.

List:

Medium No.	Medium Name
M1-M4	BEN | BHI | BRA | CSA
M5-M8	CHIA | CZA | GN1 | GYM
M9-M12	GLA | GGA | HA | HVA
M13-M16	KB | LNMS | LB | MCA
M17-M20	MYP | MIC | MM | PYG
M21-M24	PYS | PPS | PDA | RA
M25-M28	RH | RZ1 | RZ2 | SA
M29-M32	SSM | SSTY | CMCA | SLA
M33-M36	SEAB | SEAR | SCA | SGCY
M37-M40	SNA | STA | STBG | T5
M41-M44	TP | TGY | TCN | YGCP
M45-M48	YMG | 1×TSA | 0.1×TSA | 0.01×TSA
M49-M52	1×NA | 0.1×NA | 0.01×NA | 1×AIA
M53-M56	0.1×AIA | 0.01×AIA | 1×R2A | 0.1×R2A
M57-M60	0.01×R2A | 1×MA | 0.1×MA | 0.01×MA

M1 (BEN)

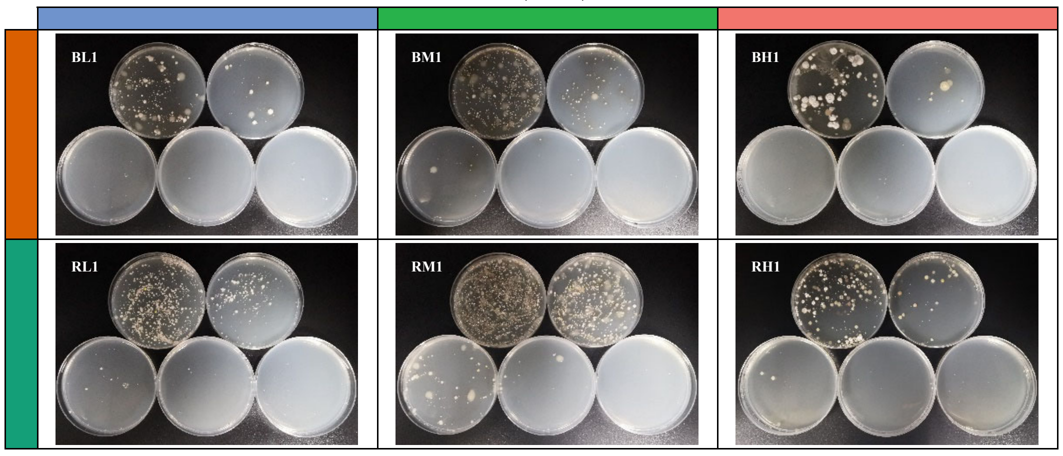

M2 (BHI)

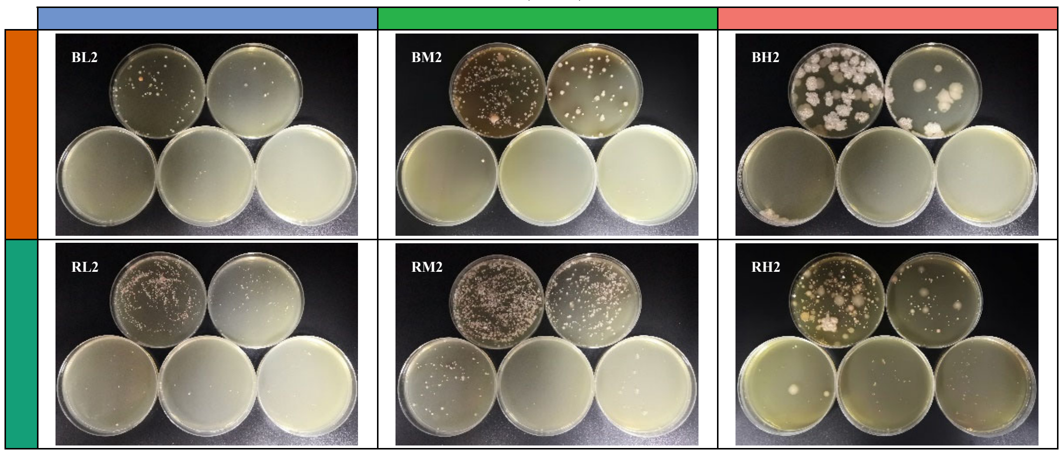

M3 (BRA)

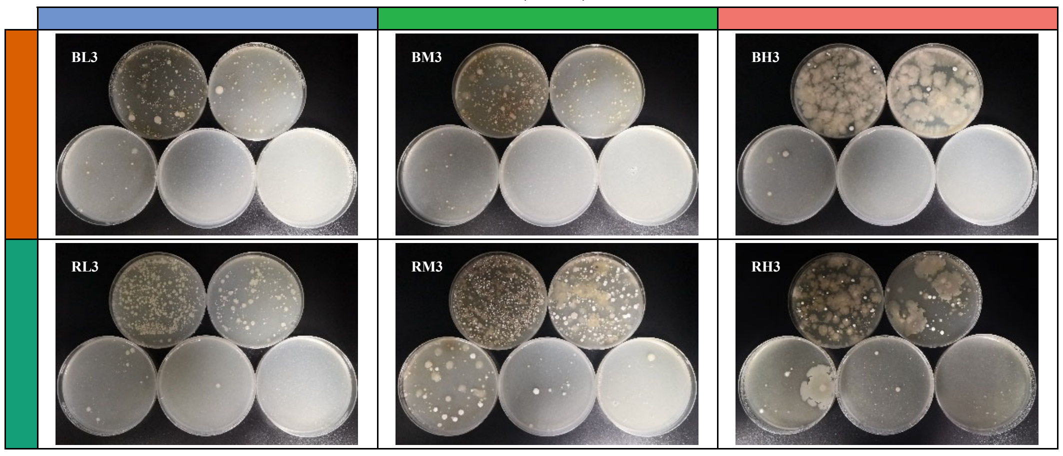

M4 (CSA)

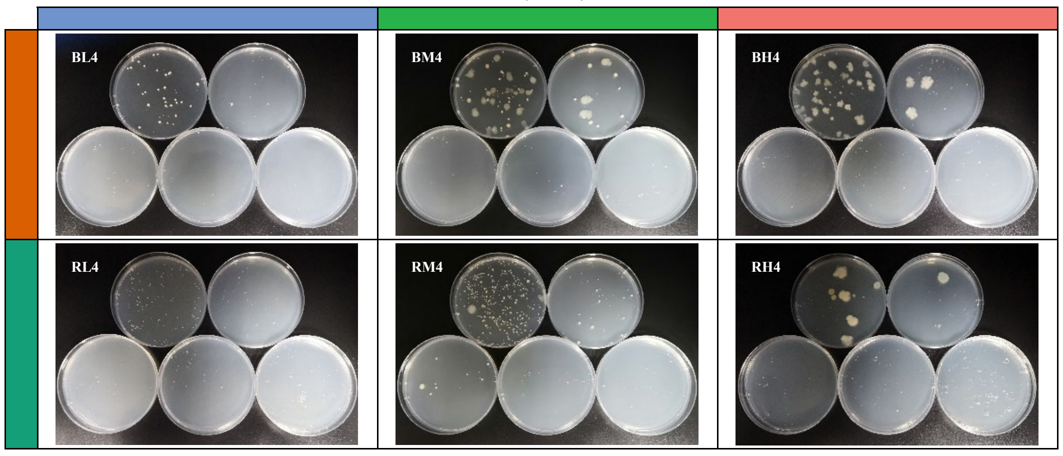

M5 (CHIA)

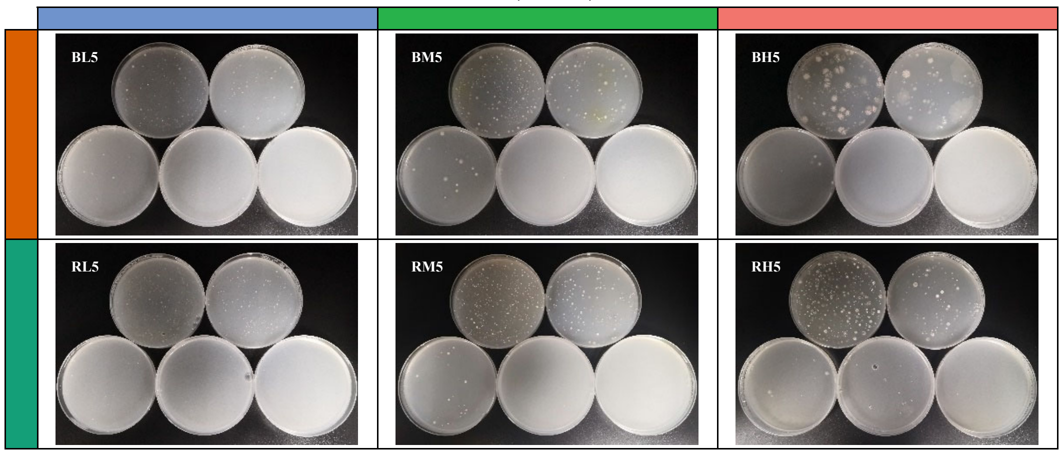

M6 (CZA)

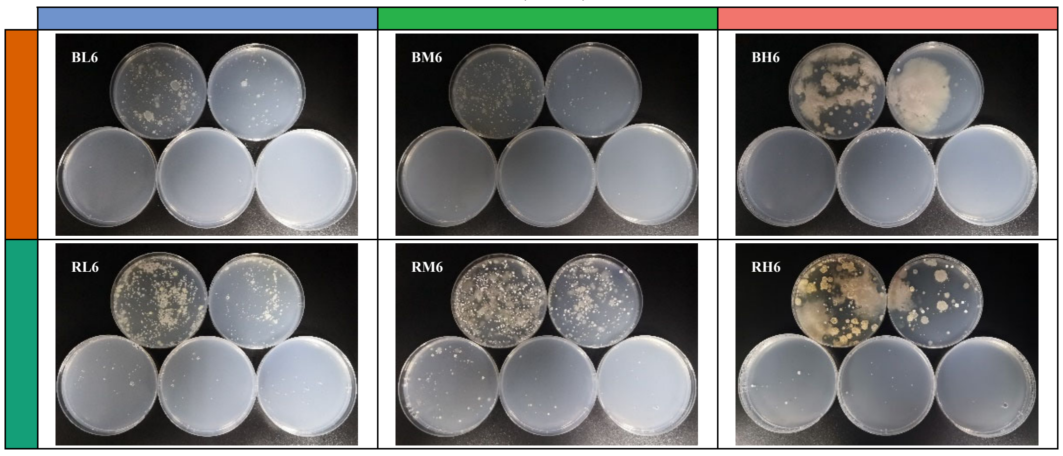

M7 (GN1)

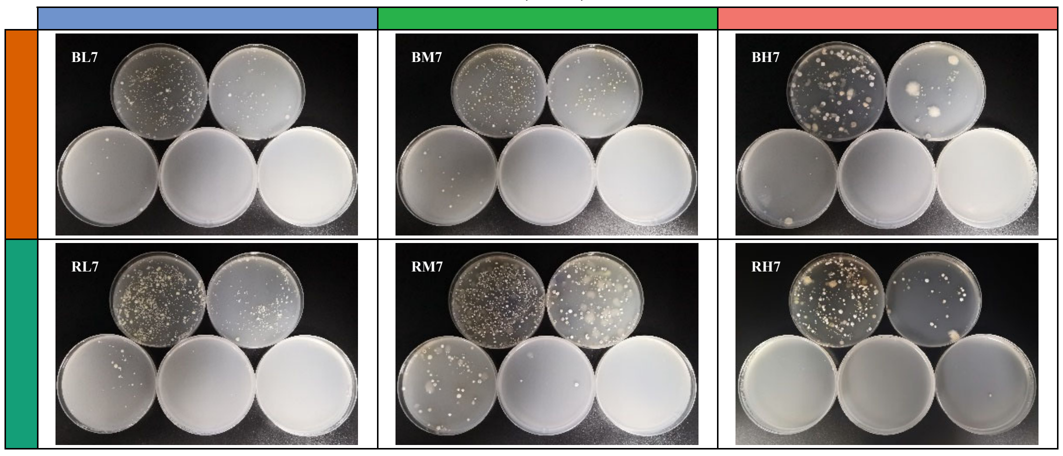

M8 (GYM)

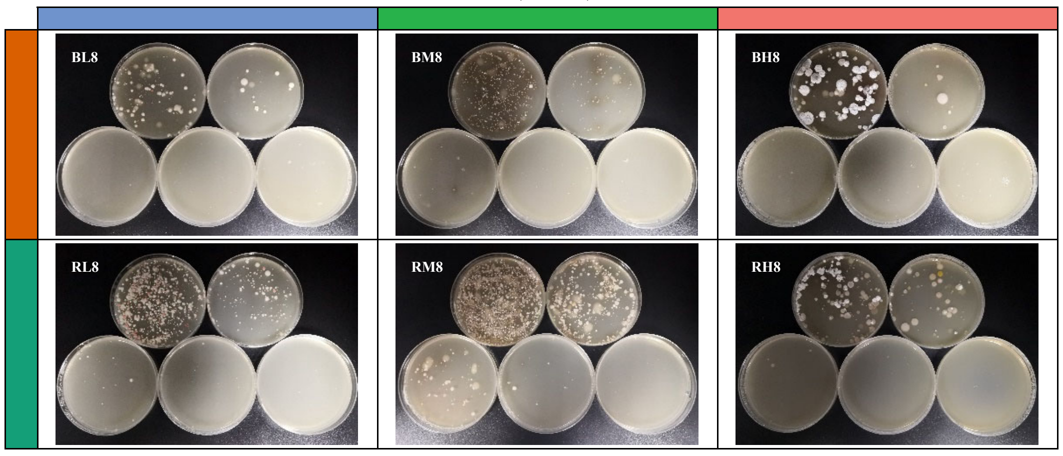

M9 (GLA)

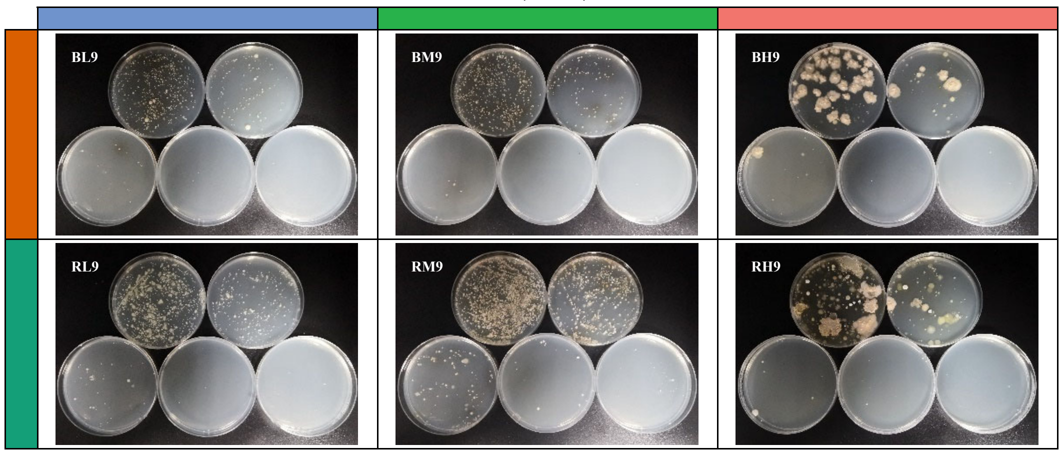

M10 (GGA)

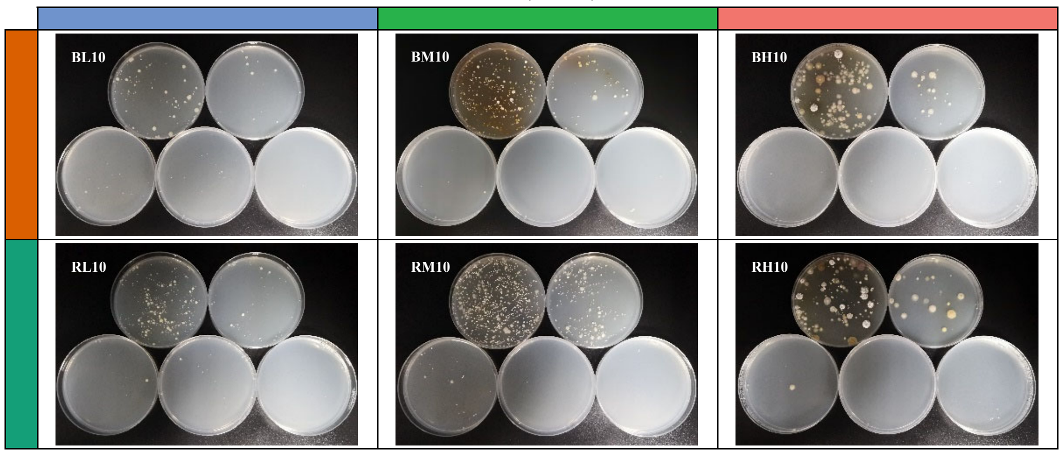

M11 (HA)

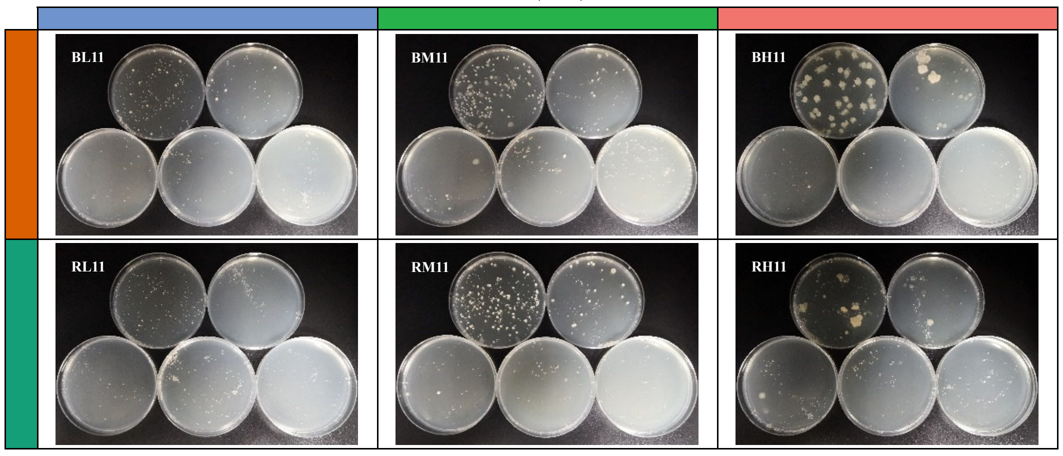

M12 (HVA)

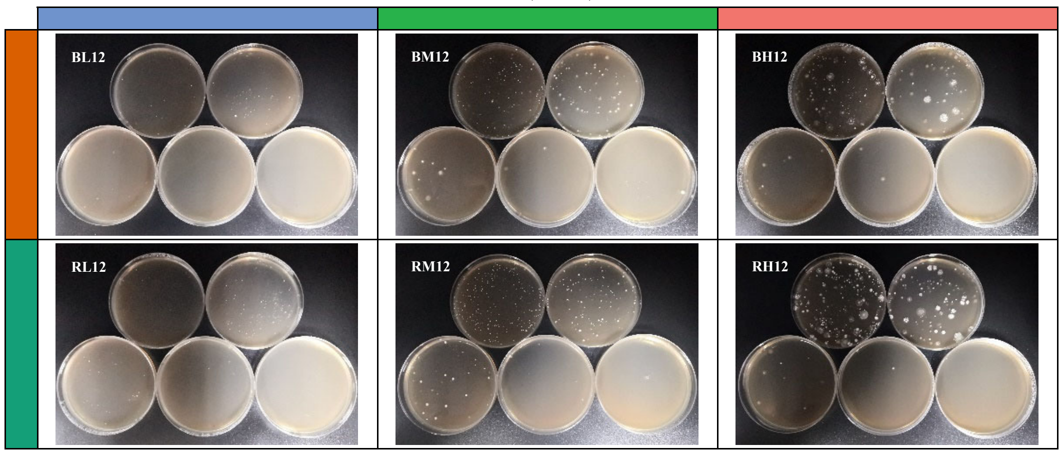

M13 (KB)

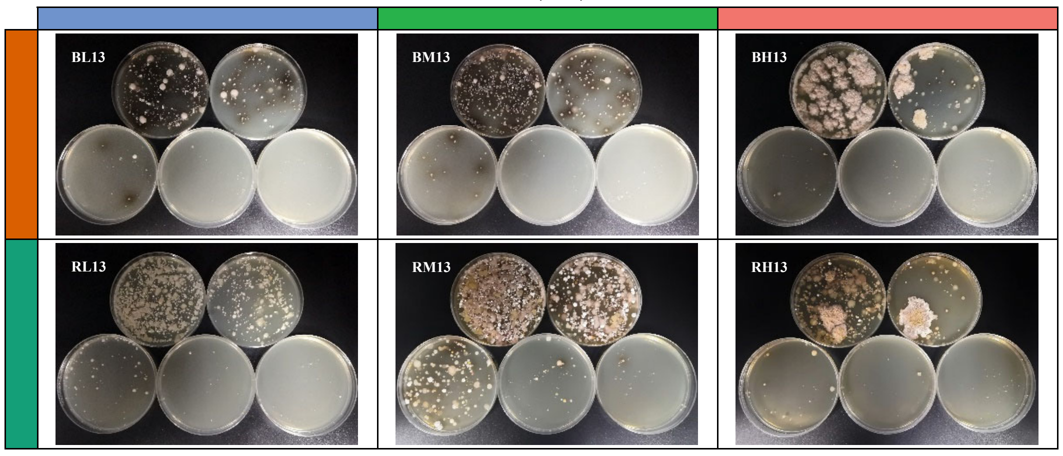

M14 (LNMS)

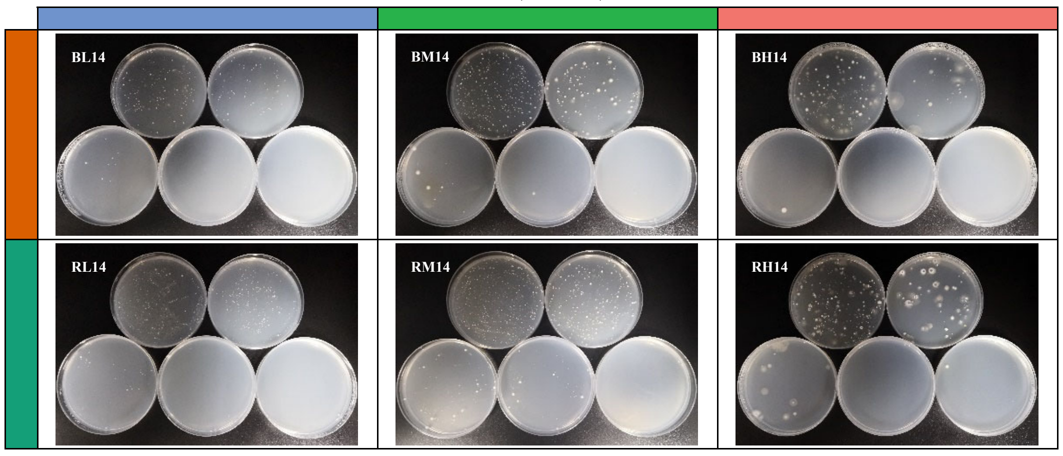

M15 (LB)

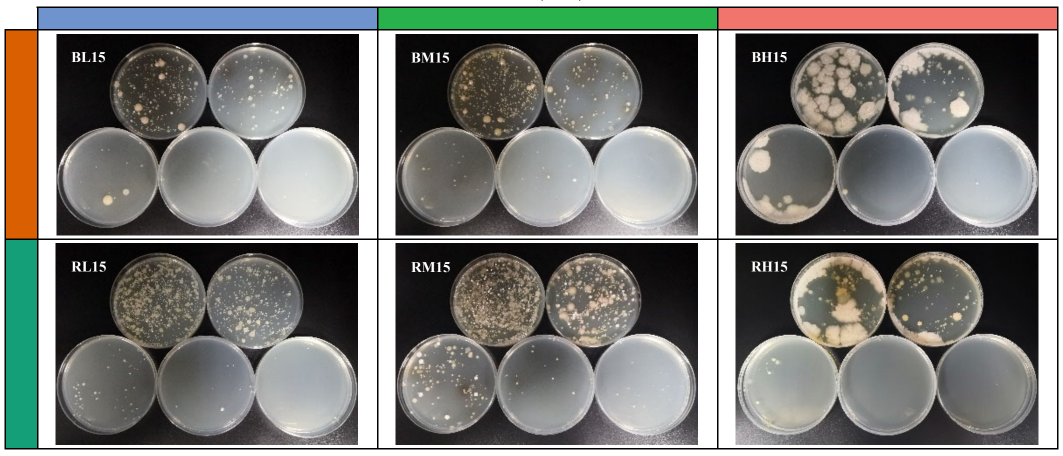

M16 (MCA)

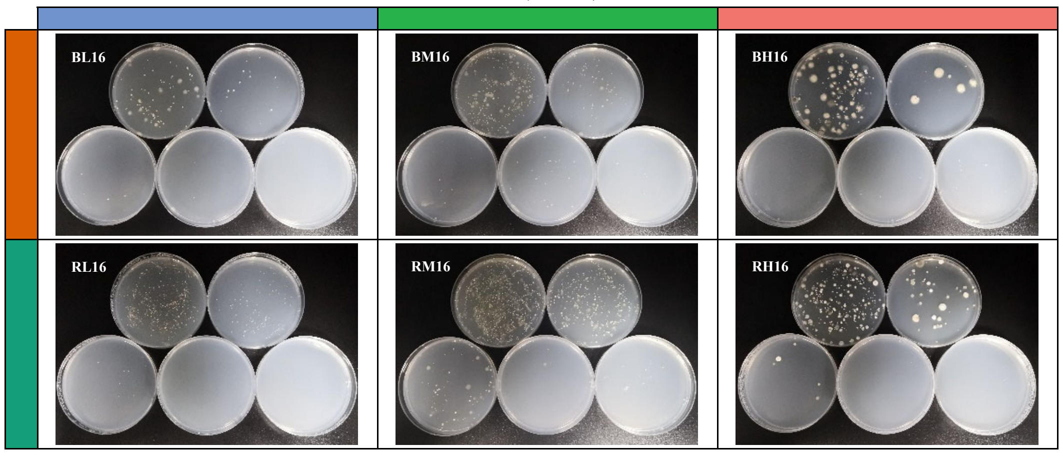

M17 (MYP)

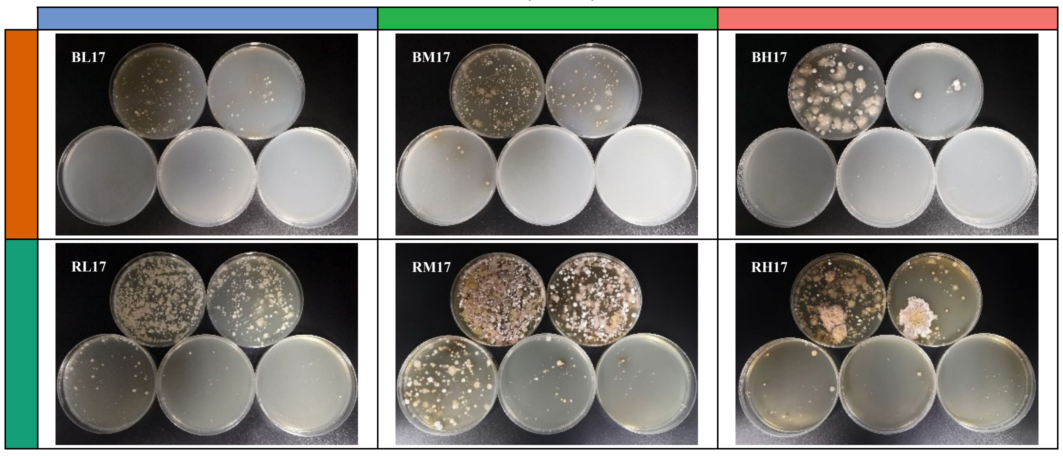

M18 (MIC)

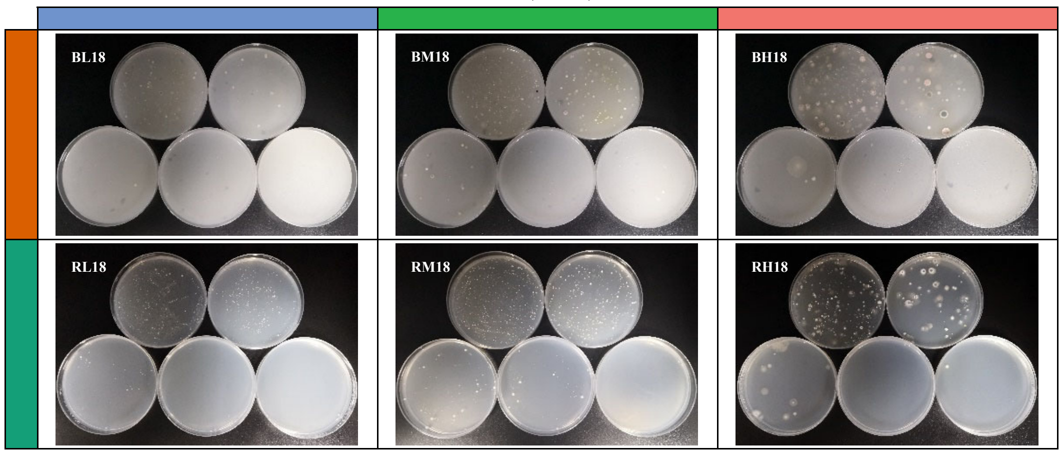

M19 (MM)

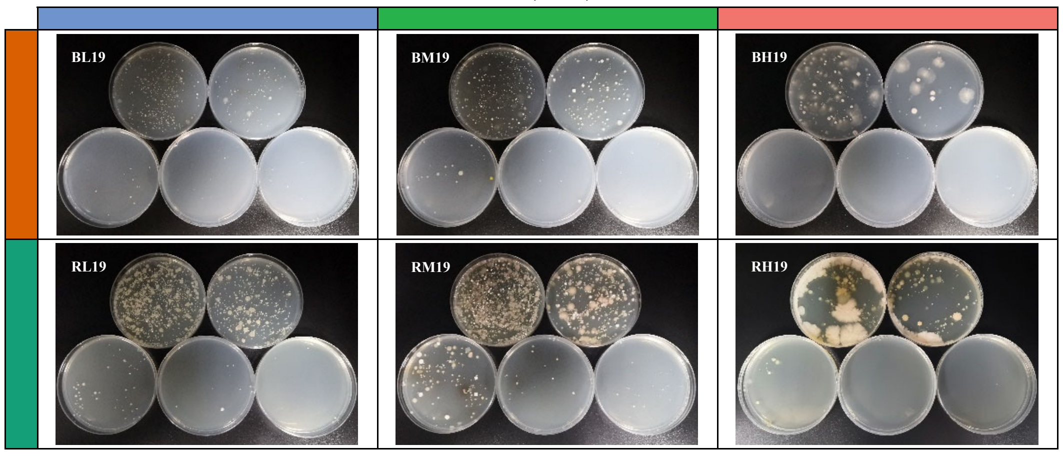

M20 (PYG)

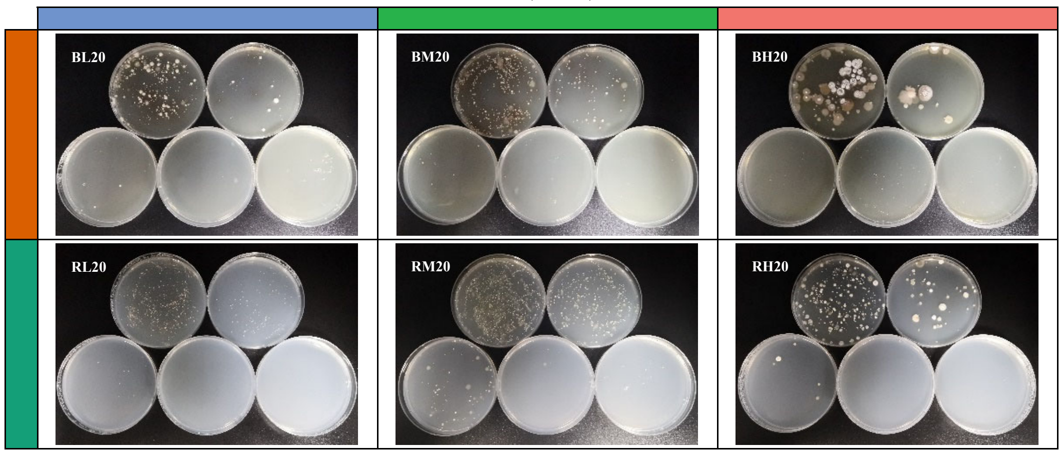

M21 (PYS)

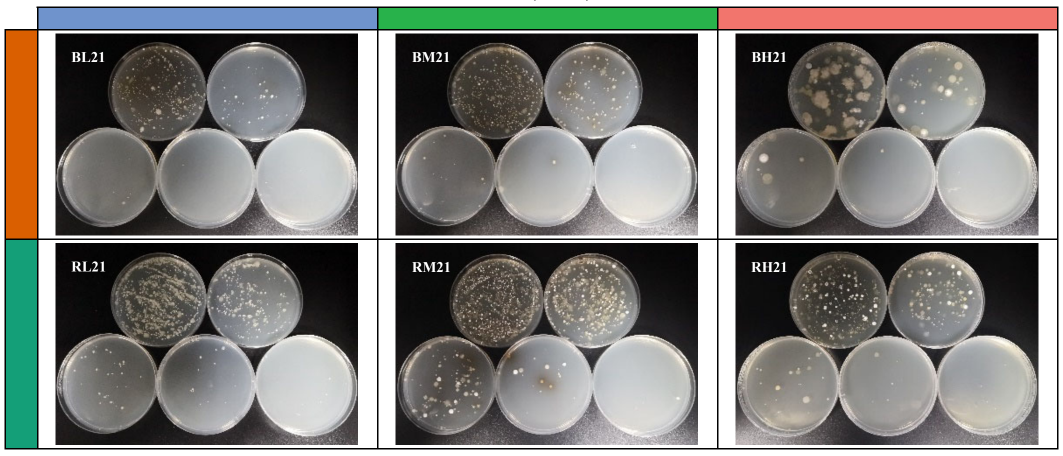

M22 (PPS)

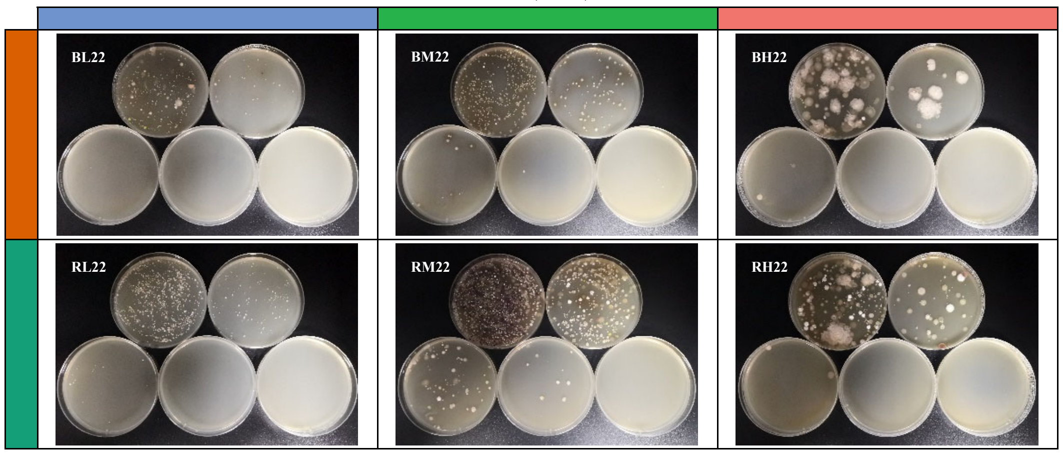

M23 (PDA)

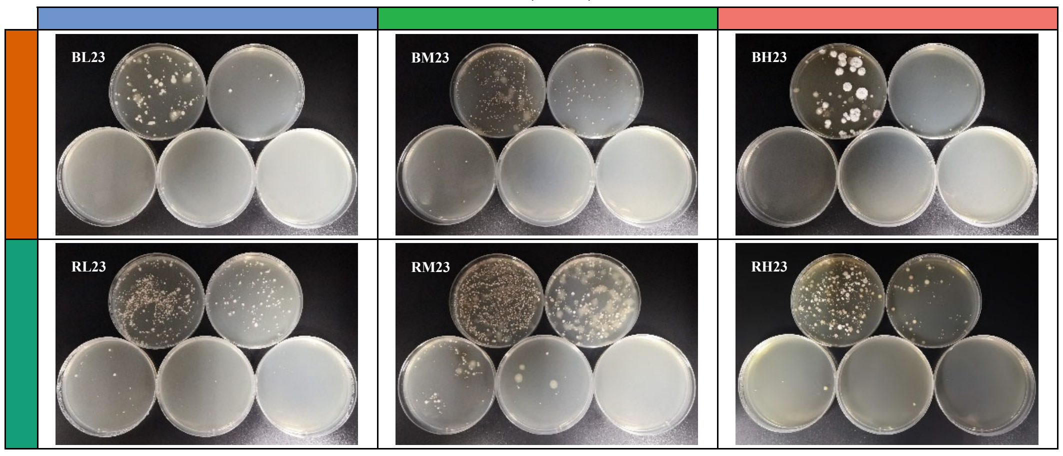

M24 (RA)

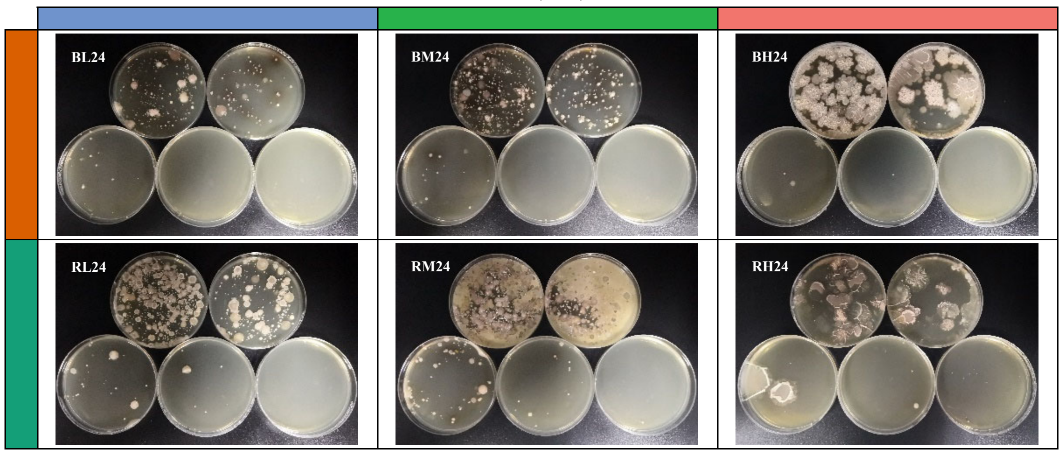

M25 (RH)

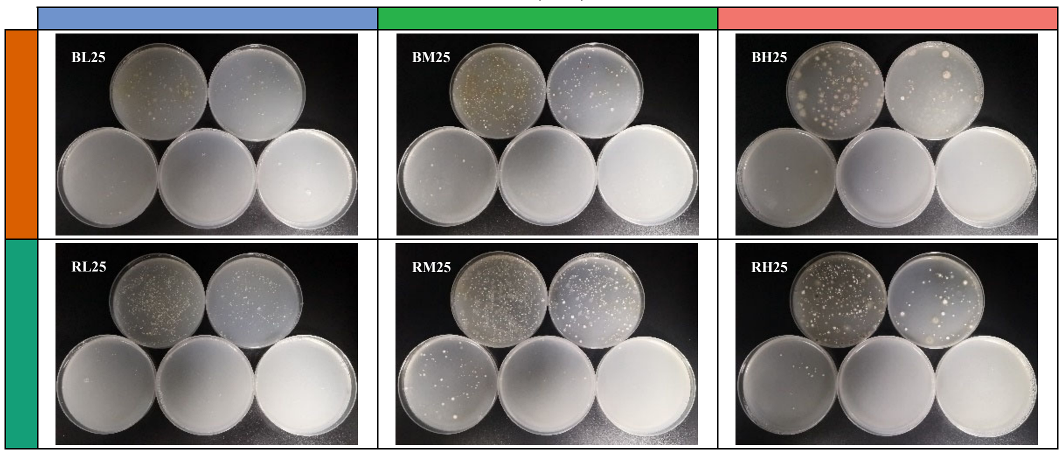

M26 (RZ1)

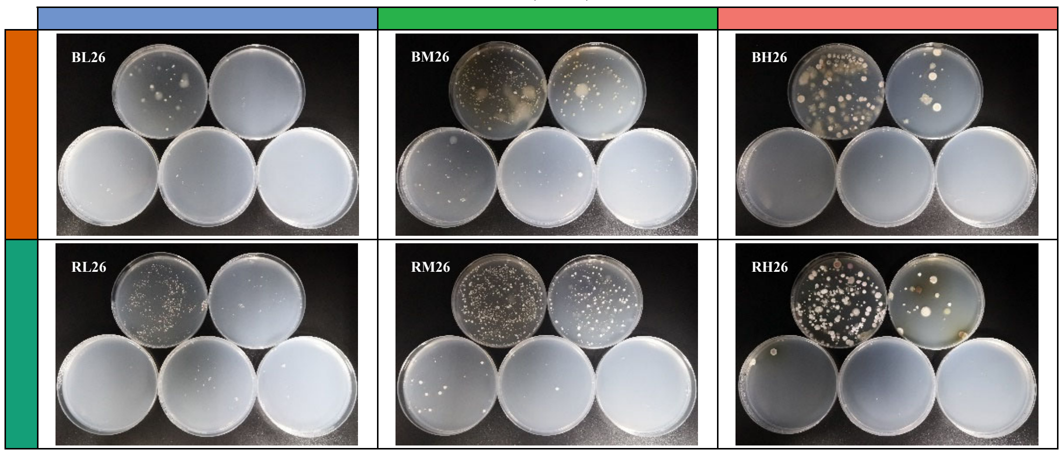

M27 (RZ2)

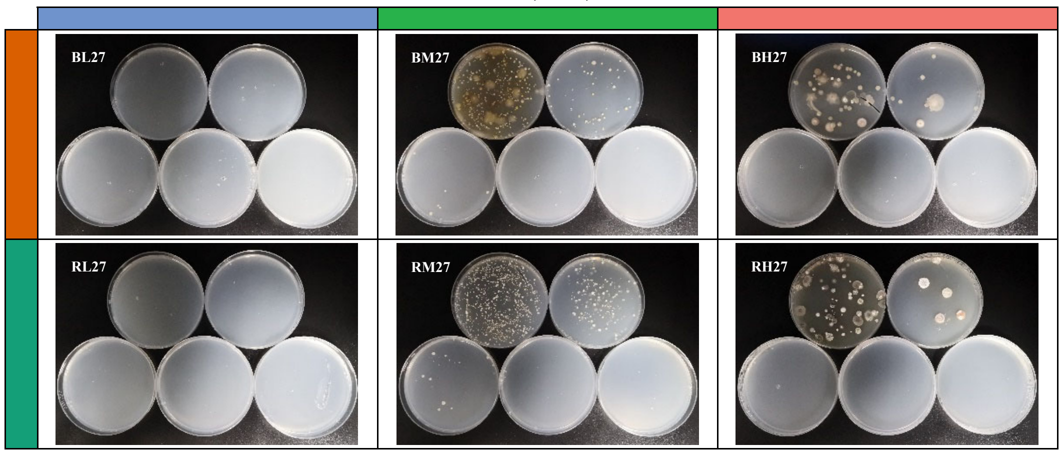

M28 (SA)

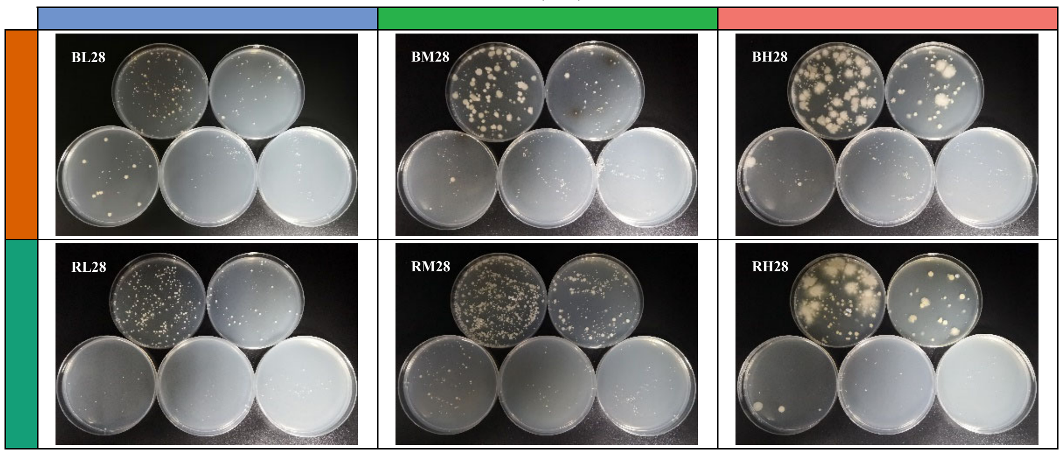

M29 (SSM)

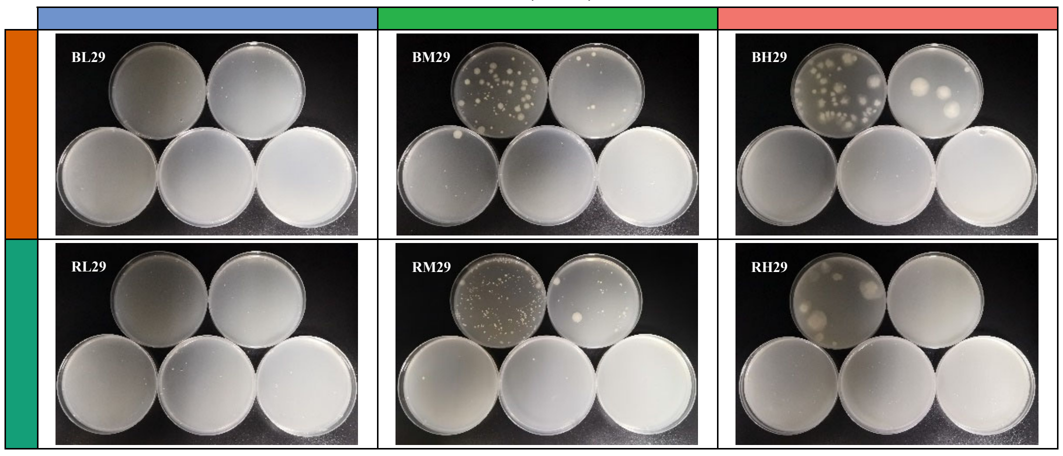

M30 (SSTY)

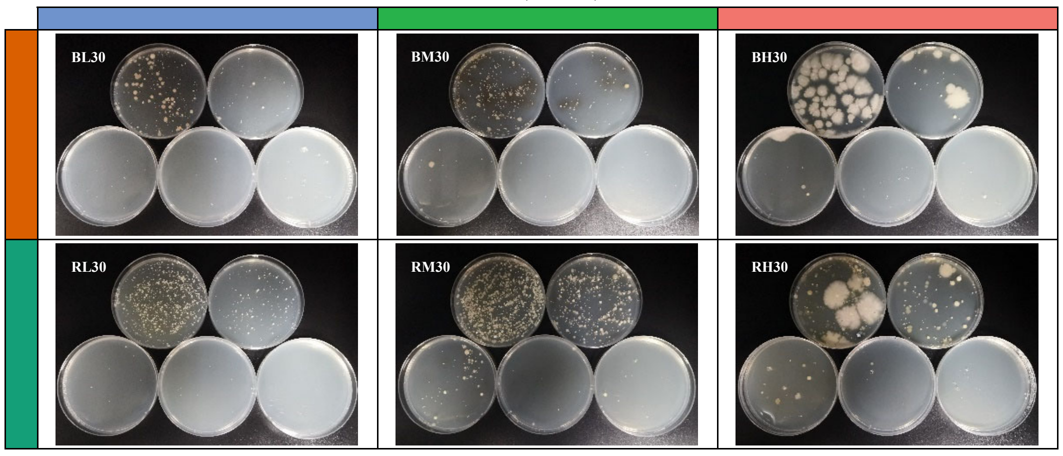

M31 (CMCA)

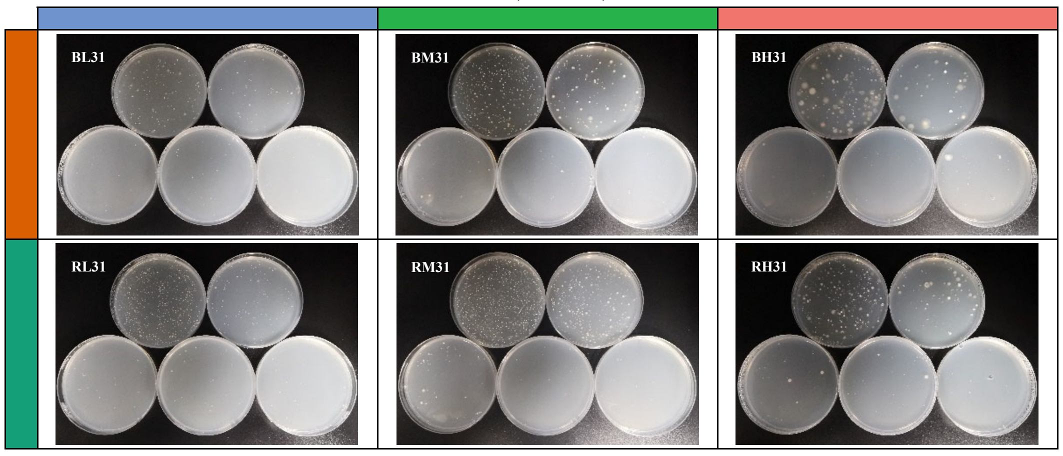

M32 (SLA)

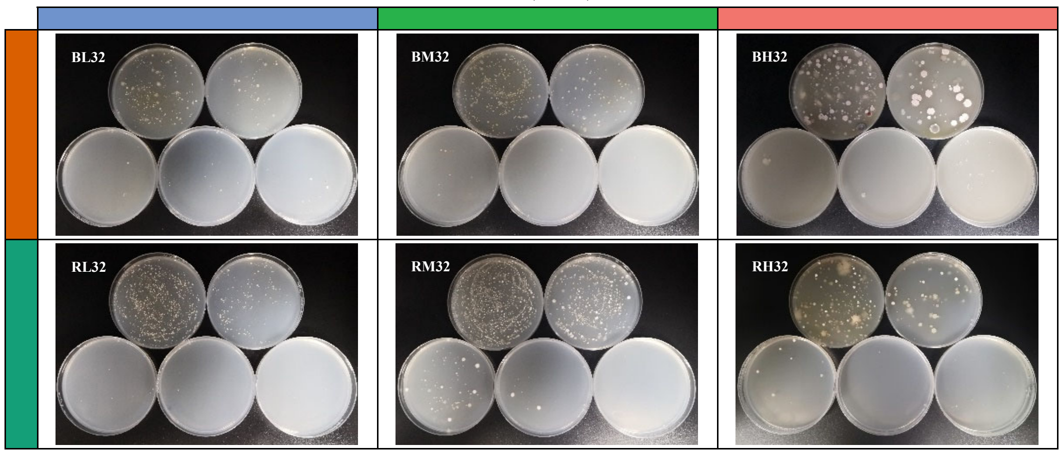

M33 (SEAB)

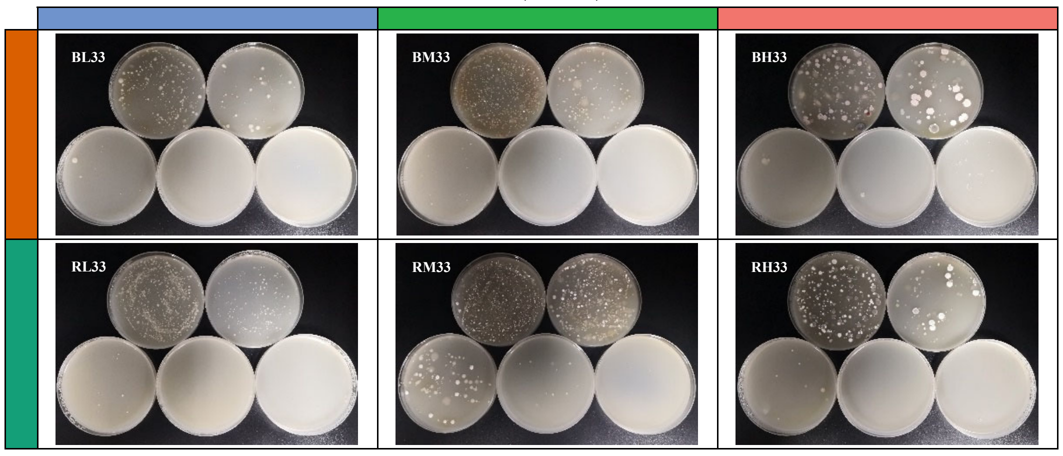

M34 (SEAR)

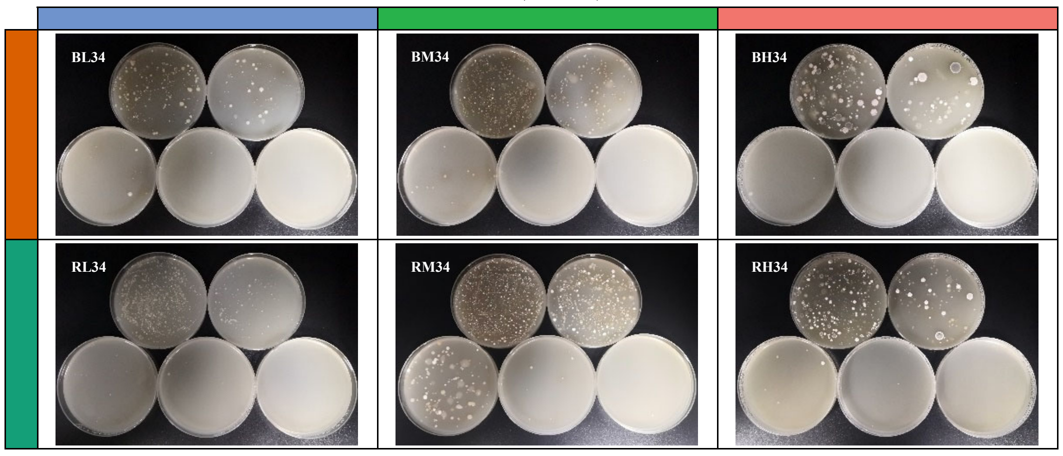

M35 (SCA)

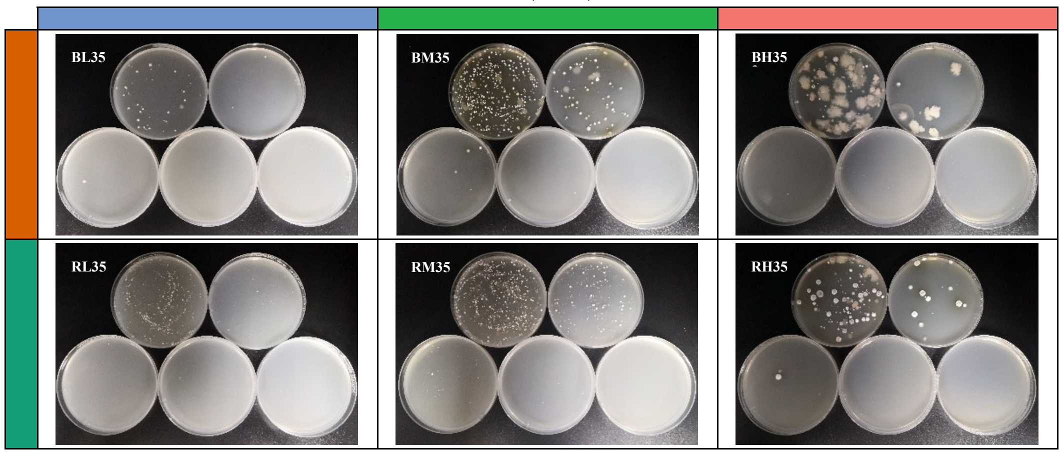

M36 (SGCY)

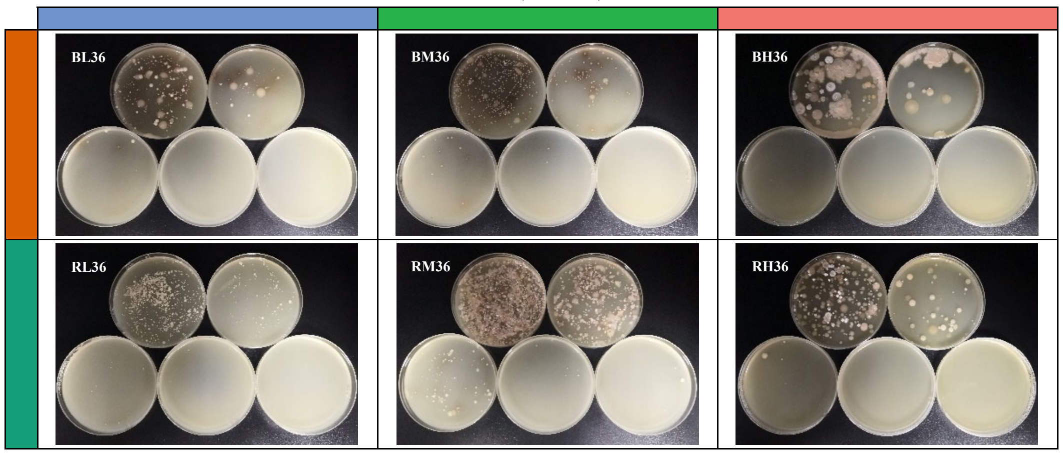

M37 (SNA)

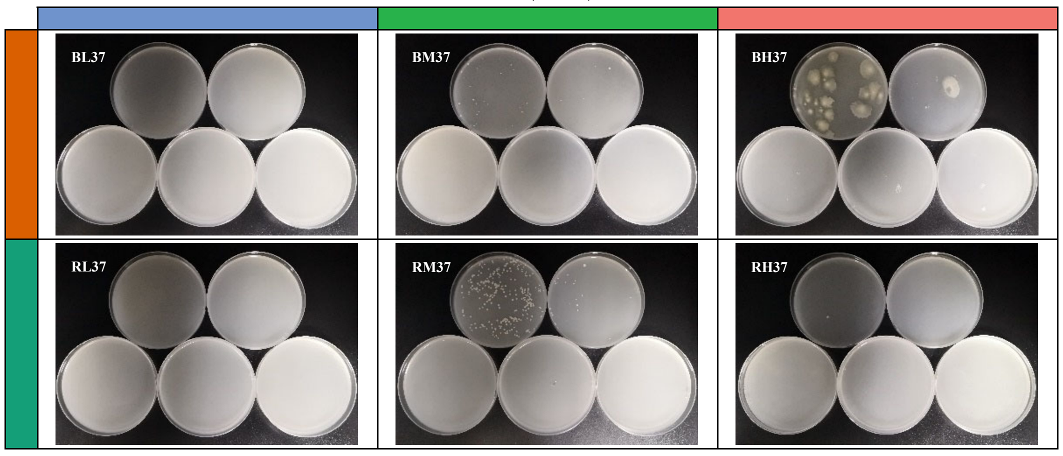

M38 (STA)

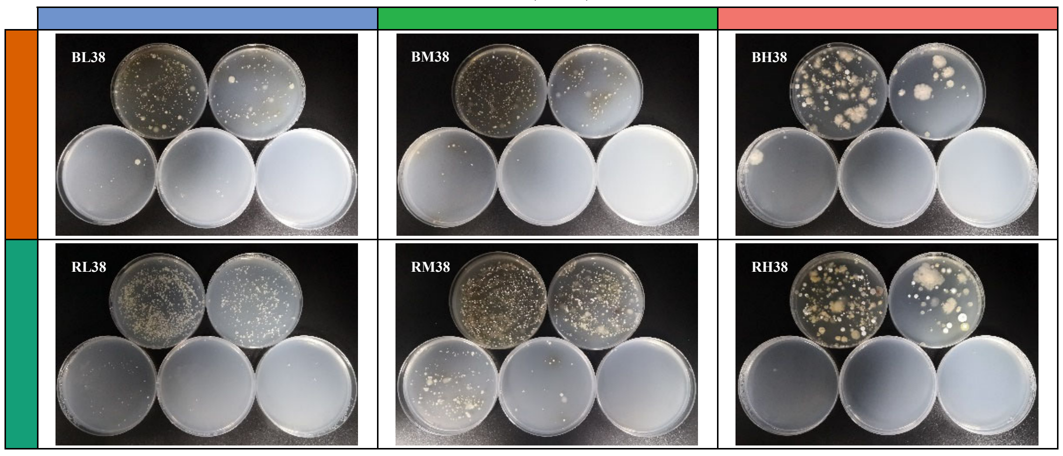

M39 (STBG)

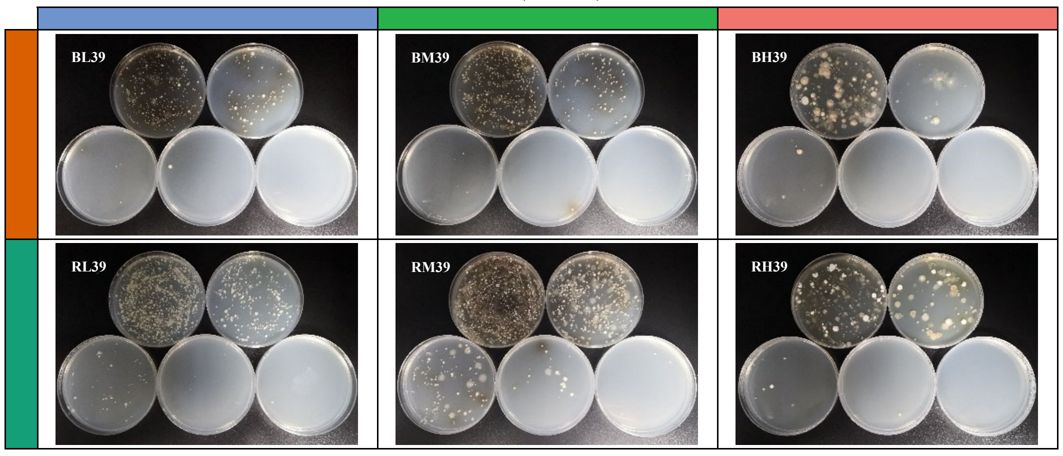

M40 (T5)

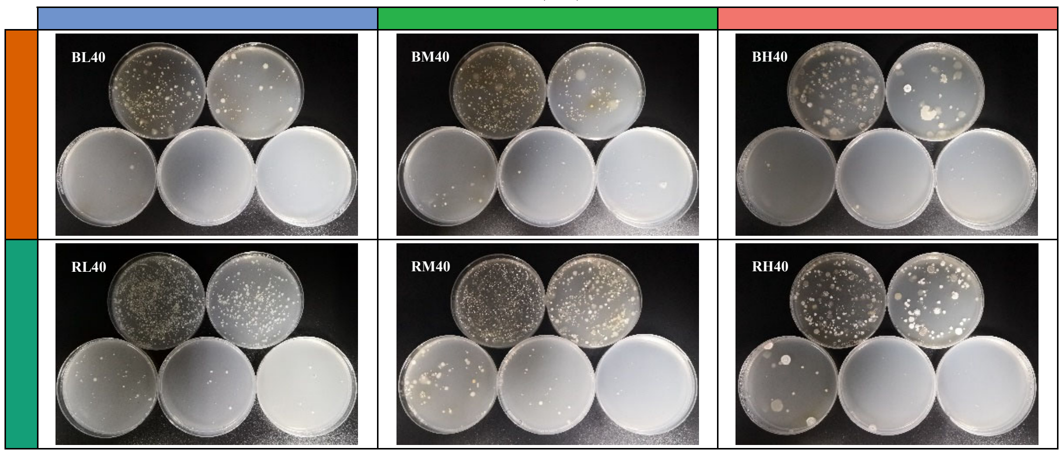

M41 (TP)

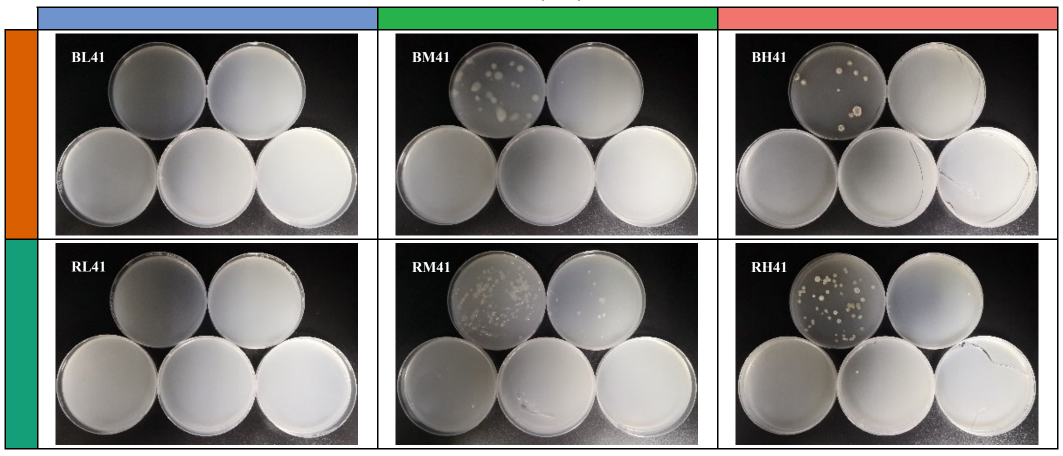

M42 (TGY)

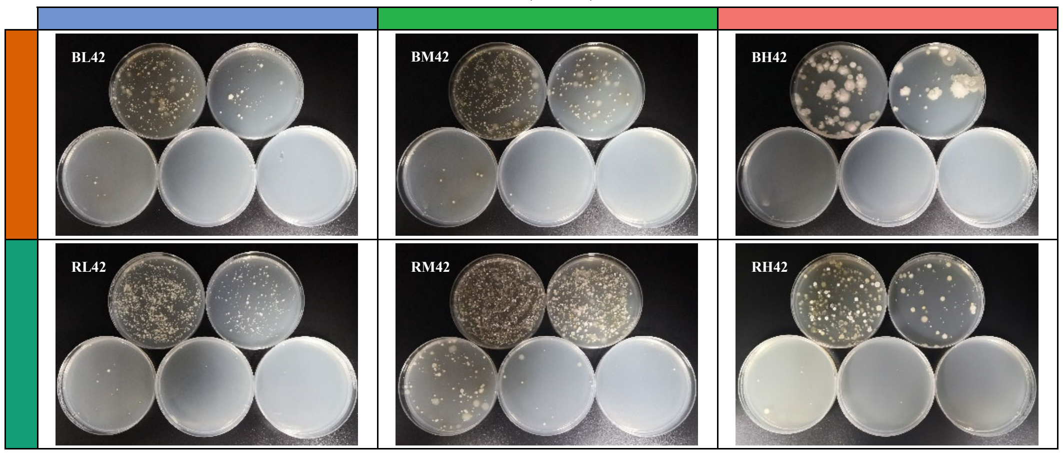

M43 (TCN)

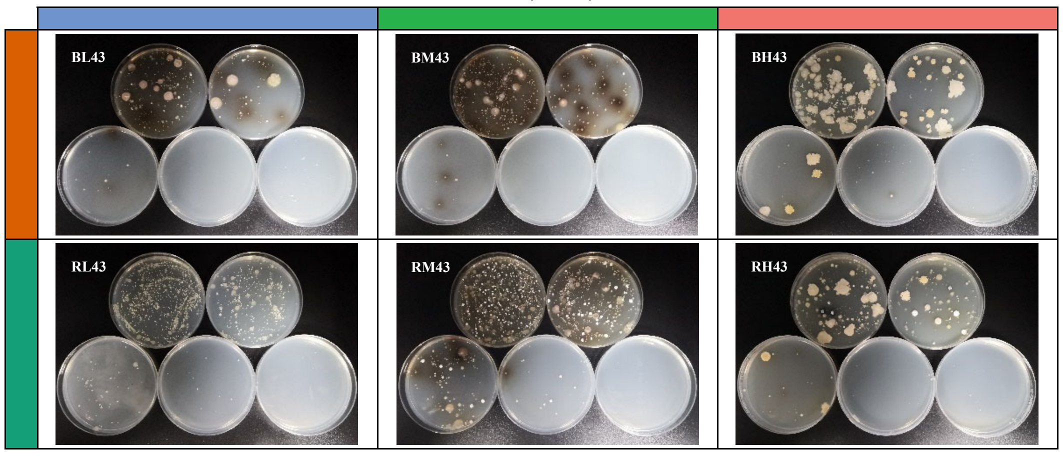

M44 (YGCP)

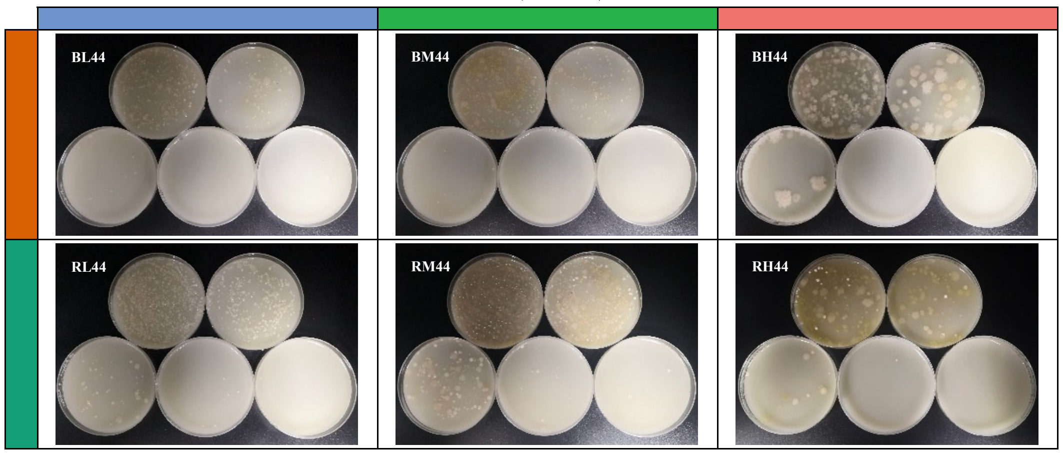

M45 (YMG)

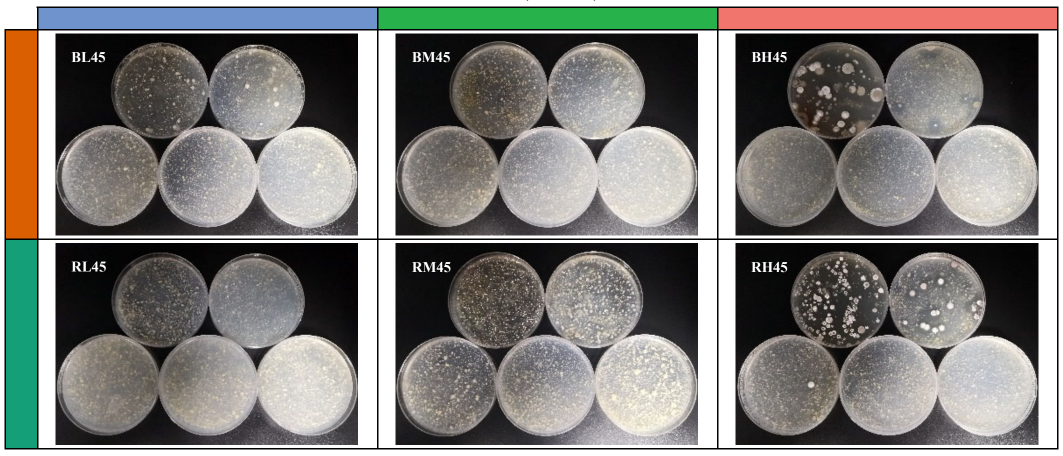

M46 (1×TSA)

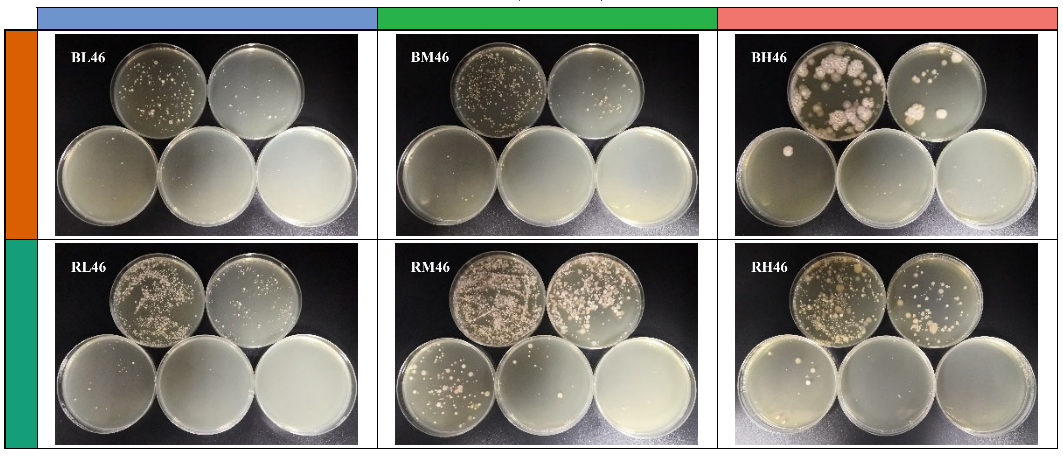

M47 (0.1×TSA)

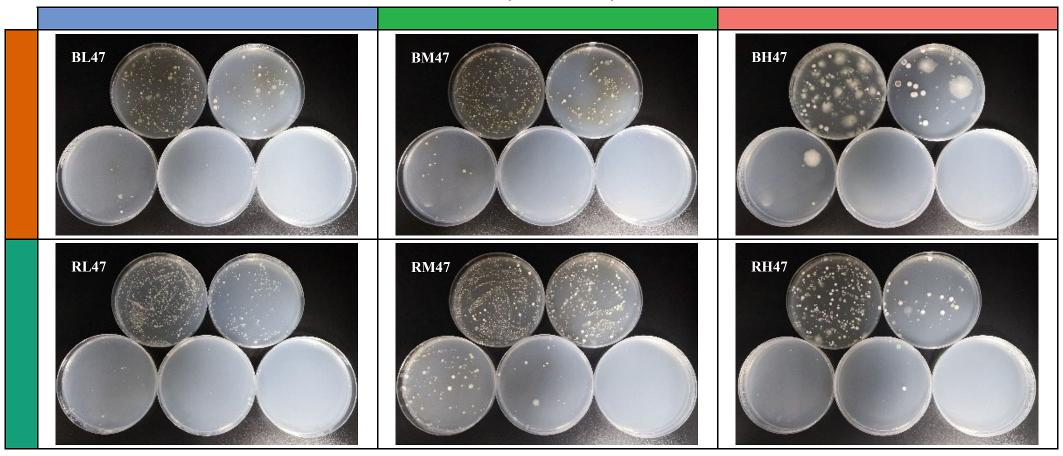

M48 (0.01×TSA)

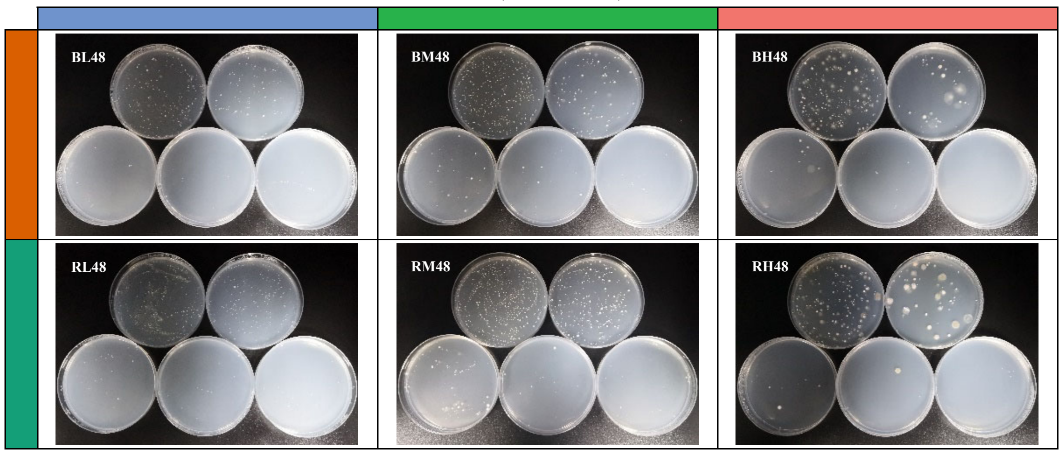

M49 (1×NA)

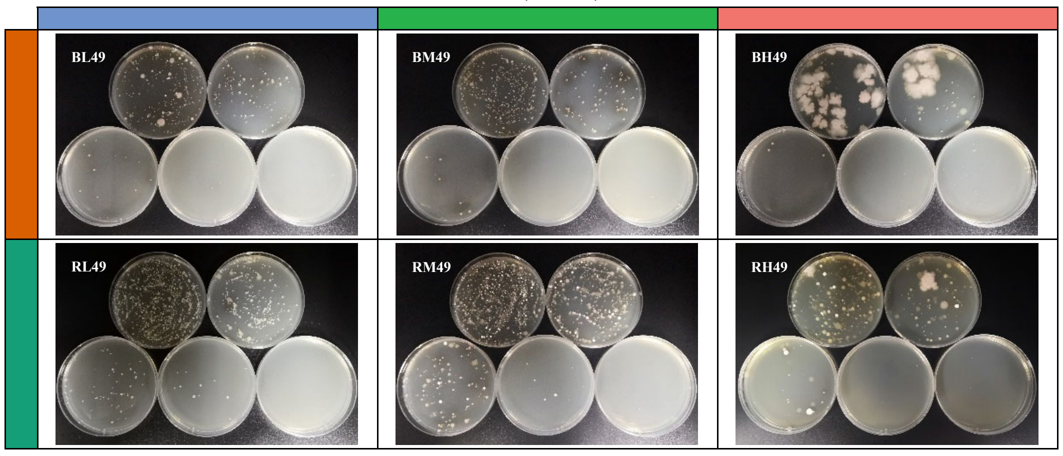

M50 (0.1×NA)

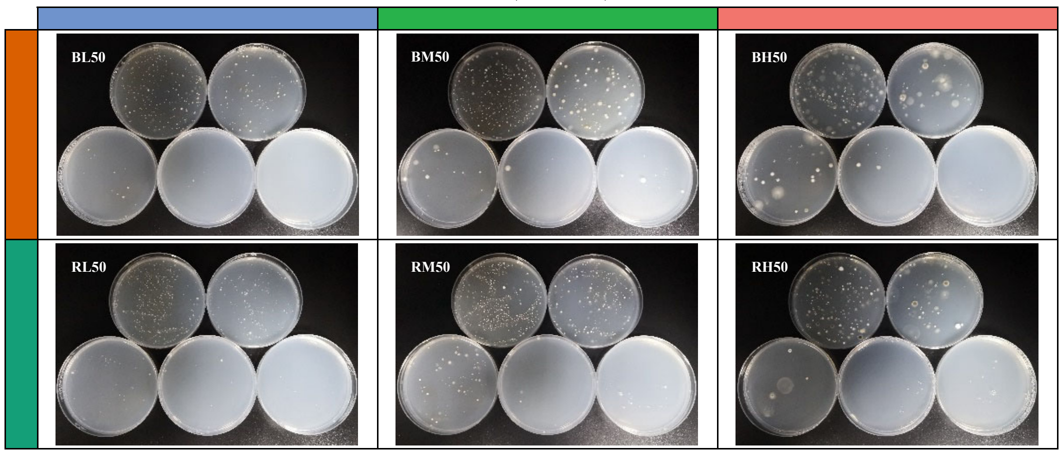

M51 (0.01×NA)

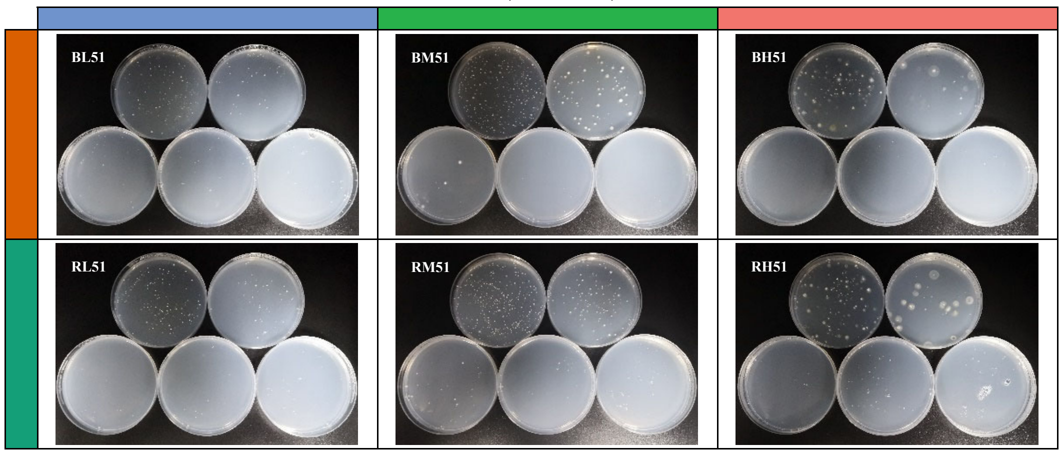

M52 (1×AIA)

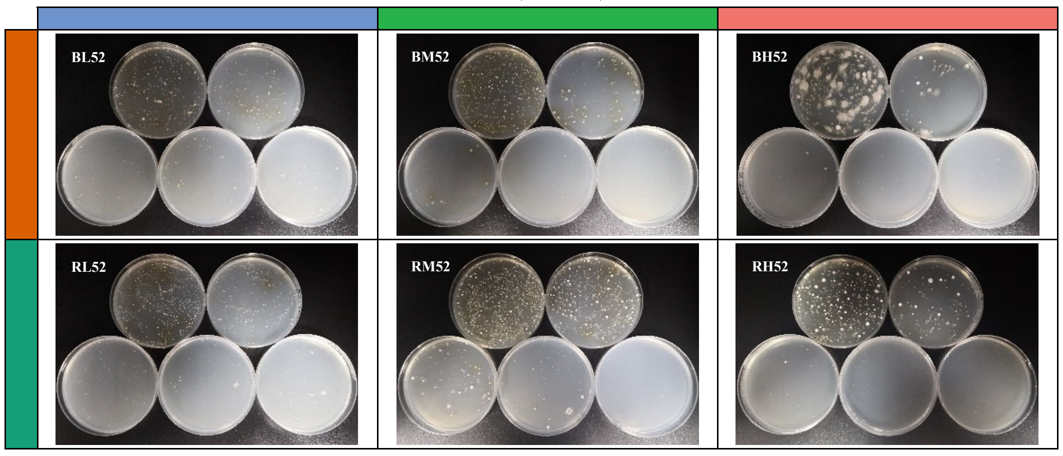

M53 (0.1×AIA)

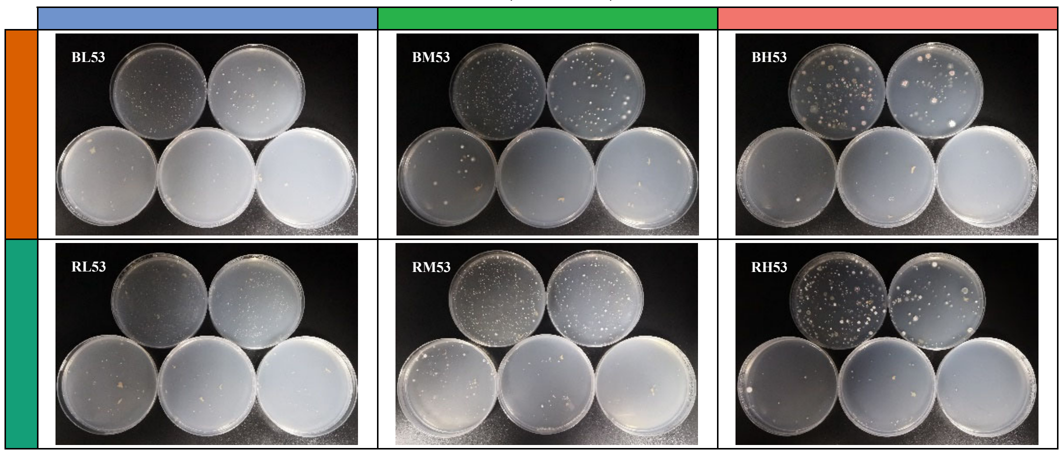

M54 (0.01×AIA)

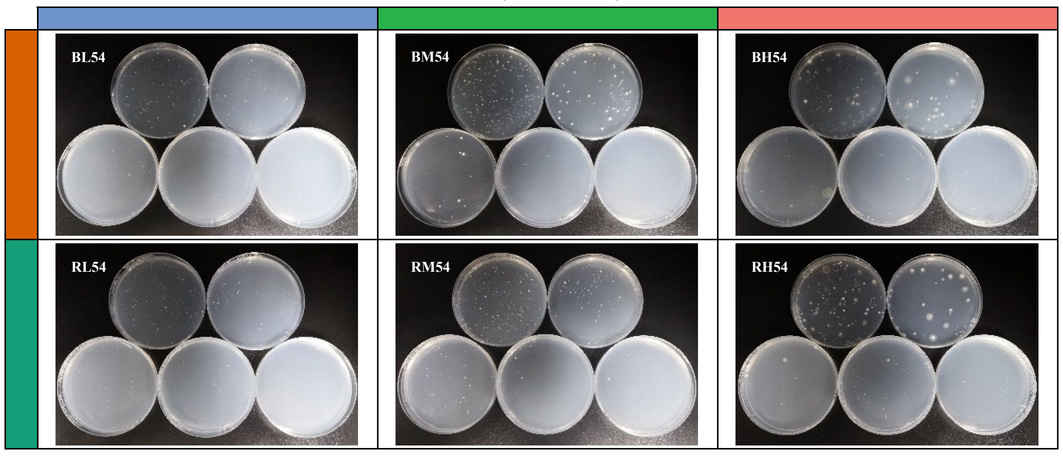

M55 (1×R2A)

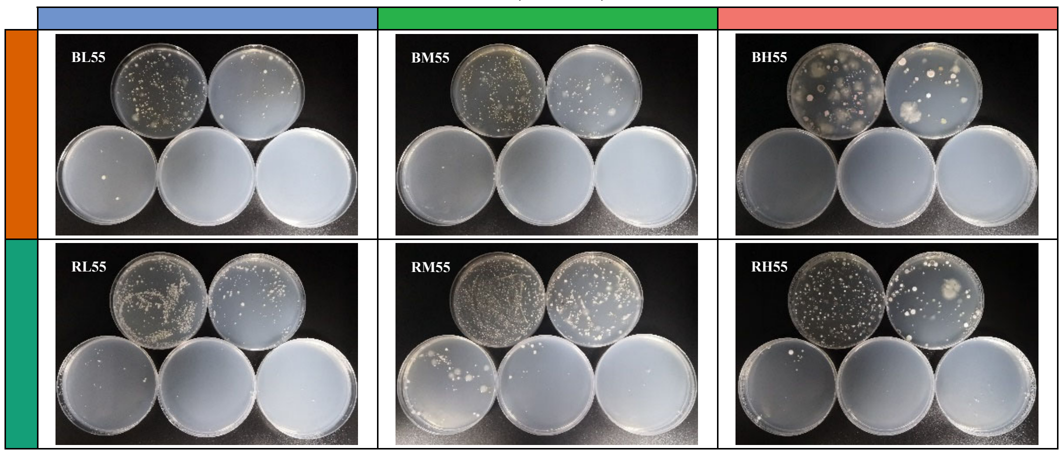

M56 (0.1×R2A)

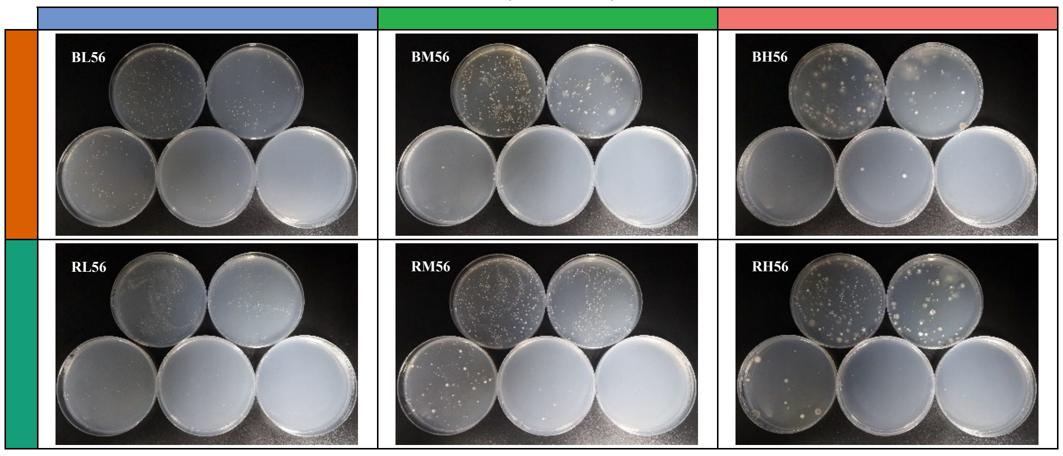

M57 (0.01×R2A)

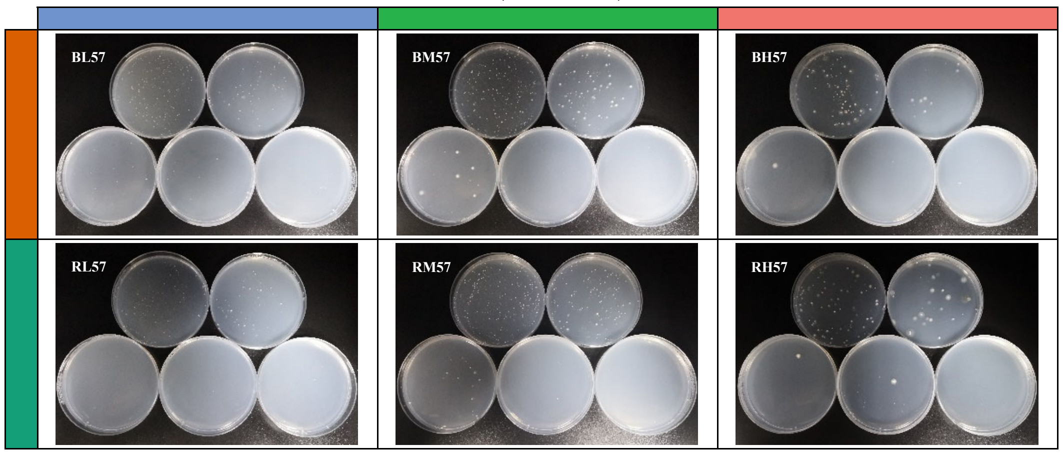

M58 (1×MA)

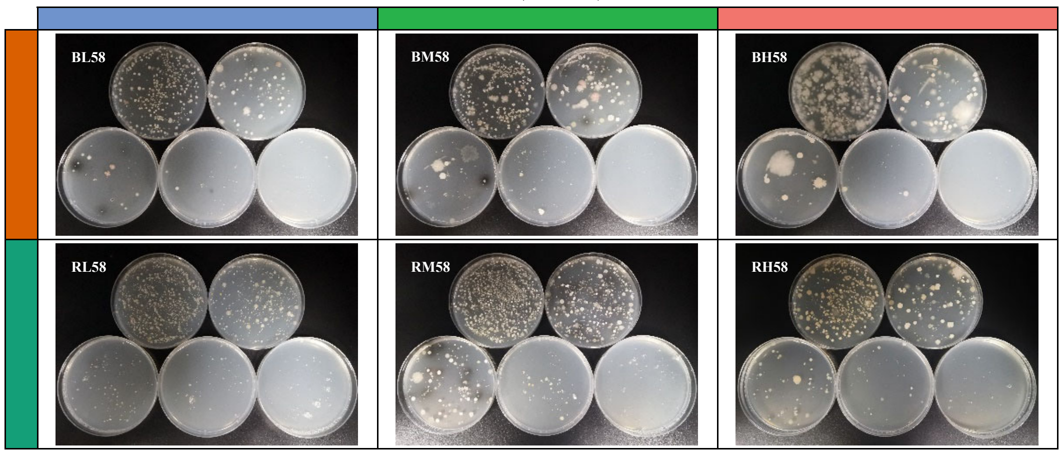

M59 (0.1×MA)

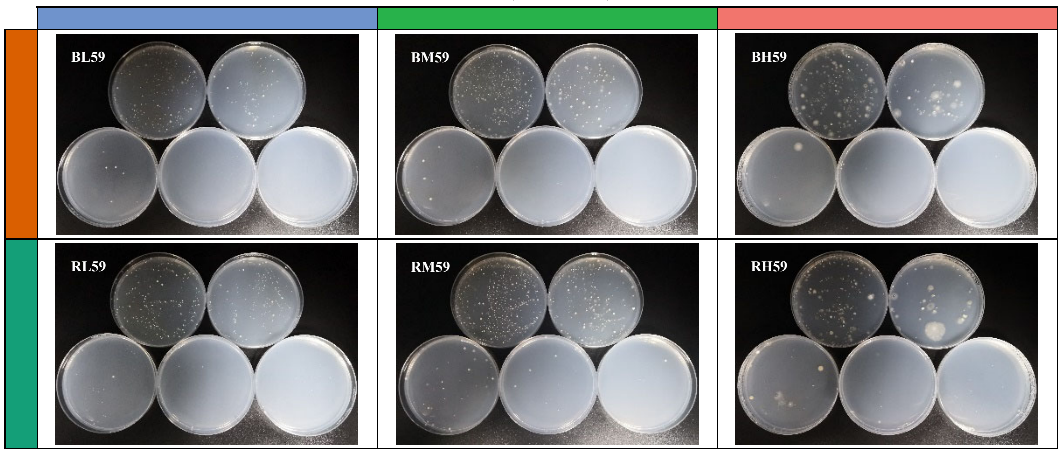

M60 (0.01×MA)

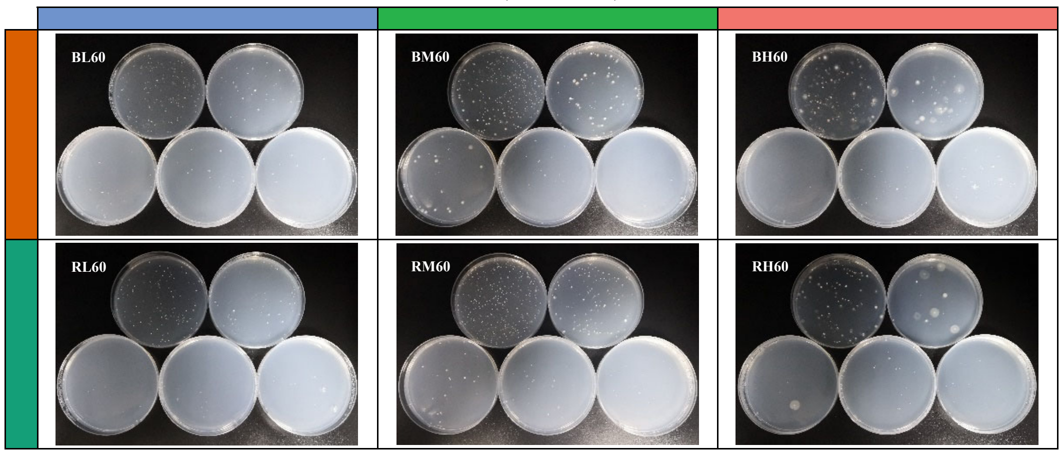

Supplement: Supplementary file 1 — Supplementary Information [file 41522_2023_439_MOESM1_ESM.pdf]
