# Supplementary material for: Capturing the microbial dark matter in desert soils using culturomics-based metagenomics and high-resolution analysis
Source: NPJ Biofilms Microbiomes. 2023 Sep 22;9:67. doi: 10.1038/s41522-023-00439-8 (PMC10516943; doi:10.1038/s41522-023-00439-8)
Supplement: Supplementary file 2 — Reporting Summary [file 41522_2023_439_MOESM2_ESM.pdf]

nature portfolio

Corresponding author(s): Prof Wen- Jun Li

Last updated by author(s): Sep 4, 2023

# Reporting Summary

Nature Portfolio wishes to improve the reproducibility of the work that we publish. This form provides structure for consistency and transparency in reporting. For further information on Nature Portfolio policies, see our Editorial Policies and the Editorial Policy Checklist.

## Statistics

For all statistical analyses, confirm that the following items are present in the figure legend, table legend, main text, or Methods section.

| n/a | Confirmed | |
|---|---|---|
| ☐ | ☒ | The exact sample size (*n*) for each experimental group/condition, given as a discrete number and unit of measurement |
| ☐ | ☒ | A statement on whether measurements were taken from distinct samples or whether the same sample was measured repeatedly |
| ☐ | ☒ | The statistical test(s) used AND whether they are one- or two-sided<br>*Only common tests should be described solely by name; describe more complex techniques in the Methods section.* |
| ☐ | ☒ | A description of all covariates tested |
| ☐ | ☒ | A description of any assumptions or corrections, such as tests of normality and adjustment for multiple comparisons |
| ☐ | ☒ | A full description of the statistical parameters including central tendency (e.g. means) or other basic estimates (e.g. regression coefficient) AND variation (e.g. standard deviation) or associated estimates of uncertainty (e.g. confidence intervals) |
| ☐ | ☒ | For null hypothesis testing, the test statistic (e.g. *F*, *t*, *r*) with confidence intervals, effect sizes, degrees of freedom and *P* value noted<br>*Give P values as exact values whenever suitable.* |
| ☒ | ☐ | For Bayesian analysis, information on the choice of priors and Markov chain Monte Carlo settings |
| ☒ | ☐ | For hierarchical and complex designs, identification of the appropriate level for tests and full reporting of outcomes |
| ☐ | ☒ | Estimates of effect sizes (e.g. Cohen's *d*, Pearson's *r*), indicating how they were calculated |

*Our web collection on statistics for biologists contains articles on many of the points above.*

## Software and code

Policy information about availability of computer code

| | |
|---|---|
| Data collection | Venn diagrams were used to present the shared and unique components among groups using the Biozeron Cloud Platform (http://www.cloud.biomicroclass.com/CloudPlatform).<br>To construct the ASV-based phylogenetic trees, ASV sequences were first aligned using MUSCLE v3.8.3158. Then, IQ-TREE v1.6.1259 was employed to implement the maximum-likelihood phylogenetic trees with the automated detection of the best evolutionary model (Total: GTR + F + R7; OSS: TNe + R10; CES: SYM + I + G4) using ModelFinder60 with 1,000 replicates. The final consensus trees were visualized and annotated with iTOL v6.6 (https://itol.embl.de/)61.<br>All the MAGs were taxonomically classified with Genome Taxonomy Database Toolkit (GTDB-Tk, v1.7.0)70 and annotated using the GCM online tool (https://gctype.wdcm.org/)71. Secondary metabolism analysis was performed using antiSMASH (version 6.1.1)72 with the default parameters. |
| Data analysis | R-software was used for data analysis with different packages.<br>Microbial biomarkers with statistical differences among groups were identified using LEfSe analysis according to the set screening criteria LDA score ≥4.0. FAPROTAX56 and PICRUSt257 were used to predict the functions of ASVs. |

For manuscripts utilizing custom algorithms or software that are central to the research but not yet described in published literature, software must be made available to editors and reviewers. We strongly encourage code deposition in a community repository (e.g. GitHub). See the Nature Portfolio guidelines for submitting code & software for further information.

## Data

Policy information about availability of data

All manuscripts must include a data availability statement. This statement should provide the following information, where applicable:

- Accession codes, unique identifiers, or web links for publicly available datasets
- A description of any restrictions on data availability
- For clinical datasets or third party data, please ensure that the statement adheres to our policy

Data will be made available on request

## Research involving human participants, their data, or biological material

Policy information about studies with human participants or human data. See also policy information about sex, gender (identity/presentation), and sexual orientation and race, ethnicity and racism.

| | |
|---|---|
| Reporting on sex and gender | *Use the terms sex (biological attribute) and gender (shaped by social and cultural circumstances) carefully in order to avoid confusing both terms. Indicate if findings apply to only one sex or gender; describe whether sex and gender were considered in study design; whether sex and/or gender was determined based on self-reporting or assigned and methods used. Provide in the source data disaggregated sex and gender data, where this information has been collected, and if consent has been obtained for sharing of individual-level data; provide overall numbers in this Reporting Summary. Please state if this information has not been collected. Report sex- and gender-based analyses where performed, justify reasons for lack of sex- and gender-based analysis.* |
| Reporting on race, ethnicity, or other socially relevant groupings | *Please specify the socially constructed or socially relevant categorization variable(s) used in your manuscript and explain why they were used. Please note that such variables should not be used as proxies for other socially constructed/relevant variables (for example, race or ethnicity should not be used as a proxy for socioeconomic status). Provide clear definitions of the relevant terms used, how they were provided (by the participants/respondents, the researchers, or third parties), and the method(s) used to classify people into the different categories (e.g. self-report, census or administrative data, social media data, etc.) Please provide details about how you controlled for confounding variables in your analyses.* |
| Population characteristics | *Describe the covariate-relevant population characteristics of the human research participants (e.g. age, genotypic information, past and current diagnosis and treatment categories). If you filled out the behavioural & social sciences study design questions and have nothing to add here, write "See above."* |
| Recruitment | *Describe how participants were recruited. Outline any potential self-selection bias or other biases that may be present and how these are likely to impact results.* |
| Ethics oversight | *Identify the organization(s) that approved the study protocol.* |

Note that full information on the approval of the study protocol must also be provided in the manuscript.

# Field-specific reporting

Please select the one below that is the best fit for your research. If you are not sure, read the appropriate sections before making your selection.

☐ Life sciences ☐ Behavioural & social sciences ☒ Ecological, evolutionary & environmental sciences

For a reference copy of the document with all sections, see nature.com/documents/nr-reporting-summary-flat.pdf

# Ecological, evolutionary & environmental sciences study design

All studies must disclose on these points even when the disclosure is negative.

| | |
|---|---|
| Study description | Seven soil samples (~500 g for each) were collected on 21st June 2021 from two different sites (5–20 cm depth) in the Gurbantunggut Desert, Xinjiang, northwestern China (Site 1: 44°53′9″N, 86°18′21″E; Site 2: 45°15′59″N; 85°2′21″E; Supplementary Fig. 9). One bulk soil was collected from each sampling site. The rhizosphere soils of Haloxylon ammodendron and Calligonum leucocladum (n = 2) were sampled from Site 1, while the rhizosphere soils of Haloxylon ammodendron, Tamarix chinensis and Populus euphratica (n = 3) were taken from Site 2 (Supplementary Fig. 10). The samples for DNA extraction were immediately placed on dry ice and transported to the laboratory and stored at −80 ? until further processing, and the samples for cultivation were placed on ice and kept at 4 ? until the isolation procedure was performed. |
| Research sample | Plant and soil types are the two main drivers of the soil microbial community42. Thus, prior to implementing large-scale cultivation (culturomics), a pre-experiment was carried out for the pilot screening of soil samples. An amount of 10.0 g of each soil was suspended in sterile phosphate buffer saline with glass beads (3 mm diameter) to make the final volume 100 ml and kept in a rotary shaker at 30 ?, 180 rpm for 1 h. The suspensions were 10-fold serially diluted, and aliquots of 100 µl of dilutions 10−2–10−5 were spread onto Reasoner's 2A agar (R2A) and tryptic soy agar (TSA). After 2.5–5 days of incubation at 30 ?, all the plates were imaged |

and compared comprehensively (Supplementary Fig. 10). After that, the rhizosphere soil of Calligonum leucocladum (Site 1) was selected on the basis of visually highest colony forming unit and morphological diversity of culturable bacteria on agar plates, as well as the associated bulk soil as the subsequent experimental soil samples (n = 2).

| | |
|---|---|
| Sampling strategy | Plant and soil types are the two main drivers of the soil microbial community. Thus, prior to implementing large-scale cultivation (culturomics), a pre-experiment was carried out for the pilot screening of soil samples. An amount of 10.0 g of each soil was suspended in sterile phosphate buffer saline with glass beads (3 mm diameter) to make the final volume 100 ml and kept in a rotary shaker at 30 ?, 180 rpm for 1 h. The suspensions were 10-fold serially diluted, and aliquots of 100 µl of dilutions 10−2–10−5 were spread onto Reasoner's 2A agar (R2A) and tryptic soy agar (TSA). After 2.5–5 days of incubation at 30 ?, all the plates were imaged and compared comprehensively (Supplementary Fig. 10). After that, the rhizosphere soil of Calligonum leucocladum (Site 1) was selected on the basis of visually highest colony forming unit and morphological diversity of culturable bacteria on agar plates, as well as the associated bulk soil as the subsequent experimental soil samples (n = 2). |
| Data collection | All the authors contributed to the data collection and analysis except Mukhtiar Ali. The authors Shuai Li and Lei Dong process the data via different methods as mentioned in the article section DNA extraction and PacBio SMRT sequencing, PacBio SMRT sequencing data processing, Full-length 16S rRNA sequencing data analysis, and Shotgun metagenomic sequencing, data processing, and analysis. |
| Timing and spatial scale | Sample were collected in June, 2021.<br>Laboratory study was conducted directly after 5 days of sampling. Five days were taken by the transportation activity from Xinjiang to Guangzhou.<br>Culture study was carried out for 16 days each. |
| Data exclusions | No data were excluded |
| Reproducibility | All the experiments to repeat the experiment were successful |
| Randomization | The samples were collected randomly from both rhizosphere and bulk and each was mixed thoroughly to remove any biased errors. |
| Blinding | Blinding was not relevant to this study because the previous reports explain clearly the data collection and processing due to which all the data was clear and reported properly in the manuscript. |

Did the study involve field work? ☒ Yes ☐ No

## Field work, collection and transport

| | |
|---|---|
| Field conditions | Seven soil samples (~500 g for each) were collected on 21st June 2021 from two different sites (5–20 cm depth) in the Gurbantunggut Desert, Xinjiang, northwestern China. |
| Location | Site 1: 44°53′9″N, 86°18′21″E; Site 2: 45°15′59″N; 85°2′21″E |
| Access & import/export | Plant and soil types are the two main drivers of the soil microbial community42. Thus, prior to implementing large-scale cultivation (culturomics), a pre-experiment was carried out for the pilot screening of soil samples. An amount of 10.0 g of each soil was suspended in sterile phosphate buffer saline with glass beads (3 mm diameter) to make the final volume 100 ml and kept in a rotary shaker at 30oC, 180 rpm for 1 h. The suspensions were 10-fold serially diluted, and aliquots of 100 µl of dilutions 10−2–10−5 were spread onto Reasoner's 2A agar (R2A) and tryptic soy agar (TSA). After 2.5–5 days of incubation at 30 ?, all the plates were imaged and compared comprehensively. After that, the rhizosphere soil of Calligonum leucocladum (Site 1) was selected on the basis of visually highest colony forming unit and morphological diversity of culturable bacteria on agar plates, as well as the associated bulk soil as the subsequent experimental soil samples (n = 2). |
| Disturbance | No disturbance occurred during the experiment |

# Reporting for specific materials, systems and methods

We require information from authors about some types of materials, experimental systems and methods used in many studies. Here, indicate whether each material, system or method listed is relevant to your study. If you are not sure if a list item applies to your research, read the appropriate section before selecting a response.

## Materials & experimental systems

| n/a | Involved in the study |
|---|---|
| ☒ | ☐ Antibodies |
| ☒ | ☐ Eukaryotic cell lines |
| ☒ | ☐ Palaeontology and archaeology |
| ☒ | ☐ Animals and other organisms |
| ☒ | ☐ Clinical data |
| ☒ | ☐ Dual use research of concern |
| ☒ | ☐ Plants |

## Methods

| n/a | Involved in the study |
|---|---|
| ☒ | ☐ ChIP-seq |
| ☒ | ☐ Flow cytometry |
| ☒ | ☐ MRI-based neuroimaging |
